# Alternaric acid: formal synthesis and related studies

**DOI:** 10.3762/bjoc.9.19

**Published:** 2013-01-24

**Authors:** Michael C Slade, Jeffrey S Johnson

**Affiliations:** 1 Caudill Laboratories, Department of Chemistry, University of North Carolina at Chapel Hill, Chapel Hill, NC 27599-3290, USA

**Keywords:** formal synthesis, multicomponent coupling, natural products, silyl glyoxylates

## Abstract

A silyl glyoxylate three-component-coupling methodology has been exploited to achieve a formal synthesis, an analogue to an intermediate in a distinct formal synthetic route, and a third (unique) approach to the natural product alternaric acid. Highlighted in this study is the versatility of silyl glyoxylates to engage a variety of nucleophile and electrophile pairs to provide wide latitude in the approach to complex molecule synthesis.

## Introduction

**Scheme 1:**
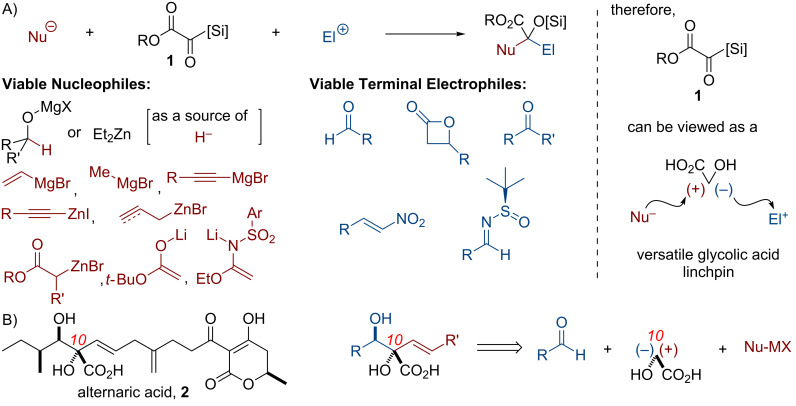
**(**A) Silyl glyoxylates as versatile reagents for three-component coupling reactions: representative nucleophiles and electrophiles. (B) Alternaric acid as a potential application of a silyl glyoxylate-enabled three-component coupling reaction.

The rapid development of molecular complexity from simple starting materials is an important goal in modern synthetic organic chemistry. In this context, streamlined one-pot transformations, cascade reactions, and multicomponent couplings have emerged as enabling tools for the synthesis of complex molecules [1–2]. Our laboratory [3–16] and others have developed [17] and employed [18–19] silyl glyoxylates **1** in a variety of synthetic endeavors, both in natural-product synthesis and synthetic methodologies [20]. Key to their use in a variety of contexts is the ability of silyl glyoxylates to function as linchpin synthons for geminal coupling of nucleophile/electrophile pairs at a glycolic acid subunit (Scheme 1A), which allows the rapid build-up of molecular complexity. Alternaric acid (**2**) [21–23] is an antifungal and phytotoxic natural product, which bears a substituted glycolic acid in the functionally and stereochemically dense core of the molecule; the potential application of silyl glyoxylate technology emerged as an attractive starting point for synthetic planning (Scheme 1B). This paper summarizes our synthetic work in this arena, which culminated in a formal synthesis, an analogue of another formal synthesis, and a unique approach to the target; each of the routes was enabled by distinct coupling partners.

**Scheme 2:**
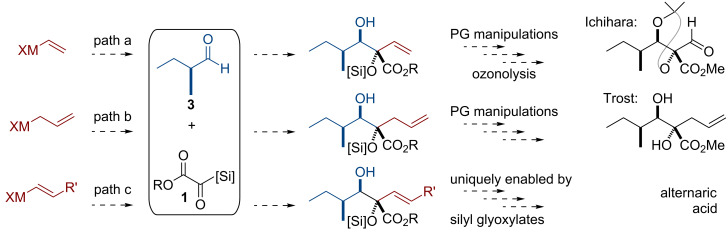
Potential applications of silyl glyoxylate couplings and precedent synthetic intermediates toward the synthesis of alternaric acid.

Alternaric acid is a particularly interesting target to demonstrate the utility of silyl glyoxylates, as it has been the subject of one total synthesis [24], one formal synthesis [25], and a potential application of an asymmetric glycolate aldol methodology [26]. These precedents serve as fruitful comparison points for application of a silyl glyoxylate three-component coupling methodology. Scheme 2 highlights three potential avenues toward the natural product and their precedents, by using the readily available (*S*)-2-methylbutanal (**3**) [27] and silyl glyoxylates **1** as two of the three key components for a coupling reaction.

## Results and Discussion

Synthetic studies were initiated to explore paths a and b in Scheme 2, given the perceived rapidity with which the known intermediates could be intercepted after the proposed three-component coupling reactions. These initial studies revealed a limitation to this approach, inherent in the use of aldehyde **3** as a coupling partner: inherently poor Felkin–Anh facial selectivity with respect to the aldehyde electrophile due to minor differentiation between the Et/Me groups [28–30]. In all cases, the facial selectivity was rather poor, i.e., approximately 1.7:1, regardless of nucleophile, counterion, solvent, and temperature. Brief optimization efforts for each nucleophile thus focused on maximizing the coupling efficiency and *syn*-/*anti*-aldol selectivity (see Supporting Information File 1).

**Scheme 3:**
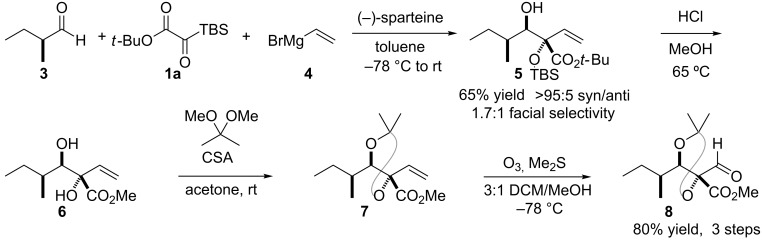
Three-component coupling with a vinyl nucleophile and elaboration to Ichihara’s aldehyde.

The optimal conditions for use of a vinyl nucleophile involved addition of a solution of vinylmagnesium bromide (**4**) and (−)-sparteine [31] in toluene to a solution of the *tert*-butyl silyl glyoxylate **1a** and (*S*)-2-methylbutanal (**3**) in toluene at −78 °C followed by warming to room temperature, which provided the three-component-coupling product **5** with excellent (>20:1) *syn-*/*anti-*aldol selectivity in 65% yield (Scheme 3). Ichihara’s aldehyde intermediate could be intercepted in three additional steps, in high overall yield. Simultaneous cleavage of the silyl ether and transesterification from the *tert*-butyl to the methyl ester in **6** was effected by warming in acidic methanol. Subsequent acetonide formation provided **7**, and ozonolysis afforded Ichihara’s aldehyde **8** (Scheme 3).

**Scheme 4:**
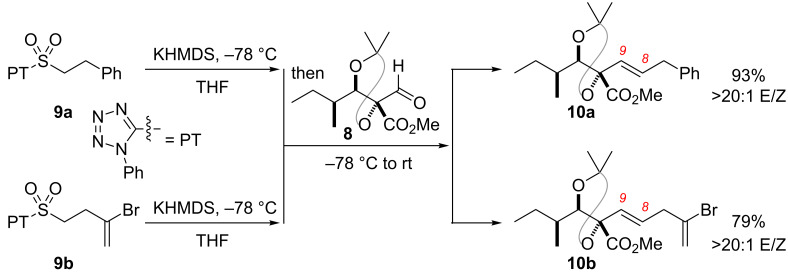
Modified Julia olefination as a step-efficient alternative endgame strategy.

Interception of this intermediate thus constituted a formal synthesis; the precedent for the C8–C9 olefination involved a classical, three-step Julia olefination sequence [24]. To demonstrate proof-of-concept for a more step-efficient endgame, test substrates were prepared for exploration of a modified Julia olefination [32]. As shown in Scheme 4, the phenyltetrazole heteroaromatic core in sulfones **9a** and **9b** provided excellent *E-*/*Z-* selectivity for formation of the C8–C9 olefin under typical modified Julia conditions with no optimization necessary. In particular, the vinyl bromide functional handle in **10b** provides a potential avenue for elaboration to the natural product.

**Scheme 5:**
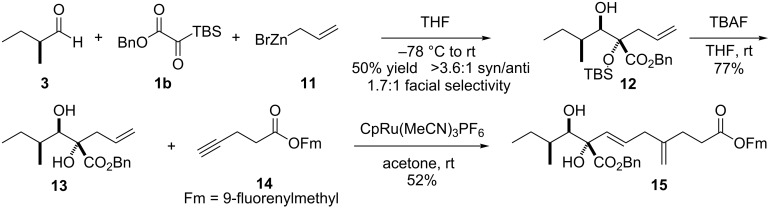
Three-component coupling with an allyl nucleophile and demonstration of successful ruthenium-catalyzed Alder–ene approach.

With the promise of the approach thus demonstrated involving the use of the vinyl nucleophile, attention shifted toward exploration of the allyl nucleophile. The best conditions for the use of an allyl nucleophile involved the addition of allylzinc bromide (**11**) in THF to a THF solution of benzyl silyl glyoxylate **1b** and (*S*)-2-methylbutanal (**3**) at 0 °C followed by warming to room temperature (Scheme 5). In the event, the three-component coupling product **12** was in 50% combined yield of all four possible diastereomers: 3.6:1 *syn-*/*anti-*selectivity and ~1.7:1 facial selectivity were observed. Thus, under these conditions both the control of enolate geometry as well as facial selectivity with respect to the aldehyde were incomplete. The four diastereomers could only be separated into *syn*/*anti* sets; within each set, the Felkin/anti-Felkin diastereomers could not be separated.

As with three-component coupling product **5**, advancement of intermediate **12** proved straightforward (Scheme 5). Deprotection of the silyl ether with TBAF afforded diol **13**, which is a benzyl ester analogue of one of Trost’s substrates employed in the ruthenium-catalyzed Alder–ene reaction [25]. It too proved to be a successful substrate for the reaction with alkyne **14**, affording the 1,4-diene product **15** in 52% yield. This sequence thus demonstrated a second avenue for successful exploitation of a silyl glyoxylate coupling methodology to achieve a step-efficient approach toward the assembly of the carbon skeleton of alternaric acid.

The two approaches described above both highlighted an important limitation to the use of (*S*)-2-methylbutanal (**3**) as the third component in the silyl glyoxylate-based three-component coupling reaction: while this aldehyde directly affords the substructure of the natural product target, it is unable to adequately control the facial selectivity of the approach of the glycolate enolate revealed after nucleophile addition/[1,2]-Brook [33] rearrangement. Moreover, attempts to achieve separation of the resultant diastereomers at all synthetic intermediates in these two routes were unsuccessful. Thus, attention shifted to address the stereochemical issue.

**Scheme 6:**
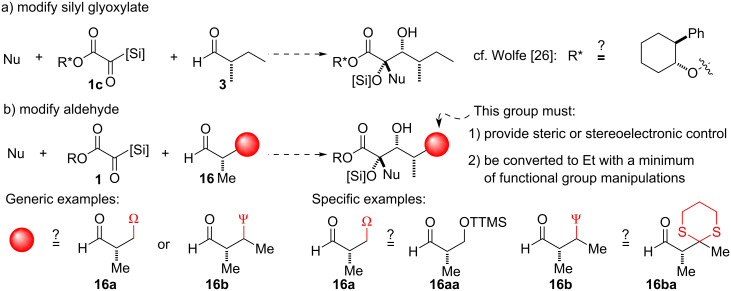
Approaches considered to address the stereochemical issue.

Various approaches were considered to achieve a higher level of stereoselection in the three-component coupling reaction, which are summarized in Scheme 6 [34]. In light of the elegant precedent for overriding the moderate substrate bias from (*S*)-2-methylbutanal (**3**) [26], auxiliary modification of the silyl glyoxylate structure to generalized type **1c** could be envisioned. As hydrolysis of an ester would be required as a late-stage deprotection in any silyl glyoxylate-based approach, this modification would represent a relatively minor departure from ideality in the form of additional concession steps [35]. Alternatively, modification of the aldehyde partner, as in generalized type **16**, was also considered. For this purpose, any stereocontrolling element (Ω or Ψ in aldehyde types **16a** and **16b**, respectively) employed should meet the additional requirement that it be easily converted to a simple ethyl group to minimize the number of concession steps.

**Scheme 7:**
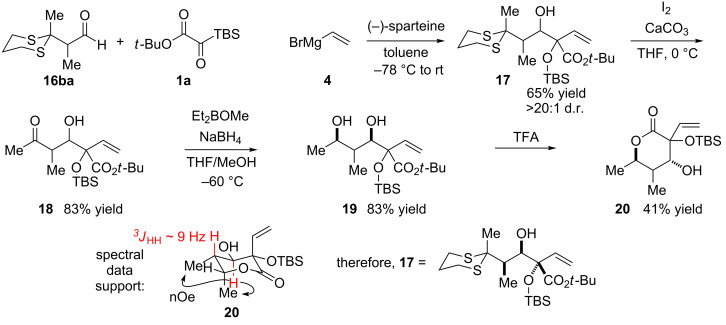
Use of a dithiane moiety to excert stereochemical control in the three-component coupling reaction and supporting evidence for its nature as a nonchelating R_L_.

The auxiliary approach using silyl glyoxylates **1c** ([Si] = TES or TBS, Scheme 6) proved to be suboptimal: despite the successful formation of the desired three-component coupling product, yields were low and poor stereochemical control was observed. Likewise, even the extreme steric demand of the tris(trimethylsilyl) group in aldehyde **16aa** was insufficient for adequate stereocontrol in the three-component coupling reaction [34]. An additional branch point in the carbon backbone, such as in **16b**, was deemed necessary. The 1,3-dithiane group in aldehyde **16ba** was conceived as a promising candidate for a stereocontrolling element due to its large size and the wealth of precedents for single-step desulfurization to alkanes [36–40]. The racemic synthesis of the requisite aldehyde proved straightforward (see Supporting Information File 1). Most importantly, in initial three-component coupling reactions with vinyl nucleophile **4** and silyl glyoxylate **1a** under previously optimized conditions, high efficiency was achieved along with excellent (>20:1) stereochemical control for the formation of three-component-coupling product **17** (Scheme 7) [41–44]. To verify that the dithiane was acting in the desired fashion, and to rule out chelation from one of the Lewis basic sulfur atoms, derivatization to a lactone was carried out. The dithiane was cleaved to the ketone **18**, which underwent a 1,3-*syn*-selective reduction [45]. The resultant diol **19** was subjected to acidic conditions to effect cleavage of the *tert*-butyl ester and lactonization to afford **20**. The NOESY and coupling-constant data of **20** was consistent with the role of the dithiane in **16ba** as a nonchelating R_L_ group that led to Felkin selectivity in the three-component coupling reaction.

**Scheme 8:**
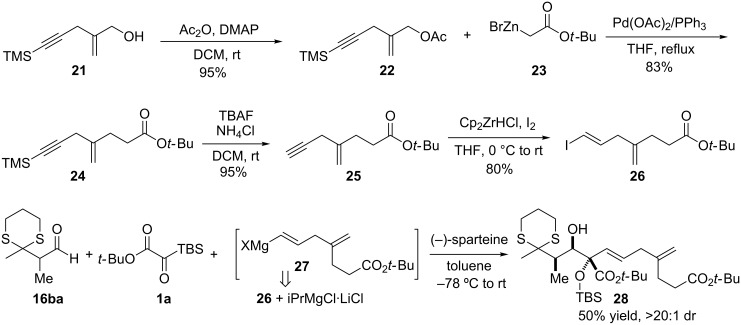
Synthesis of a vinyl iodide for nucleophile generation and its use in a three-component coupling reaction.

The complete diastereochemical control exerted by the dithiane moiety of the aldehyde **16ba** provided the impetus for exploring the use of a functionalized vinyl nucleophile in the three-component coupling reaction. Use of a more complex nucleophile would maintain the convergence of the overall synthesis, which was deemed important because (1) the route to the aldehyde component was becoming more involved; and (2) one or more additional steps for the removal of the directing group would be required. Thus, we developed a synthesis of a nucleophile that would allow the vast majority of the alternaric acid carbon skeleton to be installed through the three-component coupling reaction (Scheme 8). It began from the known allylic alcohol **21** [46], which was acetylated to afford ester **22** as prelude for reaction as a π-allyl electrophile with the Reformatsky reagent **23** derived from *tert*-butyl bromoacetate. The TMS-alkyne in **24** was deprotected with buffered TBAF to afford free alkyne **25**, and the vinyl iodide **26** was generated by hydrozirconation/iodination of the free alkyne with Schwartz’s reagent [47]. The vinyl nucleophile **27** could be generated by Knochel’s Mg/I exchange [48] and employed successfully in the three-component-coupling reaction with silyl glyoxylate **1a** and aldehyde **16ba** to assemble **28**, which contains the bulk of the carbon skeleton of alternaric acid. Remarkably, this highly convergent coupling allows the majority of the carbon backbone of the natural product to be assembled in a single complexity-building step.

With this gratifying result, a third distinct route to alternaric acid was enabled. Most importantly, this provides the first example of such a highly functionalized nucleophile being used in a silyl glyoxylate based three-component coupling reaction. Remaining tasks for the complete formation of the natural product include desulfurization [49], deprotection [50], and appendage of the pyrone moiety [24].

## Conclusion

In conclusion, we have described the application of silyl glyoxylate three-component-coupling reactions as the central feature of three distinct approaches to the total synthesis of alternaric acid. By judicious choice of coupling partner and reaction conditions, it has been possible to achieve a formal synthesis, an analogous formal synthesis via an alternative route, and significant progress toward a third distinct route reliant on a highly functionalized nucleophile/electrophile combination for the construction of the majority of the natural product in a single step. In particular, this underscores the unique utility of silyl glyoxylates to serve as crucial linchpins for the coupling of a variety of nucleophile/electrophile pairs at a glycolic acid junction for the rapid development of molecular complexity.

## Supporting Information

Contains additional tables and schemes for the three-component coupling reactions and approaches to address the stereochemical problem. Also contains experimental procedures, characterization, and spectral data.

File 1 Additional data.

## References

[1] Nicolaou K C, Hale C R H, Nilewski C, Ioannidou H A (2012). Chem Soc Rev.

[2] (2009). Rapid complexity generation in natural product total synthesis. Chem Soc Rev.

[3] Nicewicz D A, Johnson J S (2005). J Am Chem Soc.

[4] Linghu X, Satterfield A D, Johnson J S (2006). J Am Chem Soc.

[5] Nicewicz D A, Brétéché G, Johnson J S, Bryan C, Lautens M (2008). Org Synth.

[6] Nicewicz D A, Satterfield A D, Schmitt D C, Johnson J S (2008). J Am Chem Soc.

[7] Greszler S N, Johnson J S (2009). Org Lett.

[8] Greszler S N, Johnson J S (2009). Angew Chem, Int Ed.

[9] Schmitt D C, Johnson J S (2010). Org Lett.

[10] Steward K M, Johnson J S (2010). Org Lett.

[11] Boyce G R, Johnson J S (2010). Angew Chem, Int Ed.

[12] Greszler S N, Malinowski J T, Johnson J S (2010). J Am Chem Soc.

[13] Greszler S N, Malinowski J T, Johnson J S (2011). Org Lett.

[14] Schmitt D C, Lam L, Johnson J S (2011). Org Lett.

[15] Boyce G R, Liu S, Johnson J S (2012). Org Lett.

[16] Schmitt D C, Malow E J, Johnson J S (2012). J Org Chem.

[17] Bolm C, Kasyan A, Heider P, Saladin S, Drauz K, Günther K, Wagner C (2002). Org Lett.

[18] Lettan R B, Galliford C V, Woodward C C, Scheidt K A (2009). J Am Chem Soc.

[19] Yao M, Lu C D (2011). Org Lett.

[20] Boyce G R, Greszler S N, Johnson J S, Linghu X, Malinowski J T, Nicewicz D A, Satterfield A D, Schmitt D C, Steward K M (2012). J Org Chem.

[21] Brian P W, Curtis P J, Hemming H G, Unwin C H, Wright J M (1949). Nature.

[22] Brian P W, Curtis P J, Hemming H G, Jefferys E G, Unwin C H, Wright J M (1951). J Gen Microbiol.

[23] Bartels-Keith J R (1960). J Chem Soc.

[24] Tabuchi H, Hamamoto T, Miki S, Tejima T, Ichihara A (1994). J Org Chem.

[25] Trost B M, Probst G D, Schoop A (1998). J Am Chem Soc.

[26] Giampietro N C, Kampf J W, Wolfe J P (2009). J Am Chem Soc.

[27] Anelli P L, Montanari F, Quici S, Nonoshita K, Yamamot H (1990). Org Synth.

[R28] 28Our results are consistent with the additions of nucleophiles to **3** (cf. [24–26]). For rare, successful examples of Me/Et differentiation, see [29–30].

[29] Evans D A, Kozlowski M C, Burgey C S, MacMillan D W C (1997). J Am Chem Soc.

[30] Copeland G T, Miller S J (2001). J Am Chem Soc.

[R31] 31The precise role of the (−)-sparteine is uncertain at this time. It is possible that the Lewis basic nitrogens chelate the magnesium, facilitating the reversibility of the aldolization step leading to high *syn-*selectivity. See [20], footnote 42.

[32] Blakemore P R (2002). J Chem Soc, Perkin Trans 1.

[33] Brook A G (1974). Acc Chem Res.

[R34] 34The results of efforts with silylglyoxylates **1c**, and other specific examples for the identities of Ω and Ψ, were also pursued. See Supporting Information File 1 for additional details.

[35] Gaich T, Baran P S (2010). J Org Chem.

[36] Yang T-K, Lee D-S, Haas J (2006). Raney Nickel. e-EROS Encyclopedia of Reagents for Organic Synthesis.

[37] Back T G, Baron D L, Yang K (1993). J Org Chem.

[38] Chan M C, Cheng K M, Ho K M, Ng C T, Yam T M, Wang B S L, Luh T Y (1988). J Org Chem.

[39] Becker S, Fort Y, Vanderesse R, Caubere P (1988). Tetrahedron Lett.

[40] Becker S, Fort Y, Caubere P (1990). J Org Chem.

[R41] 41Aldehyde **16ba** has previously been used in aldol reactions; no comment on the diastereoselectivity was made, likely because it was immaterial in those applications (see [42–43]).

[42] Kim H, Baker J B, Lee S-U, Park Y, Bolduc K L, Park H-B, Dickens M G, Lee D-S, Kim Y, Kim S H (2009). J Am Chem Soc.

[43] Kim H, Baker J B, Park Y, Park H B, DeArmond P D, Kim S H, Fitzgerald M C, Lee D-S, Hong J (2010). Chem–Asian J.

[44] Ward D E, Kazemeini A (2012). J Org Chem.

[45] Chen K-M, Gunderson K G, Hardtmann G E, Prasad K, Repic O, Shapiro M J (1987). Chem Lett.

[46] Renaud J-L, Aubert C, Malacria M (1999). Tetrahedron Lett.

[47] Reichard H A, Rieger J C, Micalizio G C (2008). Angew Chem, Int Ed.

[48] Ren H, Krasovskiy A, Knochel P (2004). Org Lett.

[R49] 49Initial attempts to effect this transformation in a model system have demonstrated that this may not be as straightforward as anticipated. See Supporting Information File 1 for details.

[R50] 50Likewise, this may prove more difficult than anticipated; acid-promoted hydrolysis of the *tert*-butyl esters and silyl ether was planned, due to the success of such a transformation with the simpler substrate **5**, as in Scheme 3. However, substrate **28** tends to decompose when treated under a variety of acidic conditions.

