# Supplementary material for: Alternaric acid: formal synthesis and related studies
Source: Beilstein J Org Chem. 2013 Jan 24;9:166–72. doi: 10.3762/bjoc.9.19 (PMC3566800; doi:10.3762/bjoc.9.19)
Supplement: File 1 — Additional data. [file Beilstein_J_Org_Chem-09-166-s001.pdf]

Supporting Information

for

Alternaric acid: formal synthesis and related studies

Michael C. Slade and Jeffrey S. Johnson*

Address: Caudill Laboratories, Department of Chemistry, University of North Carolina at Chapel Hill, Chapel Hill, NC 27599-3290, USA

Email: Jeffrey S. Johnson - jsj@unc.edu

*Corresponding author

Additional data

CONTENTS

**General methods and materials**	**S-2**
**Brief optimization tables for vinyl-/allyl-nucleophile 3-component couplings**	**S-2**
**Discussion of the stereochemical issue**	**S-4**
**Synthesis of dithiane aldehyde 16ba**	**S-6**
**Alternative methods for nucleophile generation/3-C-C**	**S-6**
**Discussion of desulfurization issues**	**S-7**
**Experimental**	**S-8**
**References**	**S-24**
**Spectra**	**S-25**

Materials and Methods: General. Infrared (IR) spectra were obtained using a Jasco 260 Plus Fourier transform infrared spectrometer. Proton and carbon nuclear magnetic resonance spectra (^{1}H NMR and ^{13}C NMR) were recorded on a Bruker 300, Avance 400, DRX 400, or 600 MHz (^{1}H NMR at 300, 400, or 600 MHz and ^{13}C NMR at 100 or 150 MHz) spectrometer with solvent resonance as the internal standard (^{1}H NMR: $CDCl_3$ at 7.26 ppm, ^{13}C NMR: $CDCl_3$ at 77.0 ppm). ^{1}H NMR data are reported as follows: chemical shift, multiplicity (app = apparent, s = singlet, br s = broad singlet, d = doublet, dd = doublet of doublets, dt = doublet of triplets, t = triplet, q = quartet, m = multiplet), coupling constants (Hz), and integration. Mass spectra were obtained using a Micromass Quattro II (triple quad) instrument with nanoelectrospray ionization. Analytical thin layer chromatography (TLC) was performed on Sorbent Technologies Silica G 0.20 mm silica gel plates. Visualization was accomplished with UV light, aqueous basic potassium permanganate solution ($KMnO_4$), or aqueous ceric ammonium molybdate solution (CAM) followed by heating. Flash column chromatography was performed using Silia-P flash silica gel (40–63 μm) purchased from Silicycle. Ozonolyses were performed with O_3 produced by a Yanco Industries Ozone Services model OL80B ozonator. Yield refers to isolated yield of analytically pure material unless otherwise noted. The diastereomer ratios reported are for crude reaction mixtures. Reactions were performed in oven- or flame-dried glassware equipped with Teflon coated stir bars in solvents that had been dried by passage through a column of neutral alumina under nitrogen prior to use, unless otherwise stated. When reagents that were purchased commercially were employed, a reference for the compound has been provided in the procedure. All other reagents were obtained from commercial sources and used as received, unless otherwise noted.

Brief optimization tables for three-component couplings

The following results were obtained in initial explorations of the various three-component coupling reactions discussed in the manuscript.

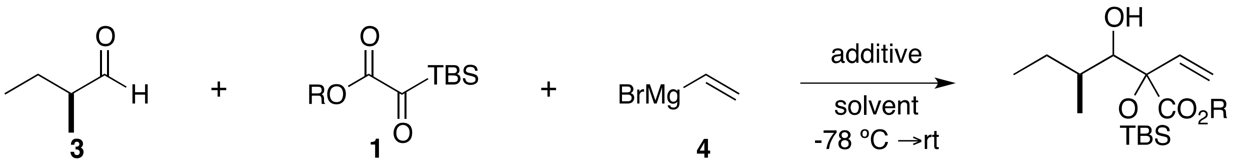

Table 1: Optimization of the vinyl nucleophile three-component coupling.

Entry	Silyl Glyoxylate	Solvent	Additive	Yield (%)[a]	dr
1	**1a**; R = *t*-Bu	THF	(−)-sparteine	40	1.4:1
2	**1a**	Toluene	(−)-sparteine	65	1.7:1
3	**1a**	THF	---	42	1.3:1[b]
4	**1a**	Toluene	---	<40	1.4:1[b]
5	**1b**; R = Bn	Toluene	(−)-sparteine	39	1.6:1

[a]Isolated yield after chromatography. [b]The 3rd and 4th diastereomers formed appreciably.

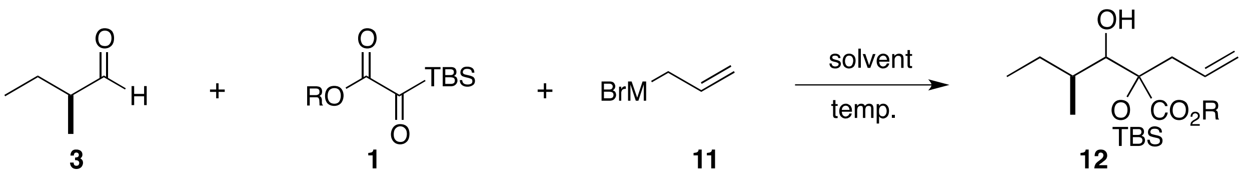

Table S-2: Optimization of the allyl nucleophile in three-component coupling.

Entry	Silyl Glyoxylate	Nucleophile	Solvent, Temp.	Result
1	**1a (*t*-Bu)**	**11-S (M = Mg)**	THF, −78 °C	"Complex mixture"[a]
2	**1b (Bn)**	**11-S**	THF, −78 °C	"Complex mixture"
3	**1a**	**11-S**	THF, −100 °C	"Complex mixture"[a]
4	**1b**	**11-S**	THF, −100 °C	20%[b] of major diast. of **12**
5	**1b**	**11 (M = Zn)**	THF, −78 °C → rt	45% total **12**,[b] 3.6:1 *syn-*/*anti-*[c]
6	**1b**	**11**	THF, 0 °C → rt	50% total **12**,[b] 3.6:1 *syn-*/*anti-*[c]

[a]Initially dismissed as such; reexamination of spectral data revealed that the isolated material was a relatively pure mixture of *all four* possible diastereomers. More of the peaks in the ^{1}H NMR of compound **12-S** (R = *t*-Bu) are resolved, as compared to product **12** (R = Bn). More of the resonances in compound **12** are coincident, which led to an initial overestimation of the chemical and stereochemical purity of this material. [b]Isolated yield. [c]Determined after isolation of the diastereomers and mixed fractions by chromatography. Only the *syn-*/*anti-*diastereomers were separable: within these, the Felkin/anti-Felkin diastereomers (~1.7:1) were inseparable.

Discussion of the stereochemical issue

The following schemes summarize the alternative approaches considered, some of which were explored to address the stereochemical issues inherent in using (*S*)-2-methylbutanal (**3**) as the terminal electrophile in three-component couplings. **Scheme S-1** provides a general overview:

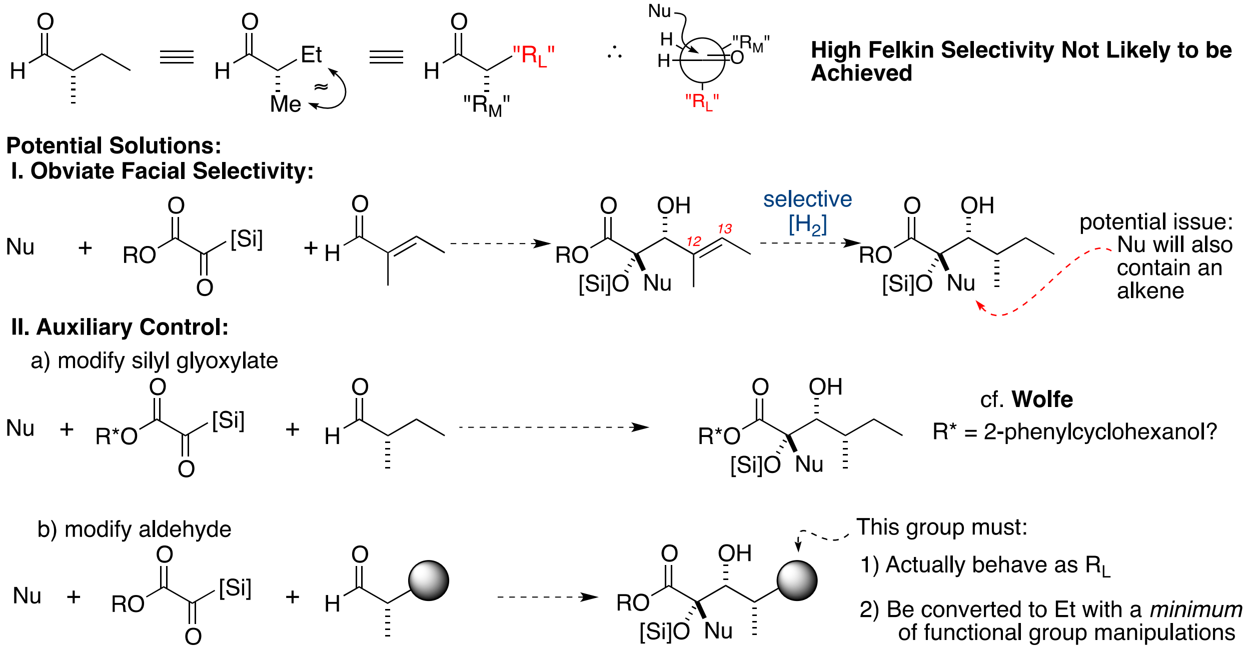

Scheme S-1: Approaches considered to address the stereochemical issue. The Wolfe citation is [1].

Approach **S-1 I** was not pursued because of the issue highlighted in Scheme **S-1**: potential chemoselectivity issues due to the unsaturation present in the nucleophilic fragment. Approach **S-1 IIa)** was investigated as summarized in **Scheme S-2** below. The approach in Scheme **S-1 IIb)** was also explored, as summarized in **Scheme S-3** to **Scheme S-7** below.

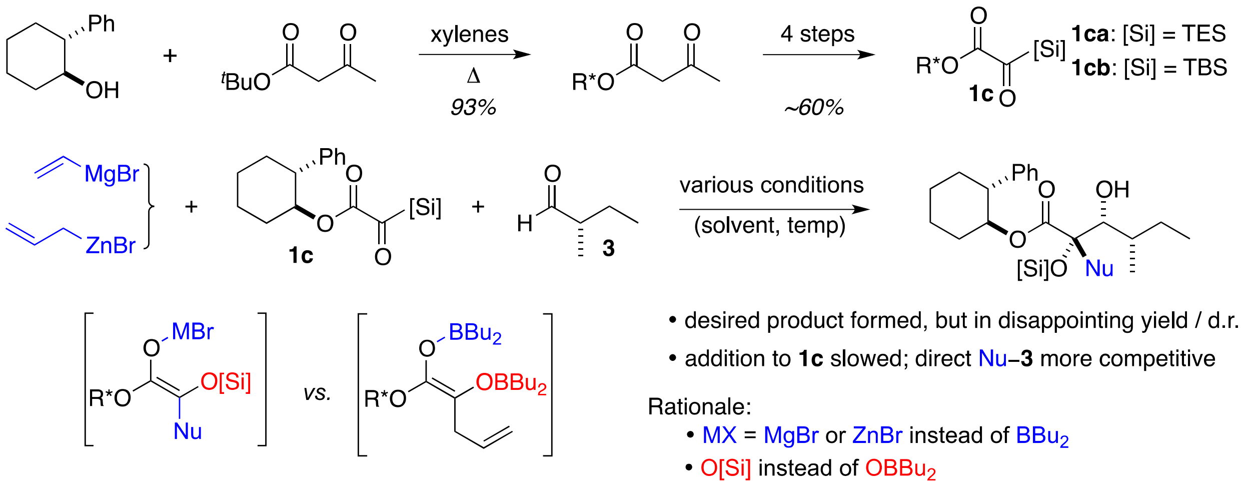

Scheme S-2: Auxiliary control via silyl glyoxylate. The four steps for completion of silyl glyoxylates **1c** were performed analogously to the standard protocol [2].

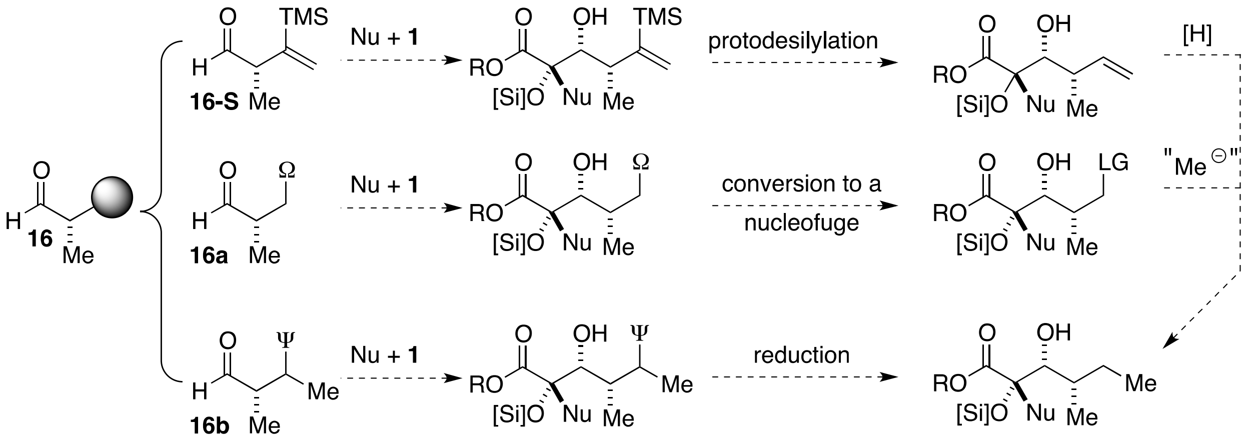

Scheme S-3: Auxiliary control via the aldehyde and projected post-coupling manipulation requirements.

Aldehyde **16-S** was developed and used by Sato and co-workers for a similar stereocontrolling purpose [3-6], but provided inadequate diastereocontrol in three-component couplings with vinylmagnesium bromide **4** and silyl glyoxylate **1a** as summarized in **Scheme S-4**, below. We attribute this to a conflict between the need to maintain cryogenic temperatures for **16-S** to exert stereochemical control, and the need to warm the three-component coupling mixture for good *E*-/*Z*-enolate geometry control.

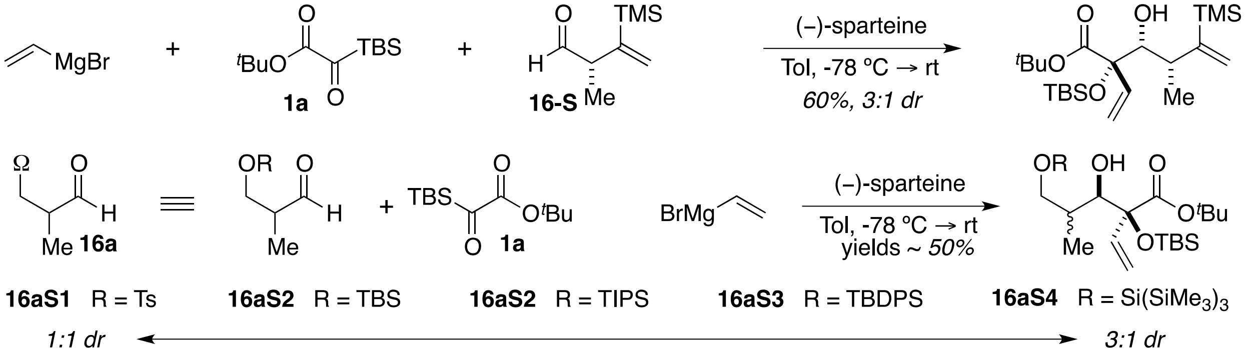

Scheme S-4: Three-component couplings with aldehydes of type **16-S**/**16a**.

The aldehydes **16a** (prepared in two steps from 2-methyl-1,3-propane diol by monoprotection and Dess-Martin oxidation) ranged in steric demand from R = tosyl to R = tris(trimethylsilyl); yields were all ~50%, while diastereoselectivity varied from 1:1 (R = tosyl) to ~3:1 (R = tris(trimethylsilyl)). From this, we judged that an extra branch point in the aldehyde backbone was needed, and aldehydes of type **16b** were investigated. The first was aldehyde **16bS1**, which was attractive for the potential to displace the mesylate with hydride (**Scheme S-5**, below). Interestingly, despite three-component couplings where oxetane formation was a relatively minor side process, subsequent derivatizations revealed this to be a major problem. Given the moderate stereochemical control (despite the small size of the mesylate), and the number of additional protection/deprotection concession steps deemed necessary to circumvent this problem, the dithiane aldehyde **16ba** was developed and pursued.

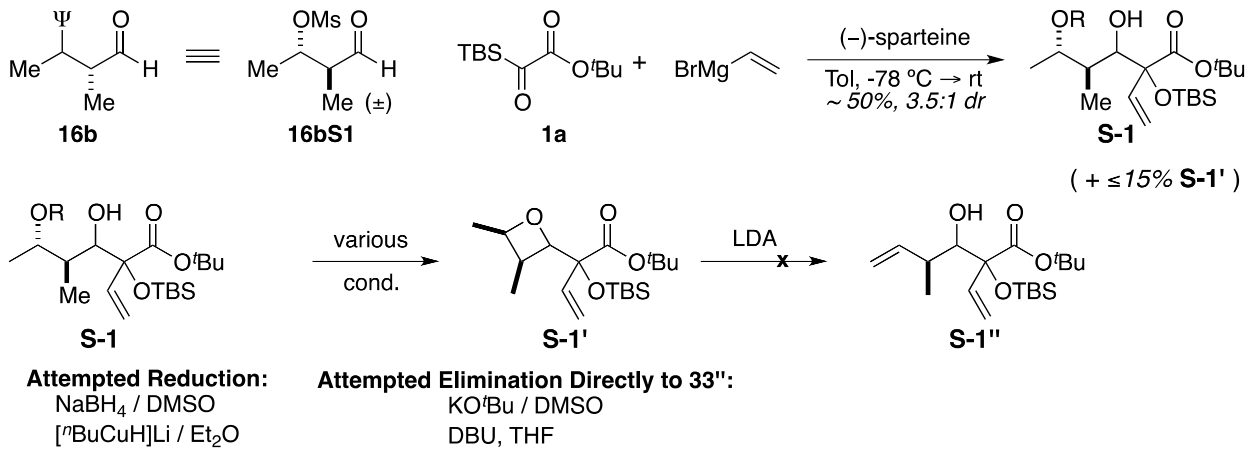

Scheme S-5: An aldehyde with a reducible group Ψ.

Synthesis of dithiane aldehyde

The route to the dithiane aldehyde **16ba** is summarized below.

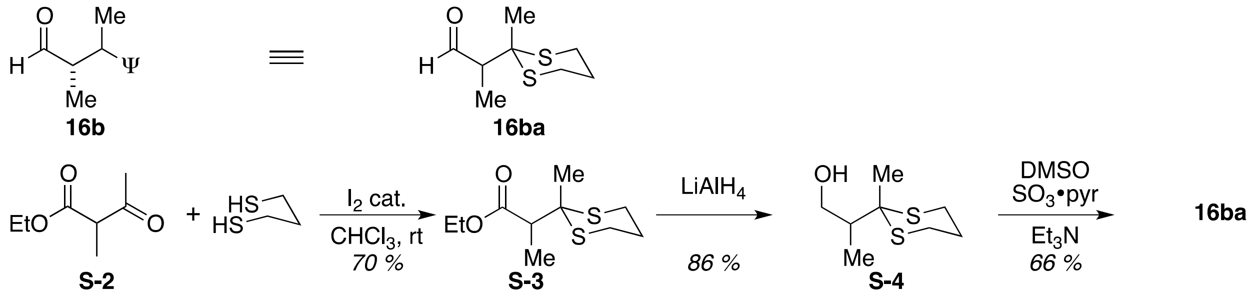

Scheme S-6: Route to the dithiane aldehyde **16ba**.

Alternative methods for nucleophile generation/3-C-C

As summarized in **Scheme S-7**, low-temperature Li/I exchange [7] (with or without transmetalation to magnesium or zinc) to provide a vinyl nucleophile **27-S** led to complex mixtures and silyl glyoxylate oligomerization in attempted three-component coupling reactions. On the other hand, generation of a vinylzinc nucleophile using the method of Wipf (with [8] or without [9] an accelerating ligand) led to a weaker nucleophile, and under these conditions silyl glyoxylate dimerization and elimination [10] was dominant.

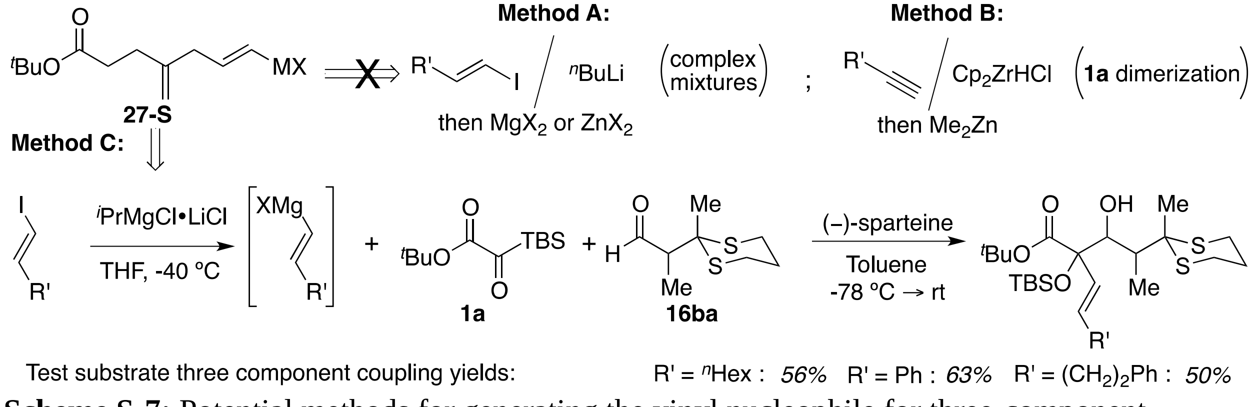

Scheme S-7: Potential methods for generating the vinyl nucleophile for three-component

coupling. All three-component coupling reactions with aldehyde **16ba** proceeded with >20:1 diastereoselectivity.

Discussion of desulfurization attempts

Due to ready availability of three-component coupling product **17**, this compound was used as a model system for the desulfurization of three-component coupling product **28** as summarized in **Scheme S-8**. Attempts with the following reagent systems were made: Raney Nickel [11], $NiCl_2/NaBH_4$ [12], $Cp_2Ni/LiAlH_4$ [13], and $Ni(OAc)_2$/NaH/^{t}AmOH [14,15]. All resulted in complex mixtures, decomposition of **17**, and/or reduction of the C8–C9 alkene.

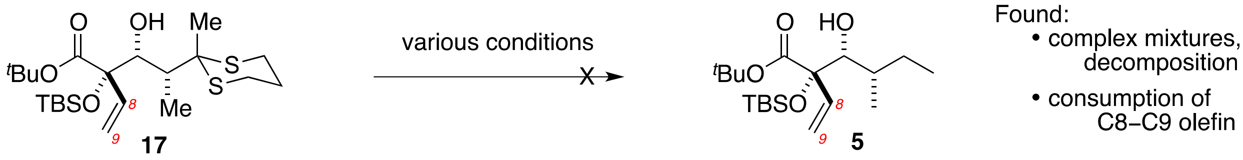

Scheme S-8: Summary of various single-step desulfurization attempts.

After those investigations proved difficult, a more stepwise approach to desulfurization of **17** was considered. As summarized in **Scheme S-9**, the dithiane could be deprotected to the ketone followed by conversion to a tosylhydrazone **S-5**, which formed cyclopentanol **S-9** instead of the desired product **5** upon attempted reduction [16]. This reaction presumably occurs via the monoalkyl diazene **S-6**, which forms radical **S-7** upon loss of dinitrogen, which then undergoes a 5-*exo* radical cyclization. Addition of >20 equivalents of strong H• donors such as *tert*-butyl mercaptan, tributyltin hydride, and tris-(trimethylsilyl)silane prior to the second stage of the reaction was attempted with the goal of attenuating this cyclization process. These H• donors were ineffective in shutting down the 5-*exo*-trig cyclization: at best, a 1:5 ratio of **S-9:5** was obtained with *tert*-butyl mercaptan. Evidently the 5-*exo*-trig intramolecular cyclization, perhaps in part due to the Thorpe-Ingold compression of this substrate, is much more rapid than intermolecular quenching of **S-8**.

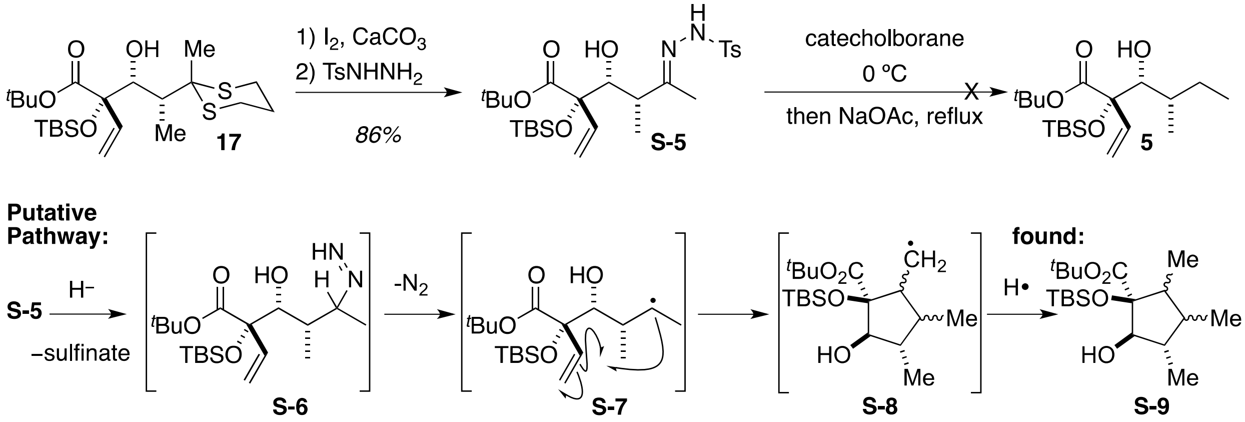

Scheme S-9: A more stepwise reduction attempt reveals a competing pathway, which may have impacted the single-step desulfurization attempts.

Experimental

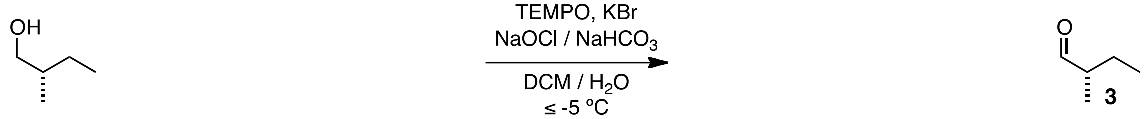

(*S*)-2-methylbutanal (3). This reaction was performed according to the reported procedure [17], with slight modifications. To a solution of (*S*)-2-methylbutanol (5 mL, 4.095 g, 46.5 mmol, 1.0 equiv) in DCM (15 mL) was added TEMPO (73 mg, 0.465 mmol, 0.01 equiv). A solution of potassium bromide (553 mg, 4.65 mmol, 0.10 equiv) in H_2O (approx. 2 mL) was added, and the resulting biphasic mixture was cooled in an ice/salt/brine bath for 15 min. Sodium bicarbonate (840 mg) was added to buffer the commercial aqueous solution of sodium hypochlorite (0.7 M, 73 mL, 51.1 mmol, 1.1 equiv), which was placed in a pressure-equalizing addition funnel. The buffered oxidant solution was added dropwise to the reaction mixture such that the total time for addition was 15–20 min (approx. 1 drop/sec). After 5 additional min, the reaction was allowed to warm to room temperature, and after an additional 5 min the layers were separated. The aqueous layer was extracted with additional DCM (3 × 25 mL), and the combined organic extracts were washed with 1 M HCl (30 mL) to which KI (approx. 300 mg) had been added (this treatment caused the organic layer to become deep red). The organic layer was subsequently washed with H_2O (30 mL), sat. aq $Na_2S_2O_3$ (30 mL), H_2O (30 mL), sat. $NaHCO_3$ (30 mL), H_2O (30 mL), brine (30 mL), and dried over Na_2SO_4. The mixture was filtered, and the bulk of the solvent was distilled at atmospheric pressure until the vapor temperature began to rise past 40 ºC. At this point, the dilute solution of aldehyde in DCM was stored in the freezer until ready for use. Prior to use, a portion of the stock aldehyde solution was distilled further using a microscale Hickman distillation tube; the desired aldehyde was distilled from the less volatile acid (from overoxidation) and aldehyde decomposition byproducts. The residual DCM content was quantified by NMR, and the aldehyde was typically used as a concentrated solution. This treatment led to aldehyde loss (early portions of its azeotrope with DCM were discarded), but it avoided the total decomposition of the aldehyde, which has an exceedingly short shelf-life when highly concentrated (~1 week in the glovebox freezer).

General procedure A: Three-component coupling reactions with vinyl Grignard

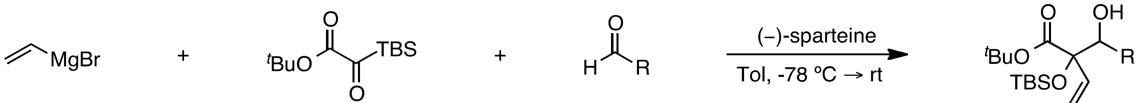

4 **1a** **3** or **16**

To a solution of (–)-sparteine (1.5 equiv) in dry toluene at −78 ºC under N_2 was added a solution of vinylmagnesium bromide (**4**, solution in THF, 1.5 equiv). A solution of silyl glyoxylate **1a** (1.0 equiv) and an aldehyde (1.5 equiv) in dry toluene was prepared and cooled to −78 ºC for ≥15 min, at which point the (–)-sparteine/Grignard solution was transferred into the reaction via cannula. After an additional 15 min, the solution was warmed to room temperature and stirred for ≥1.5 h before quenching with 10% aq AcOH (v/v). The reaction was diluted with H_2O, the layers were separated, and the aqueous layer was extracted with Et_2O three times. The combined organic extracts were washed with H_2O and brine, dried over $MgSO_4$, and concentrated *in vacuo*. The crude residue was purified by flash column chromatography using the indicated solvent

system. This procedure was used to investigate the three-component coupling reactions with all aldehydes of type **16**, although particular details for all of these will not be reproduced here.

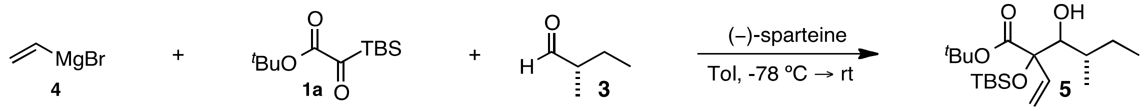

(4*S*)-*tert*-butyl 2-(*tert*-butyldimethylsilyloxy)-3-hydroxy-4-methyl-2-vinylhexanoate (5). This reaction was conducted according to General Procedure A, using vinylmagnesium bromide (**4**, 0.7 M in THF, 7.6 mL, 5.31 mmol, 2 equiv) complexed with (–)-sparteine (1.24 g, 5.31 mmol, 2 equiv) in 20 mL toluene, which was added to a solution of *t*-Bu/TBS silyl glyoxylate **1a** (650 mg, 2.65 mmol, 1.0 equiv), and (*S*)-2-methylbutanal (**3**, 5.31 mmol, 2 equiv) in 40 mL dry toluene. In this particular run, the yield was 610 mg of product as a clear, slightly yellow oil (1.7 mmol, 64% yield). The dr was > 20:1 *syn*:*anti*, ≤1.7:1 facial selectivity (**5**:**5'**). Analytical data for **5**/**5'**: **IR** (thin film, cm^{-1}): 3582, 3480, 2959, 2931, 2858, 1747, 1639, 1472, 1463, 1393, 1369, 1253, 1156, 1056, 1005, 926, 838, 780; **^{1}H NMR** (600 MHz, $CDCl_3$): *major diastereomer* **5**: δ 5.95 (dd, *J* = 17.4, 10.8 Hz, 1H), 5.40 (d, *J* = 17.4 Hz, 1H), 5.22 (d, *J* = 10.8 Hz, 1H), 3.72 (dd, *J* = 10.8, 3 Hz, 1H), 2.41 (d, *J* = 10.8 Hz, 1H), 1.75-1.65 (m, 2H), 1.49 (s, 9H), 1.28-1.22 (m, 1H), 0.93 (d, *J* = 7.2 Hz, 3H), 0.92 (s, 9H), 0.88 (t, *J* = 7.2 Hz, 3H), 0.20 (s, 3H), 0.11 (s, 3H); *resolved signals for minor diastereomer* **5'**: δ 5.39 (d, *J* = 17.4 Hz, 1H), 5.20 (d, *J* = 10.8 Hz, 1H), 3.60 (dd, *J* = 10.8, 5.4 Hz, 1H), 2.22 (d, *J* = 10.8 Hz, 1H), 0.12 (s, 3H); **^{13}C NMR** (150 MHz, $CDCl_3$): *both diastereomers*: δ 171.8, 171.6, 138.4, 138.3, 116.1, 115.8, 84.3, 83.8, 82.4, 82.3, 80.7, 78.8, 36.3, 35.3, 28.6, 27.97, 27.96, 26.37, 26.33, 24.0, 19.18, 19.14, 17.7, 13.6, 11.7, 11.4, −2.26, −2.29, −2.38 (two coincident resonances); **TLC** (5% EtOAc/hexanes), R_f 0.26 (CAM); **LRMS** (ESI): Calcd. for $C_{19}H_{38}O_4$+Na: 381.24, Found: 381.25.

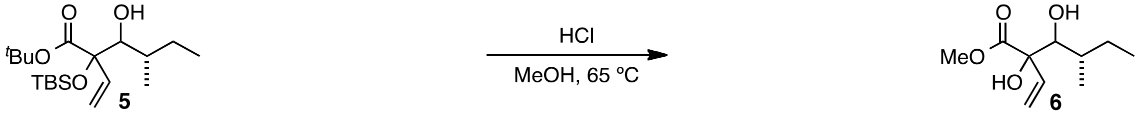

(4*S*)-methyl 2,3-dihydroxy-4-methyl-2-vinylhexanoate (6/6'). The title compound was prepared using a solution of methanolic HCl, generated as follows: to a long test tube containing methanol (~10 mL) was carefully added acetyl chloride (0.21 mL, 0.231 g, 3 mmol, 3 equiv) slowly, dropwise (audible popping sound). This solution was used to dissolve the starting material **5/5'** (359 mg, 1.0 mmol, 1.0 equiv) and transfer it to a 50 mL round-bottomed flask equipped with a stir bar, to which was subsequently affixed a reflux condenser. Another 3 mL of methanol was used to ensure complete transfer of the starting material to the reaction flask. The reaction was heated under reflux, open to air, until it was judged complete by TLC analysis (a compound of intermediate R_f, isolated once and determined to be the desilylated *tert*-butyl ester, appears *en route* to the desired product of lower R_f). Upon completion, the reaction was poured into a separatory funnel containing H_2O (~75 mL), and Et_2O (~10 mL) was added to form two layers, which were shaken and separated. The aqueous layer was extracted with Et_2O (3 × 10 mL), and the combined organic extracts were washed with H_2O (2 × 20 mL) and brine (20 mL), and dried over $MgSO_4$. Filtration and careful concentration *in vacuo* afforded 200 mg (~1 mmol, quant. yield) of the title compound **6/6'** as a clear oil, which required no further purification. Purification in an attempt to separate the diastereomers at this stage was unsuccessful. Analytical data for **6/6'**: **IR** (thin film, cm^{-1}): 3499, 2960, 2934, 2877, 1738, 1638, 1462, 1439, 1402, 1245, 1170, 1004, 932, 789; **^{1}H NMR** (600 MHz, $CDCl_3$): *major diastereomer* **20**: δ 5.91 (dd, *J* = 17.4, 10.8 Hz, 1H), 5.59 (dd, *J* = 17.4, 1.2 Hz, 1H), 5.25 (d, *J* = 10.8, 1.2 Hz, 1H), 3.95 (d, *J* = 1.8 Hz,

1H), 3.81 (s, 3H), 3.6 (br s, 1H), 2.1 (br s, 1H), 1.70-1.60 (m, 1H), 1.45-1.38 (m, 1H), 1.30-1.20 (m, 1H), 0.90-0.85 (m, 6H); *resolved signals for minor diastereomer* **20'**: δ 5.94 (dd, *J* = 17.4, 10.8 Hz, 1H), 5.57 (d, *J* = 17.4, 1.2 Hz, 1H), 5.23 (dd, *J* = 10.8, 1.2 Hz, 1H), 3.80 (s, 3H), 1.75-1.65 (m, 2H), 1.10-1.00 (m, 1H), 0.95 (d, *J* = 7.2 Hz, 3H); **^{13}C NMR** (150 MHz, $CDCl_3$): *both diastereomers*: δ 175.1, 175.0, 135.6, 135.2, 116.1, 115.9, 81.9, 81.8, 78.2, 75.8, 53.50, 53.47, 36.0, 35.3, 28.2, 22.8, 17.4, 12.8, 11.8, 11.5; **TLC** (20% EtOAc/hexanes), R_f 0.21 (CAM only); **LRMS** (ESI): Calcd. for $C_{10}H_{18}O_4$+Na: 225.11, Found: 225.12.

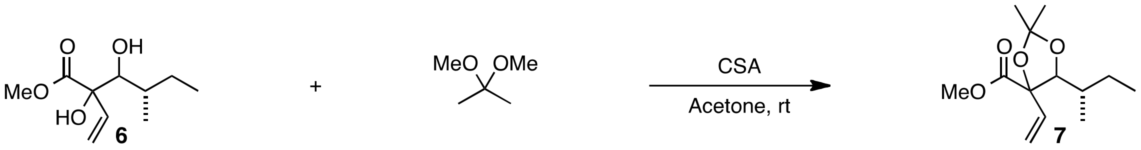

Methyl 5-((*S*)-*sec*-butyl)-2,2-dimethyl-4-vinyl-1,3-dioxolane-4-carboxylate (7/7'). To a solution of **6/6'** (200 mg, approx. 1 mmol, 1.0 equiv) and 2,2-dimethoxy propane (5 mL) in acetone (5 mL) was added (±)-camphorsulfonic acid (46 mg, 0.2 mmol, 0.2 equiv). The reaction was stirred at room temperature without rigorous exclusion of air and moisture until TLC analysis indicated complete consumption of **6/6'**. The reaction was quenched with 5% Et_3N/hexanes (2mL) and concentrated *in vacuo*. The residue was purified by flash column chromatography using 15% Et_2O/hexanes as eluent to afford 204 mg (0.84 mmol, 84% in two steps from the three-component coupling) of **7** as a clear oil. Attempted diastereomer separation at this stage was also unsuccessful. Analytical data for **7**: **IR** (thin film, cm^{-1}): 2965, 2878, 1737, 1638, 1381, 1261, 1218, 1119, 1039, 929, 891; **^{1}H NMR** (600 MHz, $CDCl_3$): *major diastereomer*: δ 6.04 (dd, *J* = 16.8, 13.2 Hz, 1H), 5.54 (d, *J* = 16.8 Hz, 1H), 5.33 (d, *J* = 13.2 Hz, 1H), 4.13 (d, *J* = 7.8 Hz, 1H), 3.77 (s, 3H), 1.72-1.60 (m, 2H), 1.54 (s, 3H), 1.43 (s, 3H), 1.15-1.10 (m, 1H), 0.98 (d, *J* = 6.6 Hz, 3H), 0.89 (t, *J* = 7.2 Hz, 3H); *resolved signals for minor diastereomer*: δ 6.08 (dd, *J* = 17.4, 10.8 Hz, 1H), 5.34 (d, *J* = 10.8 Hz, 1H), 4.09 (d, *J* = 9.6 Hz, 1H), 1.46 (s, 3H), 1.28-1.20 (m, 1H); **^{13}C NMR** (150 MHz, $CDCl_3$): *both diastereomers*: δ 172.97, 172.93, 133.8, 133.5, 117.3, 117.1, 109.2, 86.0, 85.5, 85.2, 85.0, 52.7, 35.0, 34.5, 27.24, 27.20, 26.1, 25.9, 25.1, 25.0, 15.6, 14.8, 11.1, 10.2 (two coincident resonances, likely 109.2 and 52.7); **TLC** (20% EtOAc/hexanes), R_f 0.55 (CAM only); **LRMS** (ESI): Calcd. for $C_{13}H_{22}O_4$+Na: 265.14, Found: 265.14

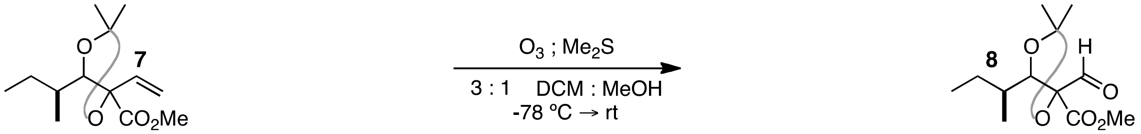

Methyl 5-((*S*)-*sec*-butyl)-4-formyl-2,2-dimethyl-1,3-dioxolane-4-carboxylate (8/8'). A solution of **(7/7')** (41 mg, 0.169 mmol, 1.0 equiv) in 4 mL DCM:MeOH (3:1) was cooled to −78 ºC and sparged with N_2 for 5 min. Ozone was subsequently passed through the solution until the characteristic blue color of ozone saturation was achieved (~10 min). TLC analysis indicated complete consumption of **(7/7')**, and the solution was sparged with N_2 until colorless again. Dimethyl sulfide (0.06 mL, 53 mg, 5.0 equiv) was added, and the reaction mixture was allowed to warm to room temperature and stirred overnight (12 h). TLC analysis to ensure the absence of peroxides was performed, and the reaction mixture was poured into H_2O (50 mL). The layers were separated, the aqueous layer was extracted with 1:1 Et_2O/pentanes (3 × 10 mL), and the combined organic extracts were washed with H_2O (2 × 20 mL) and brine (20 mL), and dried over $MgSO_4$. Filtration and careful concentration *in vacuo* afforded the crude aldehyde which was purified on a silica column that had been packed with 5% Et_3N/hexanes and flushed with

pentanes. Elution with 10% Et_2O/pentanes afforded 36 mg (0.147 mmol, 87% yield) of the desired product **(8/8')** as a clear oil. Analytical data for **(8/8')**: **IR** (thin film, cm^{-1}): 2967, 2938, 2879, 1754, 1737, 1637, 1459, 1438, 1384, 1261, 1221, 1095, 1071, 881; **^{1}H NMR** (600 MHz, $CDCl_3$): *major diastereomer*: δ 9.69 (s, 1H), 4.39 (*d, J* = 7.8 Hz, 1H), 3.83 (s, 3H), 1.78-1.70 (m, 1H), 1.66 (s, 3H), 1.63-1.55 (m, 1H), 1.43 (s, 3H), 1.25-1.15 (m, 1H), 0.97 (d, *J* = 6.6 Hz, 3H), 0.91 (t, *J* = 7.2 Hz, 3H); *resolved signals for minor diastereomer*: δ 9.68 (s, 1H), 4.33 (d, *J* = 9.6 Hz, 1H), 1.70-1.66 (m, 1H); **^{13}C NMR** (150 MHz, $CDCl_3$): *both diastereomers*: δ 196.8, 196.5, 111.5, 111.4, 87.8, 87.3, 86.5, 86.3, 53.2, 53.1, 34.2, 33.9, 30.3, 27.02, 26.99, 26.4, 25.9, 25.0, 15.8, 14.7, 11.1, 10.2 (two coincident resonances); **TLC** (10% EtOAc/hexanes, pretreated plate), R_f 0.19 (CAM only); **LRMS** (ESI): Calcd. for $C_{12}H_{20}O_5$+Na: 267.12, Found: 267.12

Preparation of sulfones for and execution of modified Julia olefination:

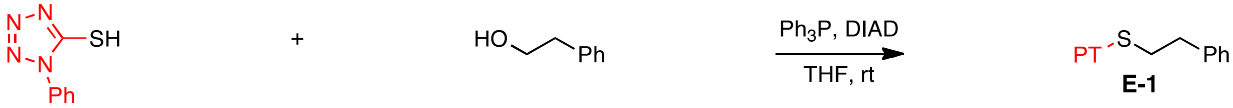

5-(phenethylthio)-1-phenyl-1*H*-tetrazole (E-1). This procedure can be taken as representative for sulfide formation via Mitsunobu reaction with this thiol (*vide infra*). A related procedure for the preparation of **E-1** has been reported previously [18]. To a solution of triphenylphosphine (1.062 g, 4.05 mmol, 1.53 equiv), 1-phenyl-1*H*-tetrazole-5-thiol (0.943 g, 5.29 mmol, 2 equiv), and 2-phenylethanol (0.32 mL, 0.323 g, 2.65 mmol, 1.0 equiv) in THF (25 mL) at 0 ºC was added diisopropylazodicarboxylate (1 mL, 0.974 g, 4.815 mmol, 1.82 equiv), which caused the solution to turn bright yellow. The reaction mixture was allowed to warm to room temperature and stirred overnight (16 h) until it was poured into a solution of 80 mL brine and 20 mL H_2O. The layers were shaken and separated, and the aqueous layer was extracted with Et_2O (3 × 25 mL). The combined extracts were washed with H_2O (20 mL), brine (20 mL), dried over $MgSO_4$, and concentrated *in vacuo* to a sticky yellow semisolid. Purification was effected using a dry-loaded silica gel column, eluting with 10% EtOAc/hexanes to afford 700 mg (2.6 mmol, 98% yield) of the desired product as a thick oil. Characterization data for this compound were consistent with those previously reported [18]: **^{1}H NMR** (300 MHz, $CDCl_3$): δ 7.56 (br s, 4H), 7.35-7.25 (m, 6H), 3.64 (d, *J* = 7.2 Hz, 2 H), 3.16 (d, *J* = 7.2 Hz, 2 H); **TLC** (20% EtOAc/hexanes) R_f 0.45 (UV/CAM).

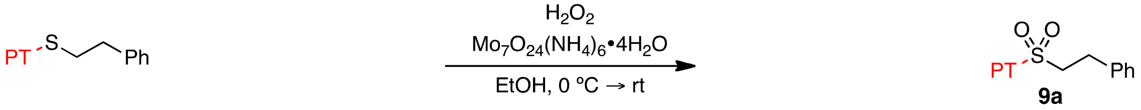

5-(phenethylsulfonyl)-1-phenyl-1*H*-tetrazole (9a). This procedure is representative of the oxidation of a sulfide to a sulfone, and was quite general for a variety of substrates. A related procedure for the preparation of **9a** has been reported previously [18]. A solution of the sulfide (700 mg, 2.6 mmol, 1.0 equiv) in EtOH (25 mL) was cooled to 0 ºC while a solution of ammonium molybdate tetrahydrate in 30% aq H_2O_2 (240 mg/mL) was prepared. This bright yellow solution (3 mL) was added to the solution of the substrate in EtOH, and allowed to warm to room temperature with stirring overnight open to air. The reaction was poured into 100 mL H_2O and extracted with EtOAc (3 × 20 mL). The combined organic extracts were washed with H_2O (2 × 20 mL) and brine (20 mL), dried over $MgSO_4$, and concentrated *in vacuo* to a solid which required no further purification (735 mg, 90% yield). Analytical data for **9a** were consistent with those previously reported [18]: **IR** (thin film, cm^{-1}): 3065, 3030, 2920, 1595, 1498, 1456, 1342, 1238, 1154, 1076, 916, 764; **^{1}H NMR** (400 MHz, $CDCl_3$): δ 7.71 (m, 2H),

7.66-7.60 (m, 3H), 7.36 (m, 2H), 7.30-7.26 (m, 3H), 4.01 (m, 2H), 3.28 (m, 2H); **^{13}C NMR** (150 MHz, $CDCl_3$): 153.3, 136.3, 132.9, 131.5, 129.7, 129.0, 128.5, 127.4, 125.0, 57.2, 28.4; **TLC** (20% EtOAc/hexanes), R_f 0.22 (UV; faint in CAM); **LRMS** (ESI): Calcd. for $C_{15}H_{14}N_4O_2S$+Na: 337.07, Found: 337.07; Calcd. for $C_{15}H_{14}N_4O_2S$+Cs: 446.99, Found: 446.99

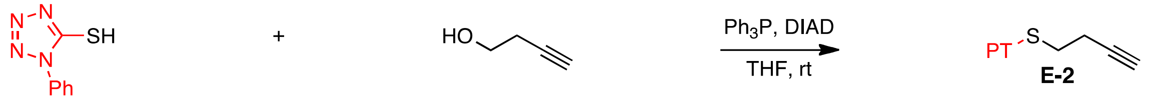

5-(but-3-yn-1-ylthio)-1-phenyl-1*H*-tetrazole (E-2). This reaction was performed analogously to the Mitsunobu procedure detailed above. Related procedures for the preparation of **E-2** have been reported previously [19,20]. The Mitsunobu reaction was conducted using triphenylphosphine (2.24 g, 8.54 mmol, 1.53 equiv), 1-phenyl-1*H*-tetrazole-5-thiol (2 g, 11.2 mmol, 2 equiv), and 3-butyn-1-ol (0.42 mL, 0.391 g, 5.58 mmol, 1.0 equiv), THF (50 mL), and diisopropylazodicarboxylate (2 mL, 2.054 g, 10.2 mmol, 1.82 equiv) Partial purification was achieved using flash column chromatography, eluting with 20% EtOAc/hexanes. The isolated material was contaminated by the starting thiol, which could be conveniently removed by basic extraction to afford the desired product: 1.2 g (5.2 mmol, 93% yield) as a thick semisolid. Analytical data for **E-2** were consistent with those reported [20]: **IR** (thin film, cm^{-1}): 3293, 3064, 2934, 2118, 1597, 1499, 1462, 1414, 1388, 1281, 1243, 1091, 1015, 762; **^{1}H NMR** (600 MHz, $CDCl_3$): δ 7.58-7.50 (m, 5H), 3.53 (t, J = 7.2 Hz, 2H), 2.80 (dt, J = 6.6, 2.4 Hz, 2H), 2.05 (t, J = 2.4 Hz, 1H); **^{13}C NMR** (150 MHz, $CDCl_3$): 153.6, 133.5, 130.2, 129.8, 123.7, 81.1, 70.6, 32.0, 19.2; **TLC** (20% EtOAc/hexanes), R_f 0.25 (UV/CAM); **LRMS** (ESI): Calcd. for $C_{11}H_{11}N_4S$+Na: 253.05, Found: 253.06; Calcd. for $C_{11}H_{10}N_4S$+Cs: 362.97, Found: 362.97

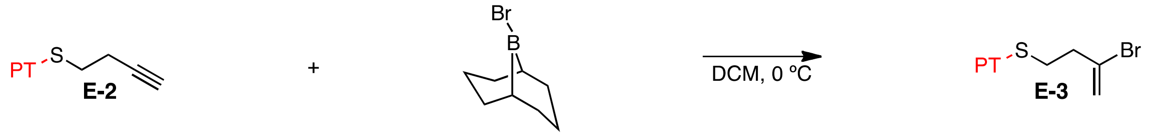

5-((3-bromobut-3-en-1-yl)thio)-1-phenyl-1*H*-tetrazole (E-3). This procedure was performed analogously to a literature procedure [21]. A cloudy suspension of sulfide **E-2** (1.2 g, 5.2 mmol, 1.0 equiv) in dry DCM (50 mL) was cooled to 0 ºC under N_2. A solution of 9-Br-9-BBN (1 M in DCM, 6.25 mL, 6.25 mmol, 1.2 equiv) was added, and the mixture gradually cleared noticeably and became a pale yellow solution as the mixture was stirred for 3 h at 0 ºC. Glacial acetic acid (~4 mL) was added neat, dropwise to the reaction mixture, followed 1 h later by 3 M NaOH (25 mL) and 30% aq H_2O_2 (10 mL). The biphasic mixture was stirred vigorously for 30 min, and the layers were separated. The aqueous layer was extracted with DCM (3 × 10 mL), and the combined organic extracts were washed with brine (20 mL) and dried over Na_2SO_4. Concentration *in vacuo* and analysis of the crude mixture revealed that only approx. 33% conversion had occurred. Purification by flash column chromatography with 15% EtOAc/hexanes as eluent afforded 336 mg (1.08 mmol, 59% yield brsm) of the product as a clear oil. Analytical data for **E-3**: **IR** (thin film, cm^{-1}): 3101, 3064, 2927, 1628, 1597, 1499, 1412, 1387, 1244, 1188, 1090, 1015, 896, 761; **^{1}H NMR** (600 MHz, $CDCl_3$): δ 7.57-7.50 (m, 5H), 5.67 (d, J = 1.8 Hz, 1H), 5.51 (d, J = 1.8 Hz, 1H), 3.59 (d, J = 7.2 Hz, 2H), 3.00 (d, J = 7.2 Hz, 2H); **^{13}C NMR** (150 MHz, $CDCl_3$): 153.7, 133.5, 130.4, 130.2, 129.8, 123.7, 119.5, 40.4, 31.4; **TLC** (20% EtOAc/hexanes), R_f 0.32 (UV/CAM); **LRMS** (ESI): Calcd. for $C_{11}H_{11}BrN_4S$+Na: 332.98, Found: 332.98; Calcd. for $C_{11}H_{11}BrN_4S$+Cs: 442.89, Found: 442.90

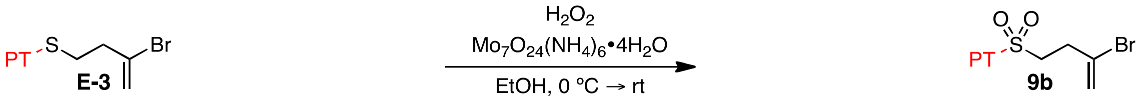

5-((3-bromobut-3-en-1-yl)sulfonyl)-1-phenyl-1*H*-tetrazole (9b). This oxidation was performed analogously to the oxidation procedure reported above for the preparation of **9a**. Here, **E-3** (335 mg, 1.075 mmol, 1 equiv), and an ammonium molybdate/aq H_2O_2 solution (240 mg/mL; 1.2 mL) were employed. Purification was effected using flash column chromatography using 20% EtOAc/hexanes as eluent to afford 330 mg (0.962 mmol, 89% yield) of the product **9b** as a white solid. Analytical data for **9b**: **IR** (thin film, cm^{-1}): 2988, 2923, 1631, 1497, 1348, 1151, 903, 763, 688; ^{1}**H NMR** (600 MHz, $CDCl_3$): δ 7.69 (d, *J* = 7.2 Hz, 2H), 7.65-7.60 (m, 3H), 5.81 (s, 1H), 5.56 (s, 1H), 3.99 (m, 2H), 3.11 (m, 2H); ^{13}**C NMR** (150 MHz, $CDCl_3$): 153.2, 132.8, 131.6, 129.8, 127.5, 125.0, 120.4, 54.6, 34.4; **TLC** (20% EtOAc/hexanes), R_f 0.24 (UV/CAM); **LRMS** (ESI): Calcd. for $C_{11}H_{11}BrN_4O_2S$+Na: 364.97, Found: 364.97

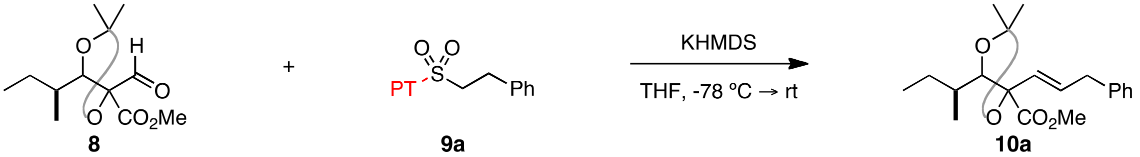

Methyl 5-((*S*)-*sec*-butyl)-2,2-dimethyl-4-((*E*)-3-phenylprop-1-en-1-yl)-1,3-dioxolane-4-carboxylate (10/10a'). To a stirred solution of **9a** (33 mg, 0.104 mmol, 1.1 equiv) in dry THF (1 mL) under N_2 and cooled to −78 °C was added a solution of potassium hexamethyldisilazide (0.5 M in toluene, 0.28 mL, 0.141 mmol, 1.5 equiv), and the clear solution became yellow. After 15 min at this temperature, a solution of aldehyde **8** (23 mg, 0.094 mmol, 1.0 equiv) in THF (1 mL) was added via syringe, followed by a THF rinse to ensure complete transfer (0.5 mL). The reaction was allowed to warm slowly to room temperature overnight (12 h), by which point the yellow color had faded and the reaction had become cloudy due to precipitate formation. TLC analysis indicated complete consumption of the aldehyde **8**, and the reaction was quenched with sat. aq NH_4Cl (5 mL). The mixture was diluted with Et_2O (5 mL) and H_2O (30 mL), the layers were separated, and the aqueous layer was extracted with Et_2O (3 × 5 mL). The combined organic extracts were washed with H_2O and brine, dried over $MgSO_4$, and concentrated *in vacuo*. The residue was purified by flash column chromatography using 5% EtOAc/hexanes to afford 29 mg (0.087 mmol, 93% yield) of **10a/10a'** as a clear oil. Analytical data for **10a/10a'**: ^{1}**H NMR** (300 MHz, $CDCl_3$): *major diastereomer*: δ 7.30-7.26 (m, 2H), 7.22- 7.15 (m, 3H), 6.12-6.02 (m, 1H), 5.63 (d, *J* = 15.3 Hz, 1H), 4.11 (d, *J* = 5.1 Hz, 1H), 3.77 (s, 3H), 3.48-3.40 (m, 2H), 1.75-1.60 (m, 1H), 1.50 (s, 3H), 1.42 (s, 3H), 1.30-1.00 (m, 2H), 0.97 (d, *J* = 6.3 Hz, 3H), 0.86 (t, *J* = 7.2 Hz, 3H); *resolved signals for minor diastereomer*: δ 5.72 (d, *J* = 15.3 Hz, 1H), 4.09 (d, *J* = 5.1 Hz, 1H), 1.43 (s, 3H); **TLC** (20% EtOAc/hexanes) R_f 0.51 (CAM).

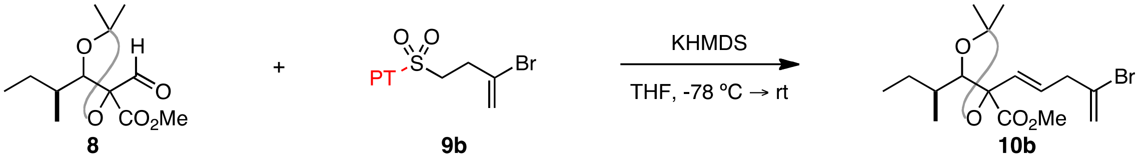

Methyl 4-((*E*)-4-bromopenta-1,4-dien-1-yl)-5-((*S*)-*sec*-butyl)-2,2-dimethyl-1,3-dioxolane-4-carboxylate (10b/10b'). The reaction was performed according to the Julia olefination procedure above, using **9b** (42 mg, 0.123 mmol, 1.0 equiv), KHMDS (0.5M in toluene, 0.37 mL, 0.184 mmol, 1.5 equiv), and aldehyde **8** (30 mg, 0.123 mmol, 1.0 equiv) and the same volumes of THF. Purification was effected by flash column chromatography using 10% EtOAc/hexanes as

eluent, to afford 35 mg (0.097 mmol, 79% yield) as a clear oil. Analytical data for **10b**/**10b'**: **IR** (thin film, cm^{-1}): 2964, 2936, 2877, 1738, 1630, 1460, 1435, 1381, 1371, 1259, 1216, 1117, 1065, 1033, 976, 893; **^{1}H NMR** (600 MHz, $CDCl_3$): *major diastereomer*: δ 5.96-5.88 (m, 1H), 5.78 (d, *J* = 15.6 Hz, 1H), 5.6 (s, 1H), 5.45 (s, 1H), 4.11 (d, *J* = 7.2 Hz, 1H), 3.77 (s, 3H), 3.23 (m, 2H), 1.72-1.65 (m, 2H), 1.53 (s, 3H), 1.44 (s, 3H), 1.43 (m, 1H), 0.87 (m, 6H); *resolved signals for minor diastereomer*: δ 5.82 (d, *J* = 15.6 Hz, 1H), 4.07 (d, *J* = 9.6 Hz, 1H), 1.445 (s, 3H), 1.25-1.15 (m, 1H), 1.15-1.05 (m, 1H), 0.97 (d, *J* = 6.6 Hz); **^{13}C NMR** (150 MHz, $CDCl_3$): *both diastereomers*: δ 172.92, 172.89, 131.7, 131.5, 129.3, 129.2, 128.2, 128.1, 117.42, 117.38, 109.2, 86.1, 85.6, 84.8, 84.6, 52.7, 44.1, 35.0, 34.5, 27.29, 27.26, 26.1, 25.9, 25.0, 24.9, 15.6, 14.9, 11.1, 10.3 (three coincident resonances: likely 109.2, 52.7, and 44.1); **TLC** (10% EtOAc/hexanes, pretreated plate), R_f 0.38 (CAM only); **LRMS** (ESI): Calcd. for $C_{16}H_{25}BrO_4$+Na: 383.08, Found: 383.08; Calcd. for $C_{16}H_{25}BrO_4$+Cs: 493.00, Found: 493.00.

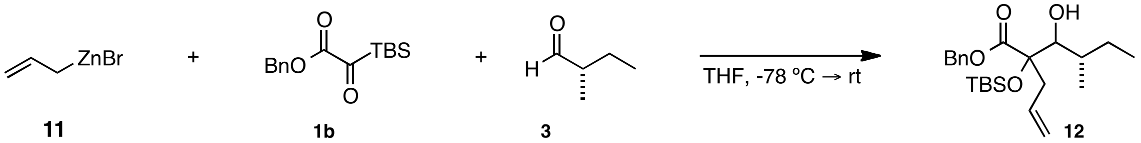

(4*S*)-benzyl 2-allyl-2-(*tert*-butyldimethylsilyloxy)-3-hydroxy-4-methylhexanoate (12). Allylzinc bromide **11** was prepared analogously to a reported procedure [22]. The zinc dust could be activated either with 1,2-dibromoethane/TMSCl or using Br_2. A typical preparation using the latter method will be described: a dry flask with stir bar was charged with Zn dust (~330 mg, approx. 5 mmol, 1.2 equiv) in the glovebox, sealed with a septum, and brought out of the glovebox. THF (4 mL) was added, and the vigorously stirred suspension was cooled to 0 ºC for 5 min, and Br_2 (0.03 mL, 0.5 mmol, 0.10 equiv relative to Zn^0) was added dropwise. The Br_2 color quickly dissipated, and after an additional 5 min allyl bromide (0.35 mL, 4 mmol, 1.0 equiv) was added dropwise. The suspension was stirred at 0 ºC for 1 h, at which point stirring was stopped and the excess of Zn dust was allowed to settle. The solution of allylzinc bromide thus prepared was assumed to be 1.0 M.

The appropriate amount of such a solution (in this case: 0.30 mL, 0.30 mmol, 1.5 equiv) was added to a stirred solution of Bn/TBS silyl glyoxylate **1b** (56 mg, 0.20 mmol, 1.0 equiv) and (*S*)-2-methylbutanal **3** (0.30 mmol, 1.5 equiv) in 3 mL THF, which had been cooled to −78 ºC for ≥15 min. The bright yellow gold color due to **1b** dissipated, and 15 min later the reaction was warmed to rt for 3 h and quenched with sat. NH_4Cl (5 mL). The mixture was diluted with H_2O (30 mL), and the layers were separated. The aqueous layer was extracted with Et_2O (3 × 10 mL), and the combined extracts were washed with brine and dried over $MgSO_4$. The mixture was filtered and concentrated *in vacuo*, and the residue was purified by flash column chromatography eluting with 2.5% Et_2O/hexanes. "Diastereomers A" eluted first, followed by "diastereomers B" (typically with mixed fractions in between), and the combined yield in this run was 48 mg (0.118 mmol, 60% yield) of approx. 1:2.4 "diastereomers A":"diastereomers B". **^{1}H NMR** for **12**, "diastereomers A": (400 MHz, $CDCl_3$): *major diastereomer*: δ 5.76-5.60 (m, 1H), 5.70-5.60 (m, 2H), 5.60-4.48 (m, 2H), 3.67 (dd, *J* = 10.8, 2.4 Hz, 1H), 2.72-2.62 (m, 1H), 2.62-2.52 (m, 1H), 2.0 (d, *J* = 10.8, 1H), 1.45-1.35 (m, 2H), 1.20-1.08 (m, 1H), 0.88 (s, 9H), 0.84 (d, *J* = 6.8 Hz, 3H), 0.75 (t, *J* = 7.2 Hz, 3H), 0.19 (s, 3H), 0.15 (s, 3H); *resolved signals for minor diastereomer*: δ 3.59 (dd, *J* = 10.4, 6.6 Hz, 1H), 1.80 (d, *J* = 10.4 Hz, 1H), 1.70-1.60 (m, 1H), 1.55-1.45 (m, 1H), 0.95-0.88 (m, 1H), 0.88 (s, 9H), 0.83-0.77 (m, 6H), 0.17 (s, 3H), 0.16 (s, 3H); **TLC** (5% EtOAc/hexanes) R_f 0.27 (CAM).

Analytical data for **12**, "diastereomers B": **IR** (thin film, cm^{-1}): 3464, 3078, 2957, 2930, 2857, 1750, 1641, 1462, 1388, 1254, 1215, 1139, 919, 837, 778; **^{1}H NMR** (600 MHz, $CDCl_3$): *major diastereomer*: δ 7.5 (br s, 5H), 5.78-5.75 (m, 1H), 5.15 (d, *J* = 12 Hz, 1H), 5.12 (d, *J* =12 Hz, 1 H), 5.06 (app d, *J* = 12 Hz, 2H), 3.66 (dd, *J* = 10.8, 2.4 Hz, 1H), 2.66 (dt, *J* = 14.4, 7.2 Hz, 1H), 2.53 (dt, *J* = 14.4, 7.2 Hz, 1H), 2.36 (d, *J* = 10.8 Hz, 1H), 1.70-1.60 (m, 1H), 1.45-1.39 (m, 1H), 1.30-1.24 (m, 1H), 0.87 (t, *J* = 7.2 Hz, 3H), 0.87 (s, 9H), 0.77 (d, *J* = 6.6 Hz, 3H), 0.15 (s, 3H), 0.12 (s, 3H); *resolved signals for minor diastereomer*: δ 3.56 (dd, *J* = 9.6, 3.6 Hz, 1H), 2.30 (d, *J* = 9.6 Hz, 1H), 0.93 (d, *J* = 7.2 Hz, 3H), 0.83 (t, *J* = 7.2 Hz); **^{13}C NMR** (150 MHz, $CDCl_3$): *both diastereomers* δ 174.0, 173.8, 134.94, 134.90, 132.8, 132.6, 128.9, 128.8, 128.61, 128.59, 128.57, 119.0, 118.9, 83.0, 82.7, 80.3, 78.1, 67.34, 67.27, 42.3, 41.9, 35.1, 34.4, 28.7, 26.2, 26.1, 23.0, 19.0, 17.6, 12.9, 11.9, 11.6, −2.0, −2.2, −2.46, −2.54 (two coincident resonances); **TLC** (5% EtOAc/hexanes), R_f 0.18 (CAM); **LRMS** (ESI): Calcd. for $C_{23}H_{38}O_4$+Na: 429.24, Found: 429.25.

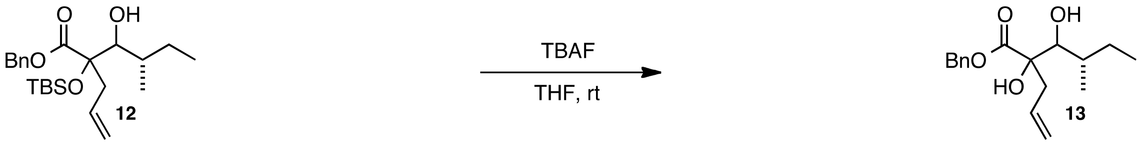

(4*S*)-benzyl 2-allyl-2,3-dihydroxy-4-methylhexanoate (13). "Diastereomers A" of three-component coupling product **12** (180 mg, 0.443 mmol, 1.0 equiv) were dissolved in 2 mL THF, and then TBAF (1 M in THF, 2.2 mL, 2.2 mmol, 5 equiv) was added and the reaction mixture was stirred at room temperature for 1.5 h. The mixture was poured into H_2O (50 mL), and the layers were shaken and separated. The aqueous layer was extracted with Et_2O (3 × 10 mL), and the combined organic extracts were washed with H_2O (2 × 20 mL) and brine (20 mL), and then dried over $MgSO_4$. Purification of the residue by flash column chromatography using 15% EtOAc/hexanes afforded 100 mg of the product as a clear oil (0.342 mmol, 77% yield). Analytical data for **13**: **^{1}H NMR** (400 MHz, $CDCl_3$): *major diastereomer*: δ 7. 37 (br s, 5H), 5.75-5.48 (m, 1H), 5.23 (d, *J* = 12 Hz, 1H), 5.17 (d, *J* = 12 Hz, 1H), 5.05-4.98 (m, 2H), 3.83 (dd, *J* = 10.8, 1.6 Hz, 1H), 3.49 (s, 1H), 2.49-2.40 (m, 2H), 2.13 (d, *J* = 10.8 Hz, 1H), 1.78-1.55 (m, 1H), 1.50-1.39 (m, 1H), 1.37-1.28 (m, 1H), 0.95-0.85 (m, 6H); *resolved signals for the minor diastereomer*: δ 3.71 (dd, *J* = 11.2, 2.8 Hz, 1H), 3.49 (s, 1H), 2.12 (d, *J* = 11.2 Hz, 1H), 1.01 (d, *J* = 7.2 Hz, 3H); **TLC** (15% EtOAc/hexanes), R_f 0.44 (CAM)

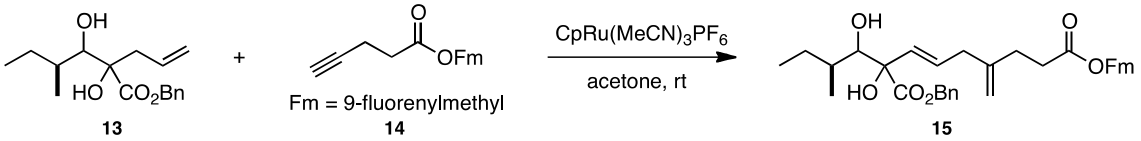

(*E*)-9-(9-fluorenylmethyl) 1-benzyl 2-hydroxy-2-((2*S*)-1-hydroxy-2-methylbutyl)-6-methylenenon-3-enedioate (15). A vial was charged with cyclopentadienylruthenium (tris-acetonitrile) hexafluorophosphate (3 mg, 0.0075 mmol, 0.10 equiv) and a stir bar. A solution of **13** (22 mg, 0.075 mmol, 1.0 equiv) and (9-fluorenylmethyl) 4-pentynoate **14** (21 mg, 0.075 mmol, 1.0 equiv) in 0.80 mL acetone was added and the reaction mixture was stirred overnight (~18 h), and then poured into H_2O (10 mL). The cloudy mixture was extracted with Et_2O (3 × 5 mL), and the combined organic extracts were washed with brine (10 mL) and dried over $MgSO_4$. Purification of the residue by flash column chromatography using 15% EtOAc/hexanes as eluent afforded 22 mg of the product as a clear oil (0.0387 mmol, 52% yield). Analytical data for **15**: **^{1}H NMR** (400 MHz, $CDCl_3$): *major diastereomer*: δ 7.77 (d, *J* = 7.6 Hz, 2H), 7.59 (d, *J* = 7.6 Hz, 2H), 7.45-7.28 (m, 9H), 6.05-5.95 (m, 1H), 5.58 (d, *J* = 15.2 Hz, 1H), 5.28 (d, *J* = 9 Hz, 1H),

5.22 (d, J = 9 Hz, 1H), 4.75 (s, 1H), 4.73 (s, 1H), 4.39 (d, J = 7.2 Hz, 2H), 4.20 (t, J = 7.2 Hz, 1H), 3.98 (m, 1H), 3.62 (s, 1H), 2.76 (m, 2H), 2.51 (t, J = 7.2 Hz, 2H), 2.30 (t, J = 7.2 Hz, 2H), 2.10 (d, J = 10.2 Hz, 1H), 1.78-1.55 (m, 1H), 1.50-1.39 (m, 1H), 1.37-1.28 (m, 1H), 0.95-0.85 (m, 6H); *resolved signals for the minor diastereomer*: δ 7.53 (d, J = 7.6 Hz, 2H), 3.85 (m, 1H), 3.66 (s, 1H), 0.95 (d, J = 6.8 Hz, 3H) **TLC** (20% EtOAc/hexanes) R_f 0.29 (CAM).

Synthesis of dithiane aldehyde 16ba:

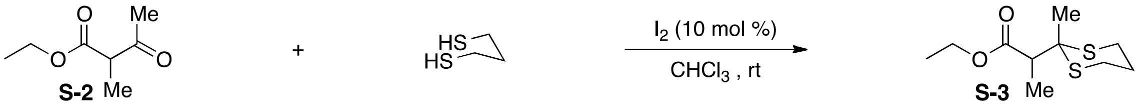

Ethyl 2-(2-methyl-1,3-dithian-2-yl)propanoate (S-3). This procedure was conducted analogously to a literature procedure [23]. To a solution of ethyl 2-methylacetoacetate **(S-2)** [24] (5 g, 34.68 mmol, 1 equiv) and 1,3-propanedithiol (4.2 mL, 4.5 g, 41.6 mmol, 1.2 equiv) in chloroform (125 mL) was added iodine (880 mg, 3.47 mmol, 0.1 equiv), and the resultant deep red solution was stirred at room temperature overnight without the need for an N_2 atmosphere (12 h). The reaction was worked up by pouring into 10% aq (w/w) KOH (75 mL) and sat. aq $Na_2S_2O_3$ (75 mL) and shaking vigorously until the I_2 color had dissipated. The layers were separated, and the aqueous layer was extracted with additional chloroform (2 × 10 mL). The combined organic extracts were washed with H_2O (20 mL) and brine (20 mL), dried over Na_2SO_4 and concentrated *in vacuo*. The crude yellow oil obtained (8 g, 34.1 mmol, 98% crude yield) required no further purification. Analytical data for **S-3**: **IR** (thin film, cm^{-1}): 2977, 2935, 2904, 2830, 1731, 1641, 1446, 1423, 1372, 1335, 1254, 1184, 1111, 1070, 1043, 1020, 906, 866; **^{1}H NMR** (600 MHz, $CDCl_3$): δ 4.22-4.08 (m, 2H), 3.35 (q, J = 7.2 Hz, 1H), 3.20-3.10 (m, 1H), 3.0-2.90 (m, 1H), 2.68-2.60 (m, 2H), 2.10-2.03 (m, 1H), 1.80-1.70 (m, 1H), 1.59 (s, 3H), 1.29 (d, J = 7.2 Hz, 3H), 1.26 (t, J = 7.2 Hz, 3H); **^{13}C NMR** (150 MHz, $CDCl_3$): δ 173.2, 60.4, 49.2, 45.6, 26.5, 26.4, 24.5, 23.1, 14.1, 13.5; **TLC** (20 % EtOAc/hexanes) R_f 0.47 (UV/CAM); **LRMS** (ESI): Calcd. for $C_{10}H_{18}O_2S_2$+Na: 257.06; Found: 257.09.

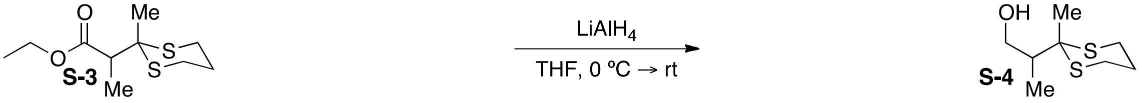

2-(2-Methyl-1,3-dithian-2-yl)propan-1-ol (S-4). To a solution of dithiane ester **S-3** (5.7 g, 24 mmol, 1 equiv) in dry THF (100 mL) under N_2 at 0 °C was added a solution of lithium aluminum hydride (1 M in THF, 18 mL, 18 mmol, 0.75 equiv). The reaction mixture was allowed to warm to room temperature, and upon completion, the solution was cooled again to 0 °C. A sat. aq solution of Rochelle's salt (Na/K tartrate) was added carefully until the mixture no longer effervesced. Once the reaction mixture was thus quenched, additional Rochelle's salt solution (~200 mL) was added and the mixture was stirred vigorously until the two layers cleanly separated when stirring was stopped (1–2 h). The aqueous layer was extracted with Et_2O (3 × 50 mL), and the combined organic extracts were washed with H_2O (50 mL) and brine (50 mL), dried over $MgSO_4$, and concentrated *in vacuo*. The material thus obtained required no further purification, and its spectral properties matched those reported in the literature [25].

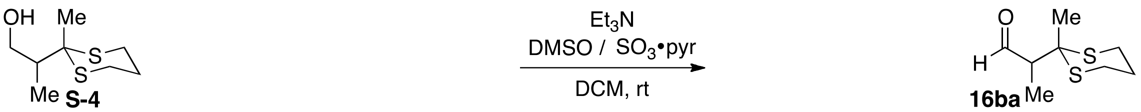

2-(2-methyl-1,3-dithian-2-yl)propanal (16ba). This reaction has been reported in a footnote, but no experimental details were given in the text or supporting information [26]. To a solution

of alcohol **S-4** (1 g, 5.2 mmol, 1 equiv) in DCM (20 mL) were added dimethylsulfoxide (3.6 mL, 3.94 g, 50.4 mmol, 9.7 equiv), triethylamine (7.25 mL, 5.26 g, 52 mmol, 10.4 equiv), and the sulfur trioxide·pyridine complex (4.3 g, 27 mmol, 5.2 equiv). The reaction mixture was stirred 1 h at room temperature, and then the mixture was poured into H_2O (150 mL). The layers were shaken and separated, and the aqueous layer was extracted with DCM (3 × 20 mL). The combined organic extracts were washed with H_2O (2 × 30 mL) and brine (30 mL), dried over Na_2SO_4, and concentrated in vacuo. The residue was purified by flash column chromatography using 20% Et_2O/hexanes to afford 660 mg (66% yield) of the product **32cb** as a colorless oil. Analytical data for **32cb**: IR (thin film, cm^{-1}): 2973, 2934, 2907, 2830, 2731, 1715, 1446, 1422, 1375, 1277, 1239, 1135, 1110, 1077, 906; **^{1}H NMR** (600 MHz, $CDCl_3$): δ 9.81 (d, *J* = 3.6 Hz, 1H), 3.03-2.98 (m, 1H), 2.97-2.90 (m, 1H), 2.89-2.85 (m, 1H), 2.80-2.70 (m, 2H), 2.10-2.00 (m, 1H), 1.97-1.89 (m, 1H), 1.59 (s, 3H), 1.20 (d, *J* = 7.2 Hz, 3H); **^{13}C NMR** (150 MHz, $CDCl_3$): δ 201.3, 51.1 48.5, 26.3, 25.7, 24.4, 24.3, 10.5; **TLC** (10% EtOAc/hexanes) R_f 0.20 (UV/CAM); **LRMS** (ESI): Calcd. for $C_8H_{14}OS_2$+Na: 213.04, Found: 213.05

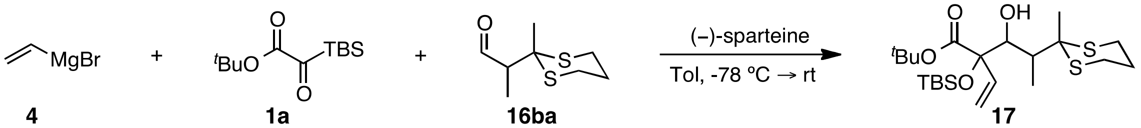

tert-butyl 2-(_tert_-butyldimethylsilyloxy)-3-hydroxy-4-(2-methyl-1,3-dithian-2-yl)-2-vinylpentanoate (17). The title compound was prepared according to General Procedure A using vinylmagnesium bromide (0.7 M in THF, 4.3 mL, 3 mmol), *t*-Bu/TBS silyl glyoxylate **16a** (489 mg, 2 mmol, 1.0 equiv), dithiane aldehyde **32cb** (571 mg, 3 mmol, 1.5 equiv), and (−)-sparteine (703 mg, 3 mmol, 1.5 equiv). The total volume of the (−)-sparteine/Grignard solution was 20 mL dry toluene, and the silyl glyoxylate/aldehyde solution was 40 mL. Analysis of the crude ^{1}H NMR indicated formation of a single diastereomer, which was purified and isolated by flash column chromatography using 2.5% Et_2O/hexanes to afford **35a** 590 mg (1.28 mmol, 64% yield) as a white solid. Analytical data for **35a**: **IR** (thin film, cm^{-1}): 3573, 2930, 2903, 2857, 1746, 1640, 1472, 1393, 1369, 1253, 1157, 995, 917, 842; **^{1}H NMR** (600 MHz, $CDCl_3$): δ 6.00 (dd, *J* = 17.4, 10.8 Hz, 1H), 5.52 (d, *J* = 17.4, 1H), 5.27 (d, *J* = 10.8 Hz, 1H), 4.59 (d, *J* = 10.8 Hz, 1H), 2.85-2.80 (m, 1H), 2.75-2.65 (m, 3H), 2.44 (q, *J* = 7.2 Hz, 1H), 2.29 (d, *J* = 10.8 Hz, 1H), 1.95-1.85 (m, 2 H), 1.54 (s, 3H), 1.50 (s, 9H), 0.25 (s, 3H) 0.12 (s, 3H); **^{13}C NMR** (150 MHz, $CDCl_3$): δ 171.2, 138.1, 116.5, 85.9, 82.3, 74.4, 54.3, 38.5, 28.0, 26.8, 26.4, 26.3, 25.0, 23.9, 19.4, 9.5, −1.9, −2.1 (one coincident resonance); **TLC** (20% EtOAc/hexanes), R_f0.53 (UV/CAM); **LRMS** (ESI): Calcd. for $C_{22}H_{42}O_4S_2Si$+Na: 485.22, Found: 485.21; Calcd. for $C_{22}H_{42}O_4S_2Si$+Cs: 595.13, Found: 595.13

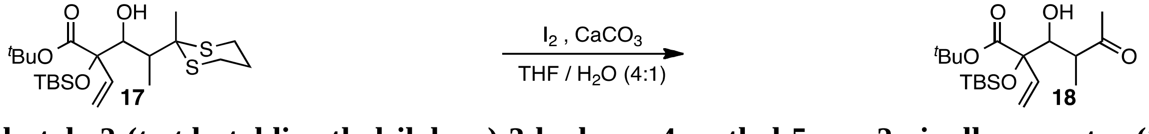

tert-butyl 2-(_tert_-butyldimethylsilyloxy)-3-hydroxy-4-methyl-5-oxo-2-vinylhexanoate (18). Three-component coupling product **17** (149 mg, 0.321 mmol, 1.0 equiv) was dissolved in 4:1 THF:H_2O (5 mL tot) and the mixture was cooled to 0 ºC. Calcium carbonate (32 mg, 0.321 mmol, 1.0 equiv) was added, and 5 min later iodine (244 mg, 0.963 mmol, 3 equiv) was added and the solution became dark red-brown. The reaction was stirred at 0 ºC open to air until judged complete by TLC analysis; this was typically checked by NMR analysis of an aliquot because on more than one occasion the reaction was terminated prematurely due to misleading TLC

appearance. When complete, the reaction was worked up by pouring into half-saturated aq $Na_2S_2O_3$ and shaking vigorously until the I_2 color had dissipated. The layers were separated, and the aqueous layer was extracted with Et_2O (3 × 10 mL). The combined organic extracts were washed with H_2O (20 mL) and brine (20 mL), dried over $MgSO_4$ and concentrated *in vacuo* to afford the ketone **18** (which typically required no further purification as long as the reaction had gone to completion). An analytical sample was purified using flash column chromatography, eluting with 7.5% EtOAc/hexanes to afford 100 mg (0.268 mmol, 83% yield) of the desired product as a white solid. Analytical data for **18**: **IR** (thin film, cm^{-1}): 3485, 2955, 2931, 2856, 1747, 1713, 1460, 1369, 1253, 1161, 1056, 841; ^{1}**H NMR** (600 MHz, $CDCl_3$): δ 5.78 (dd, *J* = 17.4, 10.8 Hz, 1H), 5.45 (d, *J* = 17.4, 1H), 5.15 (d, *J* = 10.8 Hz, 1H), 4.31 (dd, *J* = 10.8, 7.8 Hz, 1H), 2.85 (quint, *J* = 7.2 Hz, 1H), 2.16 (d, *J* = 10.8 Hz, 1H), 2.11 (s, 3H), 1.49 (s, 9H), 1.11 (d, *J* = 7.2 Hz, 3H), 0.95 (s, 9H), 0.22 (s, 3H) 0.16 (s, 3H); ^{13}**C NMR** (150 MHz, $CDCl_3$): δ 211.3, 170.9, 137.3, 116.4, 84.4, 82.7, 47.5, 29.4, 27.9, 26.51, 26.48, 19.4, 13.6, −2.2, −2.6; **TLC** (20% EtOAc/hexanes), R_f 0.45 (UV/CAM); **LRMS** (ESI): Calcd. for $C_{19}H_{36}O_5Si$+Na: 395.22, Found: 395.22; Calcd. for $C_{19}H_{36}O_5Si$+Cs: 505.14, Found: 505.13

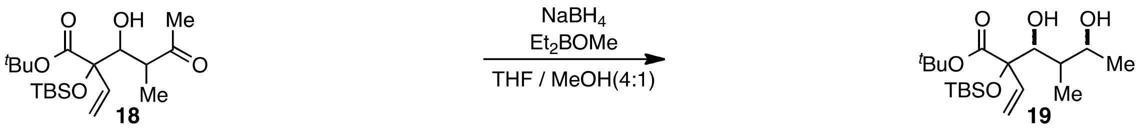

(3*R,5*R**)-*tert*-butyl 2-(*tert*-butyldimethylsilyloxy)-3,5-dihydroxy-4-methyl-2-vinylhexanoate (19).** A solution of **18** in (60 mg, 0.161 mmol) 4:1 THF:MeOH (2 mL) was cooled to −78 ºC, a solution of diethylmethoxyborane (1 M in THF, 0.21 mL, 0.21 mmol, 1.3 equiv) was added, and the solution was stirred for 45 min prior to the addition of sodium borohydride (18 mg, 0.483 mmol, 3 equiv). The reaction was moved to a cryocool, set to −70 ºC, and stirred at this temperature until judged complete by TLC analysis. The reaction was quenched by pouring into MeOH (10 mL) and carefully adding glacial acetic acid to the mixture, which effervesced. The reaction was concentrated *in vacuo*, redissolved in MeOH, and concentrated again (3×). The crude residue was triturated and filtered with Et_2O and concentrated to a clear oil. The residue was purified by flash column chromatography, using 15% EtOAc/hexanes as eluent to afford 50 mg (0.133 mmol, 83% yield) of **19** as a clear colorless oil. Analytical data for **19**: **IR** (thin film, cm^{-1}): 3438, 2974, 2930, 2857, 1747, 1472, 1462, 1370, 1252, 1153, 927, 840, 779; ^{1}**H NMR** (600 MHz, $CDCl_3$): δ 5.94 (dd, *J* = 17.4, 10.8 Hz, 1H), 5.42 (d, *J* = 17.4, 1H), 5.29 (d, *J* = 10.8 Hz, 1H), 4.00 (m, 2H), 3.22 (d, *J* = 10.2 Hz, 1H), 3.04 (br s, 1H), 1.86-1.80 (m, 1H), 1.52 (s, 9H), 1.22 (d, *J* = 6 Hz, 3H), 0.91 (s, 9H), 0.91 (d, *J* = 7.2 Hz, 3H), 0.20 (s, 3H) 0.12 (s, 3H); ^{13}**C NMR** (150 MHz, $CDCl_3$): δ 171.8, 138.0, 117.0, 83.2, 82.5, 81.0, 72.3, 38.8, 28.0, 26.3, 20.3, 19.0, 5.9, −2.2, −2.3; **TLC** (20% EtOAc/hexanes), R_f 0.26 (CAM); **LRMS** (ESI): Calcd. for $C_{19}H_{38}O_5Si$+Na: 397.24, Found: 397.23; Calcd. for $C_{19}H_{38}O_5Si$+Cs: 507.15, Found: 507.15

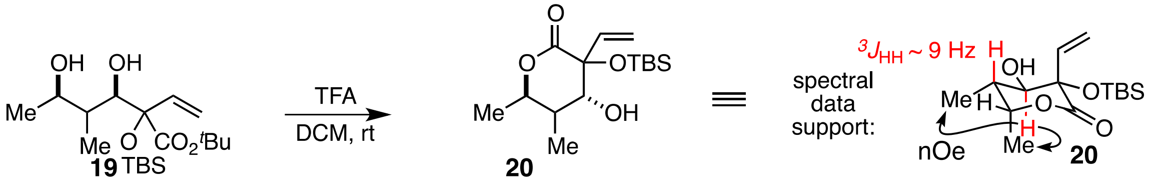

(4*R*,6*R*)-3-(*tert*-butyldimethylsilyloxy)-4-hydroxy-5,6-dimethyl-3-vinyltetrahydro-2*H*-pyran-2-one (20). To a solution of **19** (30 mg, 0.800 mmol, 1 equiv) in DCM (0.67 mL) was added trifluoroacetic acid (0.33 mL). The reaction was stirred at room temperature until TLC analysis indicated consumption of the starting material **20**. The reaction was concentrated *in*

vacuo, redissolved in $CHCl_3$, and concentrated again (repeated twice more to remove the TFA). The residue was purified via flash column chromatography using 10% EtOAc/hexanes to afford 10 mg (0.033 mmol, 41% yield) of **20** as a clear oil. Analytical data for **20**: **IR** (thin film, cm^{-1}): 3461, 2929, 2856, 2359, 1747, 1633, 1463, 1250, 1159, 1056, 839, 781; **^{1}H NMR** (600 MHz, $CDCl_3$): δ 6.12 (dd, *J* = 17.4, 10.8 Hz, 1H), 5.42 (d, *J* = 10.8, 1H), 5.38 (d, *J* = 17.4 Hz, 1H), 4.69 (quint, *J* = 6.6 Hz, 1H), 3.74 (d, *J* = 10.2 Hz, 1H), 2.24-2.19 (m, 1H), 2.19 (s, 1H), 1.30 (d, *J* = 6.6 Hz, 3H), 1.08 (d, *J* = 7.2 Hz, 3H), 0.91 (s, 9H), 0.18 (s, 3H) 0.16 (s, 3H); **^{13}C NMR** (150 MHz, $CDCl_3$): δ 172.0, 134.2, 119.8, 81.2, 76.5, 35.6, 26.0, 18.6, 17.2, 13.2, −2.8, −3.0 (one coincident resonance); **TLC** (10% EtOAc/hexanes), R_f 0.11 (CAM); **LRMS** (ESI): Calcd. for $C_{15}H_{28}O_4Si$+Na: 323.17, Found: 323.16

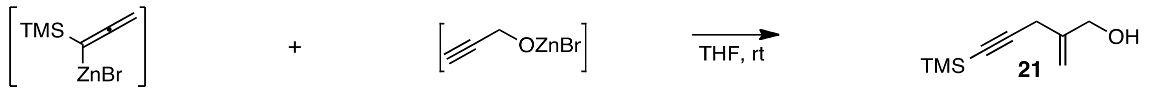

2-methylene-5-(trimethylsilyl)pent-4-yn-1-ol (21). This procedure is based on a literature precedent for which a detailed procedure is lacking [27]. The allenylzinc reagent was generated from trimethylsilyl propargyl bromide [28] as follows: to a suspension of Zn^0 (6.29 g, 96 mmol, 2.0 equiv) in dry THF (100mL) under N_2 was slowly added Br_2 (0.49 mL, 1.53 g, 9.6 mmol, 10 mol % relative to Zn^0) to activate the metal surface. The suspension was heated under reflux for 15 min and then cooled to room temperature, at which point the TMS propargyl bromide (9.2 g, 48 mmol, 1.0 equiv) was added dropwise. The mixture was stirred overnight (12 h) under N_2, and then stirring was stopped to allow the excess Zn^0 to settle. The solution was titrated with I_2 [29], and the amount of active allenylzinc was calculated: in this case, it was 29.6 mmol active reagent (62% yield; typically approx. 60% yield of active reagent was observed over several runs). Based on the calculation, 1.0 equiv of propargyl alcohol (1.72 mL, 1.66 g, 29.6 mmol) was added to a separate dry flask containing THF 60 mL, which was cooled to −78 ºC. A solution of *n*-butyllithium (1.5 M in hexanes, 21.7 mL, 32.5 mmol, 1.1 equiv) was added, and the solution is warmed to room temperature for 30 min, after which it was cooled again to −78 ºC. A solution of $ZnBr_2$ (7.33 g, 32.5 mmol, 1.1 equiv) in THF (40 mL) was transferred to the lithium alkoxide, which was warmed again to room temperature for 30 min. The allenylzinc solution was transferred to the solution of zinc alkoxide via cannula, and the reaction was stirred overnight (12 h). The reaction was quenched with a solution of NH_4Cl (50 mL), and diluted with H_2O (500 mL). The layers are separated, and the aqueous layer was extracted with Et_2O (3 × 75 mL). The combined organic extracts were washed with brine (50 mL), dried over $MgSO_4$, and concentrated *in vacuo*. The crude residue was purified by flash column chromatography using 10% EtOAc/hexanes to afford 2.64 g (15.7 mmol, 53% yield) of the product **21** as a colorless oil (reported: 80% yield; best run in our hands: 72% yield). **21** is a known compound, but its ^{1}H NMR has not been reported in $CDCl_3$ before: **^{1}H NMR** ($CDCl_3$, 300 MHz): δ 5.21 (s, 1H), 5.15 (s, 1H), 4.15 (d, *J* = 6.3 Hz, 2H), 3.06 (s, 2H), 1.50 (d, *J* = 6.3 Hz, 1H), 0.17 (s, 9H); **TLC** (20% EtOAc/hexanes) 0.28 ($KMnO_4$/CAM).

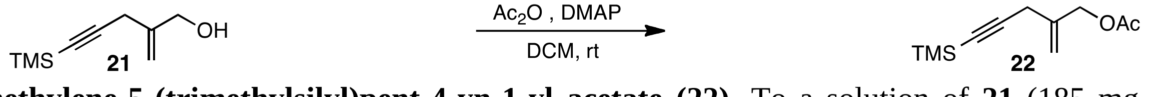

2-methylene-5-(trimethylsilyl)pent-4-yn-1-yl acetate (22). To a solution of **21** (185 mg, 1.1 mmol, 1.0 equiv), *N*,*N*-dimethylaminopyridine (27 mg, 0.22 mmol, 0.20 equiv), and triethylamine (278 mg, 2.75 mmol, 2.5 equiv) at 0 ºC in DCM (12 mL) was added acetic anhydride (224 mg, 2.2 mmol, 2 equiv) neat, dropwise. Dry solvent and N_2 atmosphere were not

necessary. The solution was stirred until TLC analysis indicated consumption of **21** (≤ 1 h), at which point the solution was carefully concentrated *in vacuo*. The crude oil was purified via flash column chromatography, eluting with 10% Et_2O/pentanes to afford 220 mg (1.045 mmol, 95% yield) of **22** as a clear, colorless oil. Analytical data for **22**: **IR** (thin film, cm^{-1}): 3090, 2960, 2899, 2179, 1744, 1658, 1419, 1373, 1249, 1233, 1030, 913, 844, 761; **^{1}H NMR** (400 MHz, $CDCl_3$): δ (5.32 (s, 1H), 5.18 (s, 1H), 4.58 (s, 2H), 3.03 (s, 2H), 2.08 (s, 3H), 0.16 (s, 9H); **^{13}C NMR** (100 MHz, $CDCl_3$): δ 170.6, 138.5, 114.9, 102.4, 87.9, 66.3, 24.5, 20.9, 0.01; **TLC** (20% EtOAc/hexanes), R_f 0.62 ($KMnO_4$/CAM); **LRMS** (ESI): Calcd. for $C_{11}H_{18}O_2Si$+Na: 233.16 Found: 233.10.

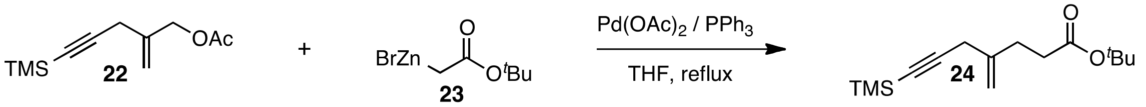

***tert*-butyl 4-methylene-7-(trimethylsilyl)hept-6-ynoate (24).** To a suspension of Zn^0 (3.92 g, 60 mmol, 2 equiv) in THF (60 mL) under N_2 was added Br_2 (0.31 mL, 0.967 g, 6 mmol, 0.1 equiv relative to Zn^0) to activate the metal surface. The suspension was heated under reflux, and *tert*-butyl bromoacetate (4.43 mL, 5.85g, 30 mmol, 1 equiv) was added slowly over 15 min via syringe pump. The reaction mixture was heated under reflux for 1 h, and then heating and stirring were stopped to allow the solution to cool and the excess Zn^0 to settle. The Reformatsky reagent **23** thus generated was titrated with I_2 [29]; on this occasion, it was 0.45 M (90% yield of active reagent; this was typical). In a separate flask, palladium acetate (107 mg, 0.475 mmol, 0.05 equiv), triphenylphosphine (249 mg, 0.95 mmol, 0.10 equiv), and the allylic acetate **22** (2 g, 9.5 mmol, 1.0 equiv) were mixed in THF (60 mL). To this mixture was added the Reformatsky reagent (31.7 mL, 14.3 mmol, 1.5 equiv), and the resulting solution was heated under reflux overnight (12 h). TLC analysis indicated consumption of the acetate **22**, and the reaction was quenched with sat. aq NH_4Cl (50 mL) and diluted with H_2O (250 mL). The layers were shaken and separated, and the aqueous layer was extracted with Et_2O (3 × 50 mL). The combined organic extracts were washed with H_2O (50 mL) and brine (50 mL), dried over $MgSO_4$, and concentrated *in vacuo*. Purification of the residue by flash column chromatography using 2.5% EtOAc/hexanes afforded 2.1 g (7.88 mmol, 83% yield) of **24** as a clear, slightly pale yellow oil. Analytical data for **24**: **IR** (thin film, cm^{-1}): 3084, 2963, 2900, 2177, 1732, 1652, 1367, 1251, 1148, 1033, 900, 844, 760; **^{1}H NMR** (600 MHz, $CDCl_3$): δ 5.10 (s, 1H), 4.85 (s, 1H), 2.97 (s, 2H), 2.40-2.35 (m, 4H), 1.43 (s, 9H), 0.15 (s, 9H); **^{13}C NMR** (150 MHz, $CDCl_3$): δ 172.3, 142.5, 111.3, 103.6, 87.3, 80.3, 33.7, 30.7, 28.1, 27.3, 0.0; **TLC** (5% EtOAc/hexanes), R_f 0.41 ($KMnO_4$/CAM); **LRMS** (ESI): Calcd. for $C_{15}H_{26}O_2Si$+Na: 289.16 Found: 289.17.

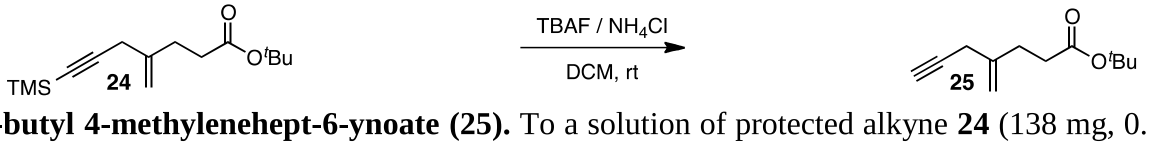

***tert*-butyl 4-methylenehept-6-ynoate (25).** To a solution of protected alkyne **24** (138 mg, 0.518 mmol, 1.0 equiv) in DCM (10 mL) was added NH_4Cl (138 mg, 2.6 mmol, 5 equiv), to make a fine suspension. To this mixture was added tetrabutylammonium fluoride (1 M in THF, 1.55 mL, 1.55 mmol, 3 equiv), and the reaction was monitored by removal of aliquots for NMR analysis. Upon completion, the reaction mixture was filtered and carefully concentrated *in vacuo*. The crude oil was purified by flash column chromatography using 5% Et_2O/pentanes to afford 95 mg (0.489 mmol, 95% yield) of the free alkyne **25** as a clear, pale yellow oil. Similar results were obtained on a larger scale (approx. 2 g). Analytical data for **25**: **IR** (thin film, cm^{-1}): 3086, 2979, 2931, 2121, 1730, 1654, 1368, 1255, 1148, 901, 847; **^{1}H NMR** (400 MHz, $CDCl_3$): δ 5.10 (s,

1H), 4.85 (s, 1H), 2.92 (br s, 2H), 2.40-2.35 (m, 4H), 2.11 (t, *J* = 2.4 Hz, 1H), 1.42 (s, 9H); **^{13}C NMR** (100 MHz, $CDCl_3$): δ 172.2, 142.3, 111.4, 81.1, 80.3, 70.8, 33.6, 30.6, 28.1, 25.9; **TLC** (10% EtOAc/hexanes), R_f 0.50 ($KMnO_4$/CAM); **LRMS** (ESI): Calcd. for $C_{12}H_{18}O_2$+Na: 217.12 Found: 217.13.

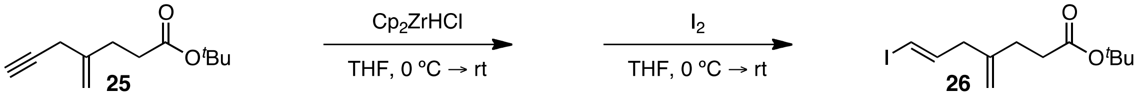

(*E*)-*tert*-butyl 7-iodo-4-methylenehept-6-enoate (26). To a suspension of zirconocene hydrochloride (520 mg, 2 mmol, 1.4 equiv) in THF (10 mL) under N_2 at 0 ºC was added a solution of the free alkyne **25** (280 mg, 1.44 mmol, 1 equiv) in THF (1.5 mL). The suspension was warmed to room temperature and stirred for 30 min past the point when the suspension had cleared (approx. 1.5 h total), at which point the solution was cooled once again to 0 ºC. A solution of iodine (585 mg, 2.3 mmol, 1.6 equiv) in THF (5 mL) was added to the reaction mixture, and the reddish color eventually persisted. The reaction was stirred for 30 min then poured into 100 mL of 1:1 sat. aq $NaHCO_3$: sat. aq $Na_2S_2O_3$, and stirred vigorously until the I_2 color had dissipated. The layers were separated, and the aqueous layer was extracted with Et_2O (3 × 25 mL). The combined organic extracts were washed with H_2O (20 mL) and brine (20 mL), dried over $MgSO_4$, and concentrated *in vacuo*. The residue was purified by flash column chromatography using 5% EtOAc/hexanes to afford 400 mg (1.24 mmol, 86% yield) of the vinyl iodide **26** as a clear, pale yellow oil. Analytical data for **26**: **IR** (thin film, cm^{-1}): 3080, 3050, 2978, 2930, 1730, 1649, 1604, 1434, 1391, 1367, 1252, 1150, 953, 898, 845; **^{1}H NMR** (400 MHz, $CDCl_3$): δ 6.50 (m, 1H), 6.07 (d, *J* = 14.4 Hz, 1H), 4.80 (s, 2H), 2.75 (d, *J* = 6.8 Hz), 2.35 (m, 2H), 2.28 (d, *J* = 7.2 Hz, 2H), 1.43 (s, 9H); **^{13}C NMR** (100 MHz, $CDCl_3$): δ 172.3, 144.9, 143.5, 111.3, 80.4, 76.4, 42.8, 33.6, 30.8, 28.1; **TLC** (5% EtOAc/hexanes), R_f 0.53 (UV/CAM); **LRMS** (ESI): Calcd. for $C_{12}H_{19}IO_2$+Na: 345.03 Found: 345.04

General procedure B: Three component coupling reactions with substituted vinyl nucleophiles generated by Mg/I exchange

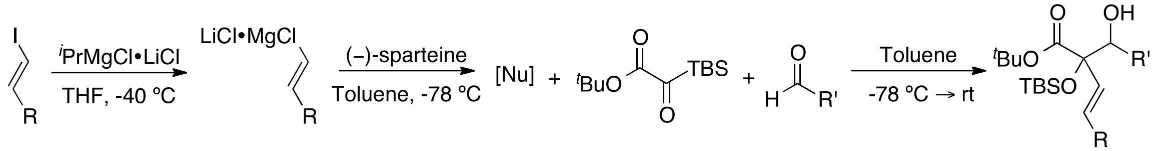

The procedure for the generation of the nucleophile was analogous to the literature procedure [30]. It was used for all of the reactions in **Scheme S-7**, although full details for these will not be reported here. To a solution of the vinyl iodide (1.5 equiv) in a minimal amount of dry THF (≤ 0.20 mL) cooled to −78 ºC under N_2 was added a solution of iPrMgCl·LiCl (1.65 equiv), and the reaction was stirred overnight (12 h) in a cryocool set to −40 ºC. The solution of the vinyl nucleophile thus generated was cooled to −78 ºC, a solution of (–)-sparteine (1.5 equiv) in toluene was added, and the solution was stirred at −78 ºC. Meanwhile, a separate solution of the silyl glyoxylate **1a** (1.0 equiv) and the aldehyde (**3** or **16**; 1.5 equiv) in dry toluene was prepared and cooled to −78 ºC for 15 min. The nucleophile/sparteine solution was transferred to the silyl glyoxylate/aldehyde solution via cannula, and the reaction mixture was stirred at −78 ºC for an additional 15 min followed by warming to room temperature for 1.5 h. The reaction was quenched with 10% (v/v) aq AcOH and diluted with H_2O. The layers were separated, and the aqueous layer was extracted with Et_2O (3×). The combined organic extracts were washed with

H_2O and brine, dried over $MgSO_4$, and concentrated in vacuo. The residue was purified by flash column chromatography using the indicated eluent system.

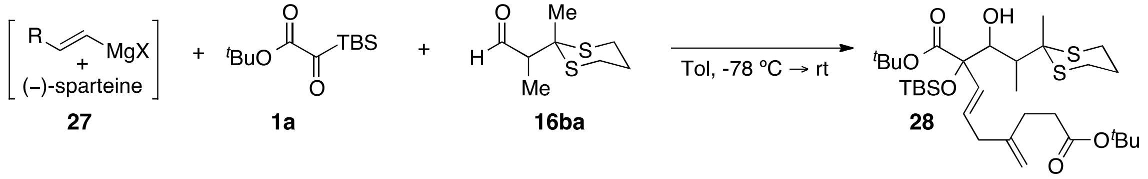

(*E*)-di-*tert*-butyl 2-(*tert*-butyldimethylsilyloxy)-2-(1-hydroxy-2-(2-methyl-1,3-dithian-2-yl)propyl)-6-methylenenon-3-enedioate (35e). The title compound was prepared according to General Procedure B using **36'** (74 mg, 0.230 mmol, 1.5 equiv) in THF (0.200 mL), and ^{i}PrMgCl·LiCl (1.3 M in THF, 0.19 mL, 0.253 mmol, 1.65 equiv). After overnight nucleophile generation, (–)-sparteine (54 mg, 0.230 mmol, 1.5 equiv) in toluene (1 mL) was used to complex the Grignard. The silyl glyoxylate **16a** (37 mg, 0.153 mmol, 1.0 equiv) and dithiane aldehyde **32cb** (44 mg, 0.230 mmol, 1.5 equiv) solution was prepared in toluene (3 mL). Purification was effected using 2.5% Et_2O/hexanes to afford 53 mg (0.0840 mmol, 55% yield) of the desired product as a clear oil. Analytical data for **35e**: **^{1}H NMR** (600 MHz, $CDCl_3$): δ 5.90 (m, 1H), 5.62 (d, *J* = 15.6 Hz, 1H), 4.77 (s, 1H), 4.75 (s, 1H), 4.55 (d, *J* = 10.8 Hz, 1H), 2.78 (d, *J* = 8.4 Hz, 1H), 2.80-2.65 (m, 4H), 2.47 (q, *J* = 7.2 Hz, 1H), 2.37-2.30 (m, 3H), 2.30-2.25 (m, 2H), 1.95-1.85 (m, 2H), 1.53 (s, 3H), 1.49 (s, 9H), 1.43 (s, 9H), 1.13 (d, *J* = 7.2 Hz, 3H), 0.93 (s, 9H), 0.23 (s, 3H), 0.11 (s, 3H); **^{13}C NMR** (150 MHz, $CDCl_3$): δ 172.3, 171.5, 146.3, 131.9, 129.6, 110.5, 85.1, 82.3, 80.2, 74.7, 54.3, 39.4, 33.7, 31.2, 28.1, 28.0, 26.8, 26.3, 26.2, 25.7, 24.9, 23.9, 19.3, 9.5, −1.99, −2.00; **TLC** (5% EtOAc/hexanes) R_f 0.18 (UV/CAM); **LRMS** (ESI): Calcd. for $C_{32}H_{58}O_6S_2Si$+Na: 653.33, Found: 653.33; Calcd. for $C_{32}H_{58}O_6S_2Si$+Cs: 763.25, Found: 763.24.

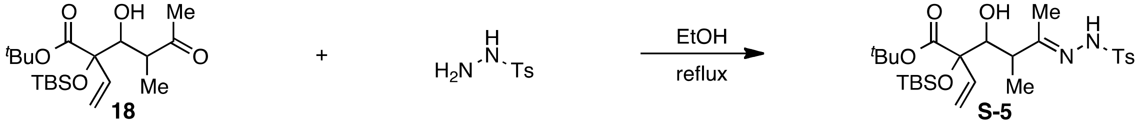

(*E*)-*tert*-butyl 2-((*tert*-butyldimethylsilyl)oxy)-3-hydroxy-4-methyl-5-(2-tosylhydrazono)-2-vinylhexanoate (S-5). To a solution of ketone **18** (100 mg, 0.268 mmol, 1.0 equiv) in EtOH (5 mL) was added *p*-toluenesulfonylhydrazide (50 mg, 0.268 mmol, 1.0 equiv). The mixture was heated under reflux for 10 min and cooled to room temperature and concentrated *in vacuo*. The residue was purified by flash column chromatography using a 10→20% EtOAc/hexanes gradient to afford 123 mg (0.227 mmol, 85% yield) of the tosylhydrazone **S-5** as a white foam. Analytical data for **S-5**: **IR** (thin film, cm^{-1}): 3327, 2956, 2930, 2856, 2253, 1744, 1708, 1640, 1460, 1369, 1252, 1166, 916, 840; **^{1}H NMR** (600 MHz, $CDCl_3$): δ 7.83 (d, *J* = 8.4 Hz, 1H), 7.29 (d, *J* = 8.4 Hz, 1H), 5.54 (dd, *J* = 17.4, 11.4 Hz, 1H), 5.22 (d, *J* = 17.4, 1H), 4. 90 (d, *J* = 11.4, 1H), 4.04 (d, *J* = 7.2 Hz, 1H), 2.64 (app quint, *J* = 7.2 Hz, 1H), 2.41 (s, 3H), 1.67 (s, 3H), 1.47 (s, 9H), 1.01 (d, *J* = 6.6 Hz, 3H), 0.91 (s, 9H), 0.18 (s, 3H), 0.10 (s, 3H); **^{13}C NMR** (150 MHz, $CDCl_3$): δ 170.9, 160.7, 143.8, 137.7, 135.6, 129.4, 128.1, 114.9, 84.3, 82.5, 76.7, 42.8, 27.9, 26.4, 21.6, 19.3, 15.1, 14.6, −2.3, −2.6; **TLC** (20% EtOAc/hexanes), R_f0.21 (UV/CAM).

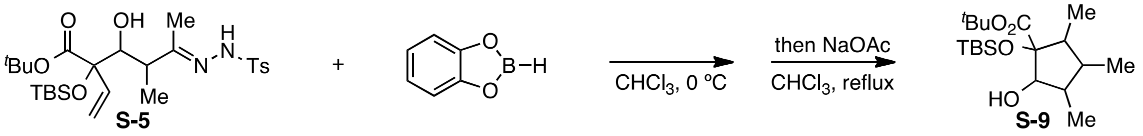

***tert*-butyl 1-(*tert*-butyldimethylsilyloxy)-2-hydroxy-3,4,5-trimethylcyclopentane-1-carboxylate (S-9).** This reaction was performed analogously to a literature procedure. To a solution of **S-5** (12 mg, 0.0222 mmol, 1.0 equiv) in dry $CHCl_3$ (3 mL) under N_2 at 0 °C was

added a solution of catecholborane (1M in THF, 0.05 mL, 0.05 mmol, 2.3 equiv). The mixture was stirred at 0 ºC until TLC analysis indicated consumption of **S-5**, at which point $NaOAc \cdot 3H_2O$ (21 mg, 0.153 mmol, 6.9 equiv) was added to the flask, which was affixed with a reflux condenser. The reaction was heated under reflux for 1 h. TLC analysis indicated consumption of the intermediate and formation of the product, and the cloudy reaction mixture was cooled to room temperature. The mixture was filtered through Celite and concentrated *in vacuo*. Purification by flash column chromatography using 2.5% EtOAc/hexanes afforded the product as a mixture of diastereomers. The yield was not determined, but **S-9** was the sole product obtained and none of the desired reduction product **5** was obtained. Preparative HPLC using 2.5% EtOAc/hexanes afforded a single diastereomer, which was characterized as follows: analytical data for **S-9**: **IR** (thin film, cm^{-1}): 3458, 2956, 2929, 2856, 2090, 1737, 1696, 1645, 1461, 1369, 1251, 1157, 1048, 839, 779; **^{1}H NMR** (600 MHz, $CDCl_3$): δ 3.83 (t, J = 10.2 Hz, 1H), 2.36-2.30 (m, 1H), 1.66 (d, J = 10.2 Hz, 1H), 1.67-1.59 (m, 1H), 1.56-1.45 (m, 1H), 1.47 (s, 9H), 1.09 (d, J = 6.6 Hz, 1H), 0.92 (d, J = 7.2 Hz, H), 0.92 (s, 9H), 0.84 (d, J = 7.2 Hz, 1H), 0.22 (s, 3H) 0.15 (s, 3H); **^{13}C NMR** (150 MHz, $CDCl_3$): δ 172.9, 87.4, 82.9, 81.4, 47.3, 41.0, 39.2, 28.0, 26.5, 19.6, 16.9, 16.0, 9.5, −2.2, −2.7; **TLC** (5% EtOAc/hexanes), R_f 0.27 (CAM); **LRMS** (ESI): Calcd. for $C_{19}H_{38}O_4Si$+Na: 381.24, Found: 381.24; Calcd. for $C_{19}H_{38}O_4Si$+Cs: 491.16, Found: 491.15.

References:

[1] Giampietro, N. C.; Kampf, J. W.; Wolfe, J. P. *J. Am. Chem. Soc.* **2009**, *131*, 12556-12557.
[2] Greszler, S. N.; Johnson, J. S. *Angew. Chem. Int. Ed*. **2009**, *48*, 3689-3691.
[3] Kobayashi, Y.; Kitano, Y.; Takeda, Y.; Sato, F. *Tetrahedron* **1986**, *42*, 2937-2943.
[4] Sato, F.; Kusakabe, M.; Kobayashi, Y. *J. Chem. Soc., Chem. Commun.* **1984**, 1130.
[5] Sato, F.; Takeda, Y.; Uchiyama, H.; Kobayashi, Y. *J. Chem. Soc., Chem. Commun.* **1984**, 1132.
[6] Sato, F.; Kusakabe, M.; Kato, T.; Kobayashi, Y. *J. Chem. Soc., Chem. Commun.* **1984**, 1331.
[7] Tucker, C. E.; Majid, T. N.; Knochel, P. *J. Am. Chem. Soc.* **1992**, *114*, 3983-3985.
[8] Wipf, P.; Ribe, S. *J. Org. Chem.* **1998**, *63*, 6454-6455.
[9] Wipf, P.; Xu, W. *Tetrahedron Lett.* **1994**, *35*, 5197-5200.
[10] Schmitt, D. C.; Johnson, J. S. *Org. Lett.* **2010**, *12*, 944-947.
[11] Yang, T.-K.; Lee, D.-S.; Haas, J. Raney Nickel. *e-EROS Encyclopedia of Reagents for Organic Synthesis* **2001**.
[12] Back, T. G.; Baron, D. L.; Yang, K. *J. Org. Chem.* **1993**, *58*, 2407-2413.
[13] Chan, M. C.; Cheng, K. M.; Ho, K. M.; Ng, C. T.; Yam, T. M.; Wang, B. S. L.; Luh, T. Y. *J. Org. Chem.* **1988**, *53*, 4466-4471
[14] Becker, S.; Fort, Y.; Vanderesse, R.; Caubere, P. *Tetrahedron Letters* **1988**, *29*, 2963-2966.
[15] Becker, S.; Fort, Y.; Caubere, P. *J. Org. Chem.* **1990**, *55*, 6194-6198.
[16] Kabalka, G. W.; Baker, J. D. *J. Org. Chem.* **1975**, *40*, 1834-1835.
[17] Anelli, P. L.; Montanari, F.; Quici, S.; Nonoshita, K.; Yamamoto, H. *Org. Synth.* **1990**, *69*, 212.
[18] Fürstner, A.; Flügge, S.; Larionov, O.; Takahashi, Y.; Kubota, T.; Kobayashi, J. *Chem. Eur. J.* **2009**, *15*, 4011-4029.
[19] Ghosh, A. K.; Gong, G. *Chem. Asian J.* **2008**, *3*, 1811-1823.
[20] Saito, T.; Fuwa, H.; Sasaki, M. *Tetrahedron* **2011**, *67*, 429-445.
[21] Hara, S.; Dojo, H.; Takinami, S.; Suzuki, A. *Tetrahedron Lett.* **1983**, *24*, 731-734.
[22] Yanagisawa, A.; Habaue, S.; Yamamoto, H. *J. Am. Chem. Soc.* **1989**, *111*, 366-368.
[23] Firouzabadi, H.; Iranpoor, N.; Hazarkhani, H. *J. Org. Chem.* **2001**, *66*, 7527-7529.
[24] Stiles, M.; Wolf, D.; Hudson, G. V. *J. Am. Chem. Soc.* **1959**, *81*, 628-632.
[25] Nickon, A.; Rodriguez, A. D.; Shirhatti, V.; Ganguly, R. *J. Org. Chem.* **1985**, *50*, 4218-4226.
[26] Kim, H.; Baker, J. B.; Lee, S.-U.; Park, Y.; Bolduc, K. L.; Park, H.-B.; Dickens, M. G.; Lee, D.-S.; Kim, Y.; Kim, S. H.; Hong, J. *J. Am. Chem. Soc.* **2009**, *131*, 3192-3194.
[27] Renaud, J.-L.; Aubert, C.; Malacria, M. *Tetrahedron Lett.* **1999**, *40*, 5015-5018.
[28] Maclnnes, I.; Walton, J. C. *J. Chem. Soc., Perkin Trans. 2* **1987**, 1077.
[29] Krasovskiy, A.; Knochel, P. *Synthesis* **2006**, 890-891.
[30] Ren, H.; Krasovskiy, A.; Knochel, P. *Org. Lett.* **2004**, *6*, 4215-4217.

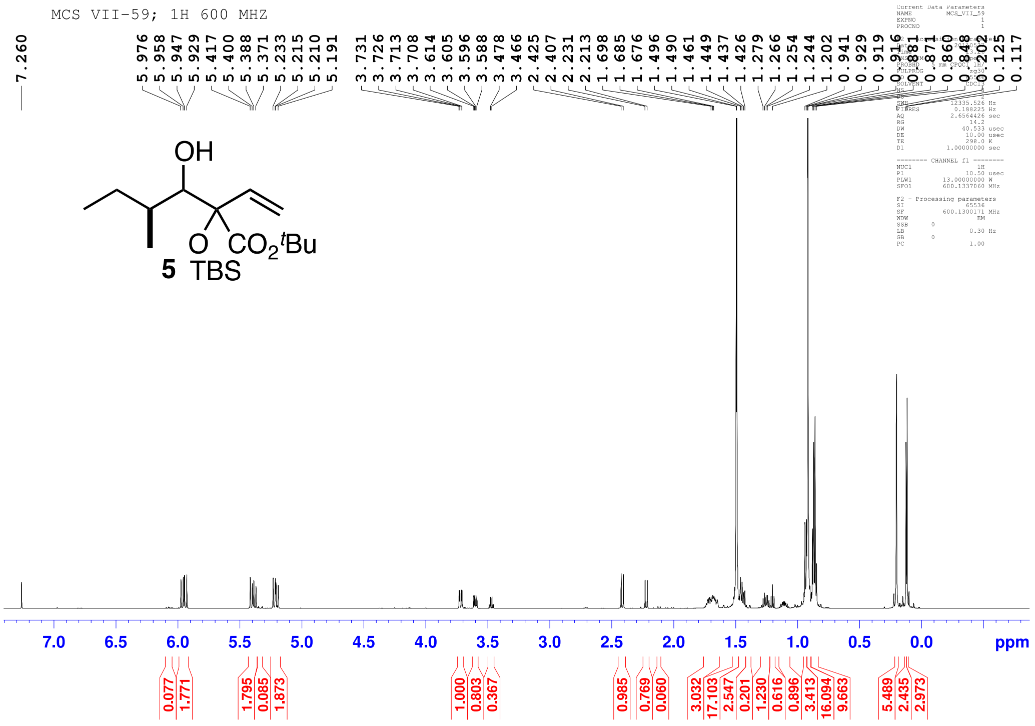
MCS VII-59; 1H 600 MHZ
7.260
5.976 5.958 5.947 5.929 5.417 5.400 5.388 5.371 5.233 5.215 5.210 5.191 3.731 3.726 3.713 3.708 3.614 3.605 3.596 3.588 3.478 3.466 2.425 2.407 2.231 2.213 1.698 1.685 1.676 1.496 1.490 1.461 1.449 1.437 1.426 1.279 1.266 1.254 1.244 1.202 0.941 0.929 0.919 0.916 0.881 0.871 0.860 0.848 0.202 0.125 0.117
OH
O
CO2tBu
5
TBS
7.0 6.5 6.0 5.5 5.0 4.5 4.0 3.5 3.0 2.5 2.0 1.5 1.0 0.5 0.0 ppm
0.077 1.771 1.795 0.085 1.873 1.000 0.803 0.367 0.985 0.769 0.060 3.032 17.103 2.547 0.201 1.230 0.616 0.896 3.413 16.094 9.663 5.489 2.435 2.973

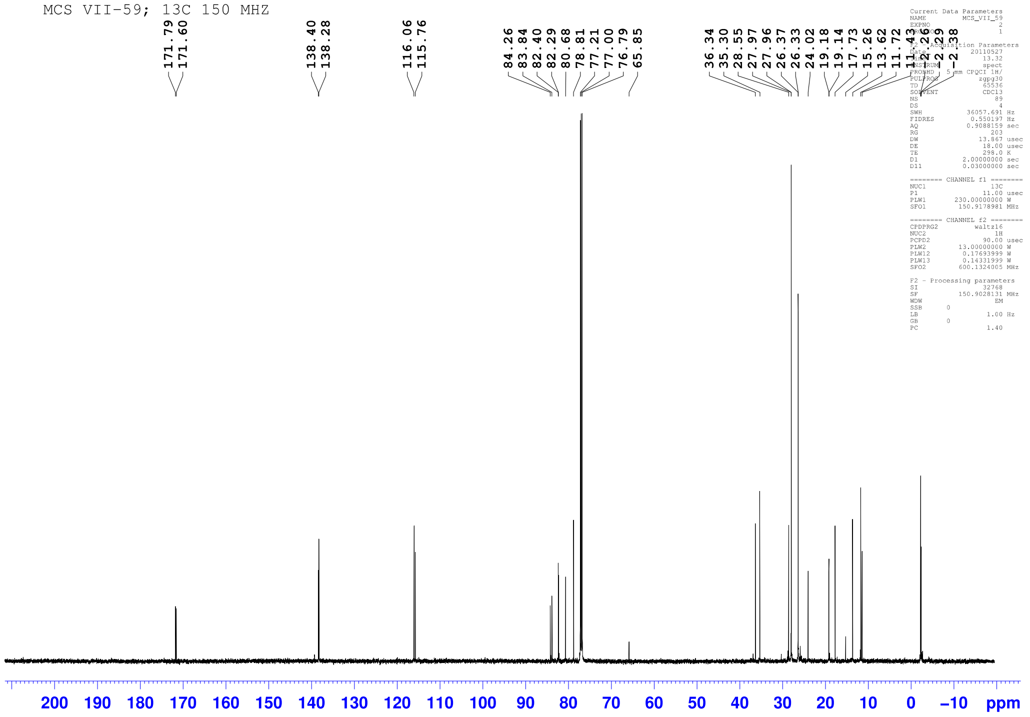
MCS VII-59; 13C 150 MHZ
171.79 171.60 138.40 138.28 116.06 115.76 84.26 83.84 82.40 82.29 80.68 78.81 77.21 77.00 76.79 65.85 36.34 35.30 28.55 27.97 27.96 26.37 26.33 24.02 19.18 19.14 17.73 15.26 13.62 11.72 11.43
200 190 180 170 160 150 140 130 120 110 100 90 80 70 60 50 40 30 20 10 0 −10 ppm

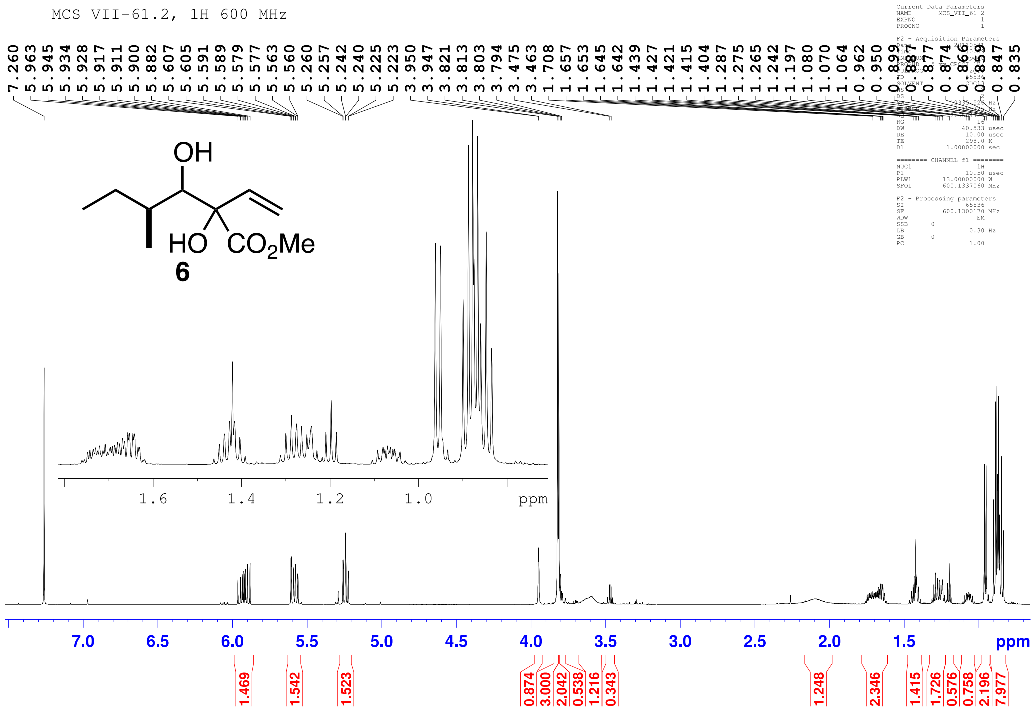
MCS VII-61.2, 1H 600 MHz
7.260 5.963 5.945 5.934 5.928 5.917 5.911 5.900 5.882 5.607 5.605 5.591 5.589 5.579 5.577 5.563 5.560 5.260 5.257 5.242 5.240 5.225 5.223 3.950 3.947 3.821 3.813 3.803 3.794 3.475 3.463 1.708 1.657 1.653 1.645 1.642 1.439 1.427 1.421 1.415 1.404 1.287 1.275 1.265 1.242 1.197 1.080 1.070 1.064 0.962 0.950 0.899 0.887 0.877 0.874 0.866 0.859 0.847 0.835
OH
HO
CO2Me
6
1.6 1.4 1.2 1.0 ppm
7.0 6.5 6.0 5.5 5.0 4.5 4.0 3.5 3.0 2.5 2.0 1.5 ppm
1.469 1.542 1.523 0.874 3.000 2.042 0.538 1.216 0.343 1.248 2.346 1.415 1.726 0.576 0.758 2.196 7.977

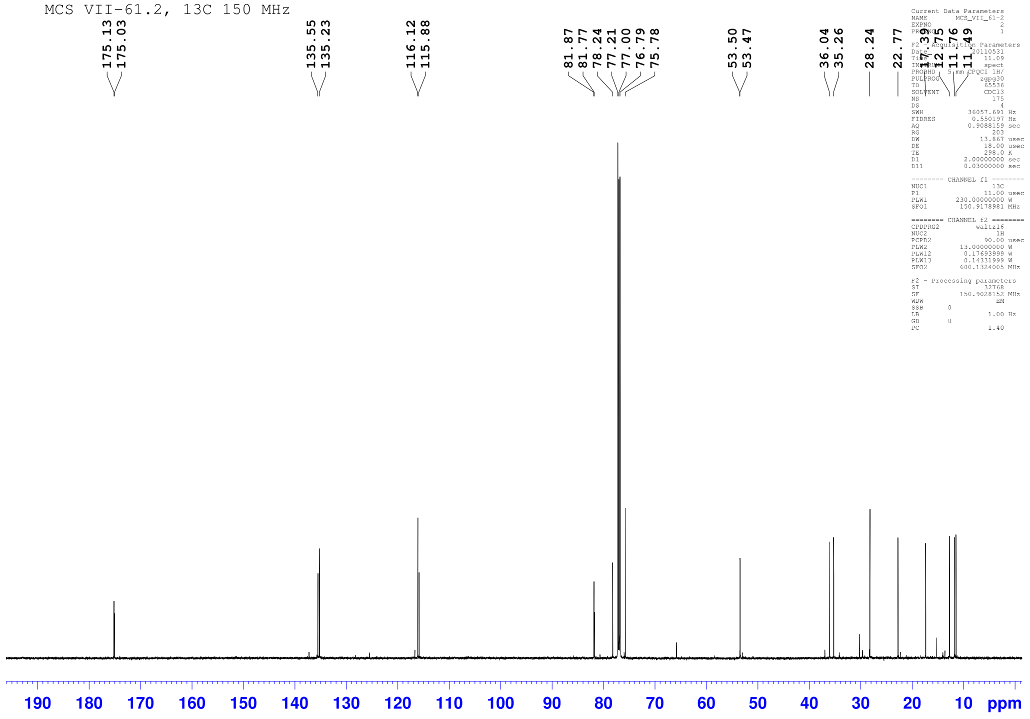
MCS VII-61.2, 13C 150 MHz
175.13 175.03 135.55 135.23 116.12 115.88 81.87 81.77 78.24 77.21 77.00 76.79 75.78 53.50 53.47 36.04 35.26 28.24 22.77 11.76 11.49
190 180 170 160 150 140 130 120 110 100 90 80 70 60 50 40 30 20 10 ppm

MCS IV-92; 1H 600 MHZ

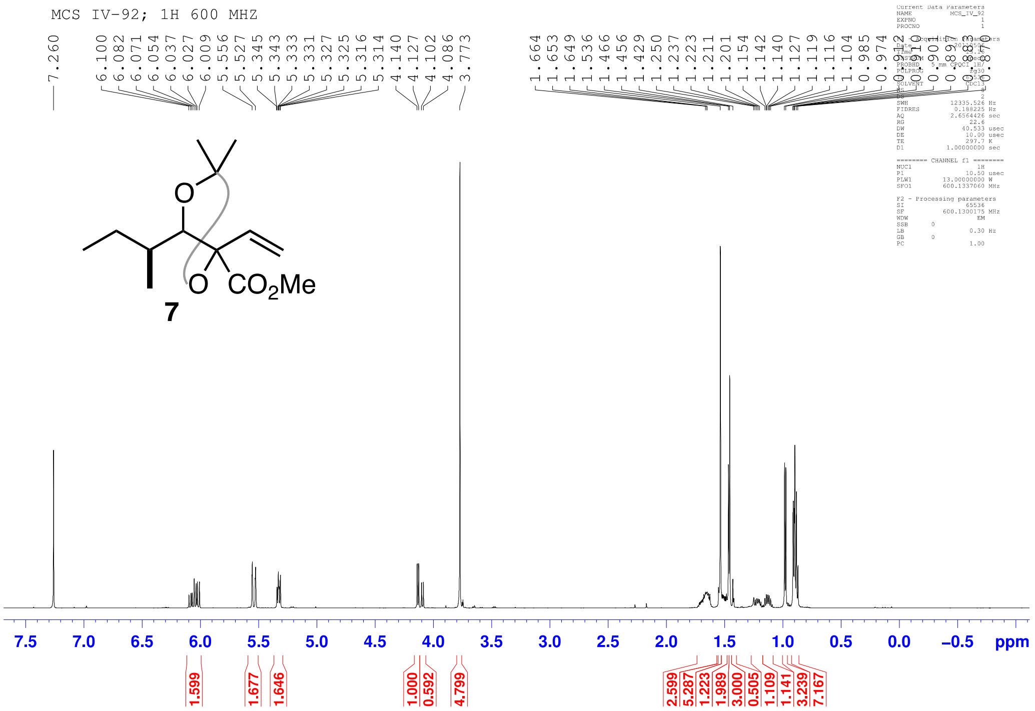

MCS IV-92; 13C, 150 MHz

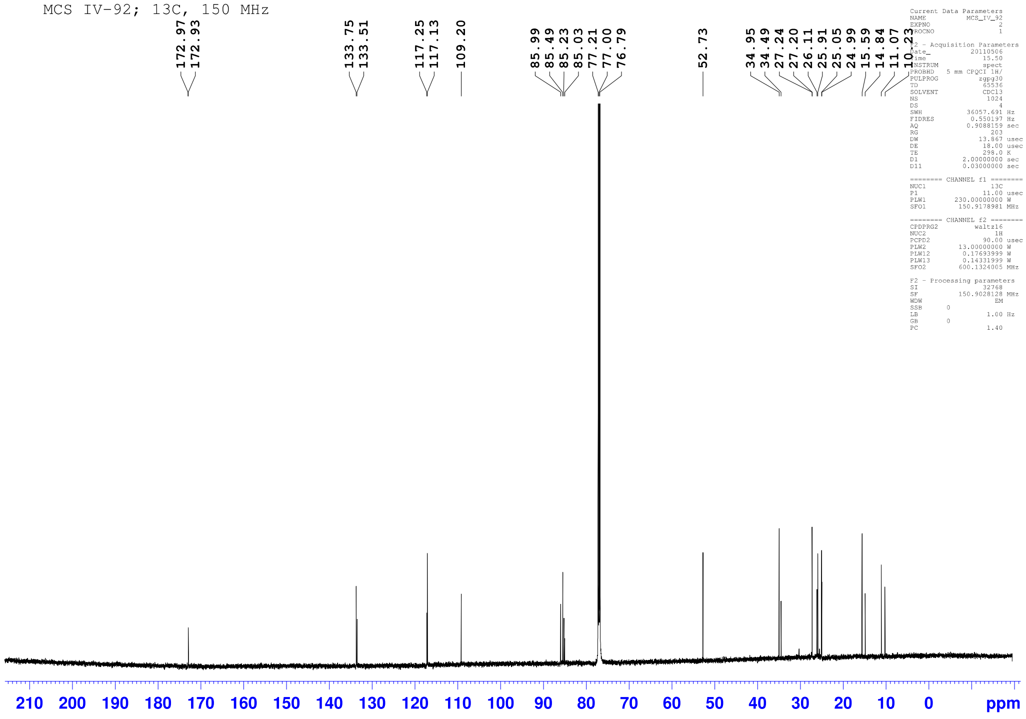

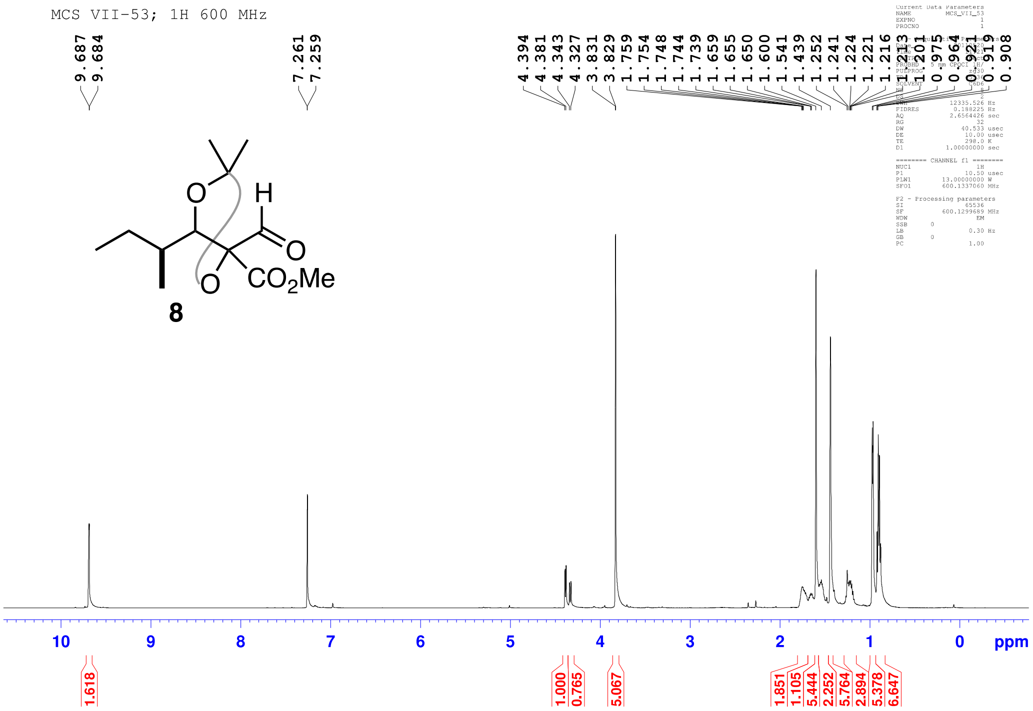
MCS VII-53; 1H 600 MHz
9.687
9.684
7.261
7.259
4.394
4.381
4.343
4.327
3.831
3.829
1.759
1.754
1.748
1.744
1.739
1.659
1.655
1.650
1.600
1.541
1.439
1.252
1.241
1.224
1.221
1.216
1.213
1.201
0.975
0.964
0.921
0.919
0.908
8
1.618
1.000
0.765
5.067
1.851
1.105
5.444
2.252
5.764
2.894
5.378
6.647
ppm

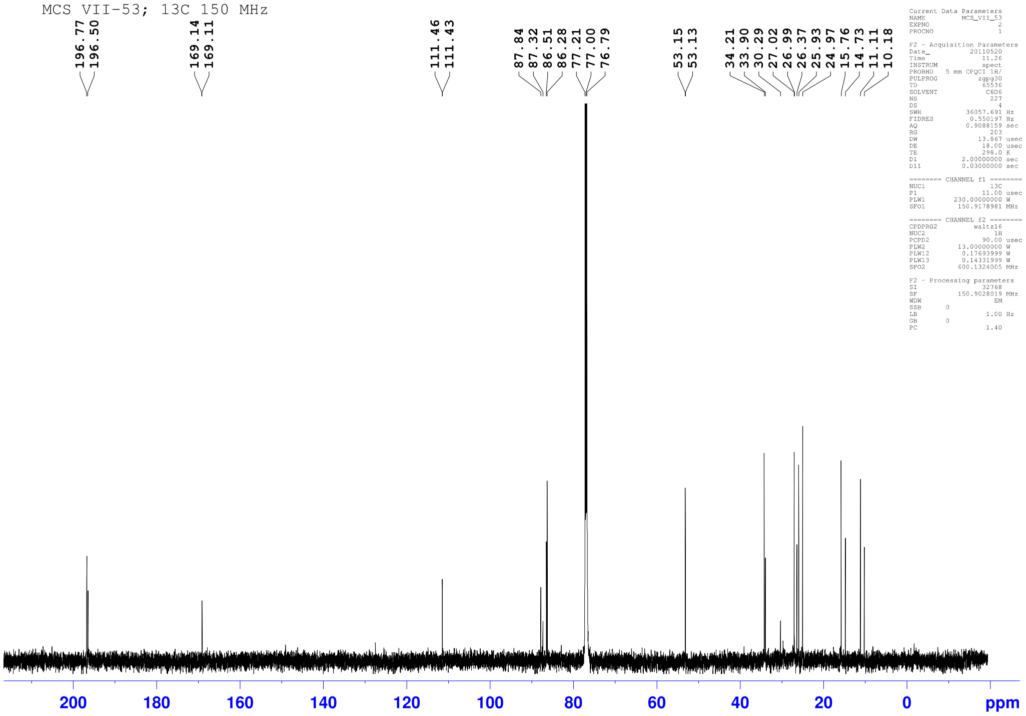
MCS VII-53; 13C 150 MHz
196.77
196.50
169.14
169.11
111.46
111.43
87.84
87.32
86.51
86.28
77.21
77.00
76.79
53.15
53.13
34.21
33.90
30.29
27.02
26.99
26.37
25.93
24.97
15.76
14.73
11.11
10.18
ppm

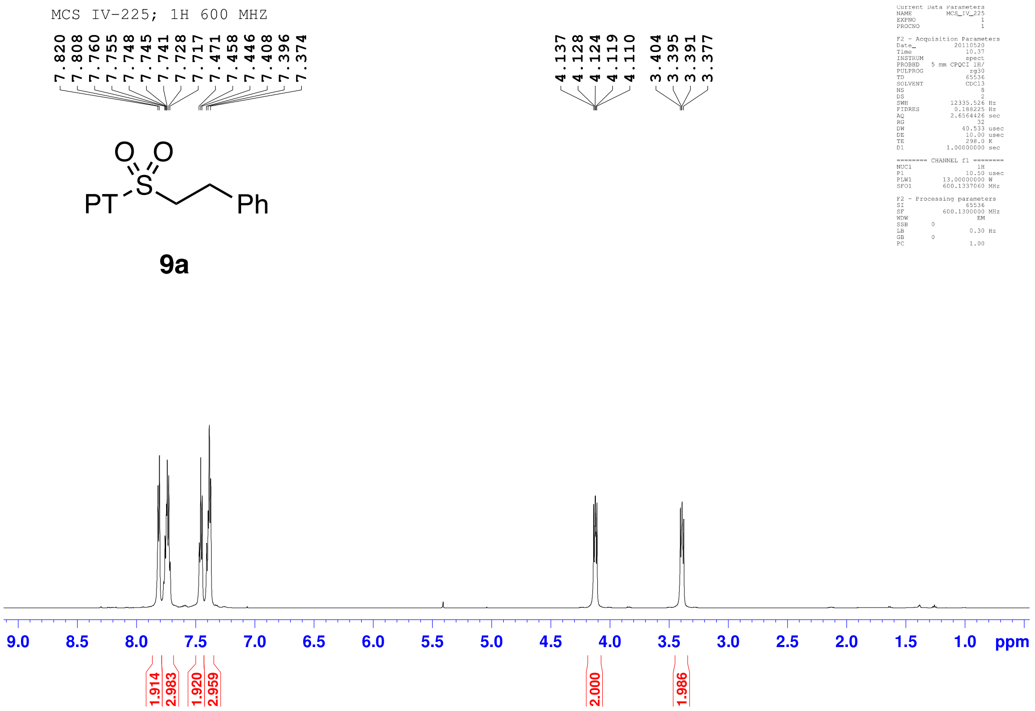
MCS IV-225; 1H 600 MHZ
7.820 7.808 7.760 7.755 7.748 7.745 7.741 7.728 7.717 7.471 7.458 7.446 7.408 7.396 7.374
4.137 4.128 4.124 4.119 4.110
3.404 3.395 3.391 3.377
O O
S
PT
Ph
9a
1.914 2.983 1.920 2.959
2.000
1.986
9.0 8.5 8.0 7.5 7.0 6.5 6.0 5.5 5.0 4.5 4.0 3.5 3.0 2.5 2.0 1.5 1.0 ppm

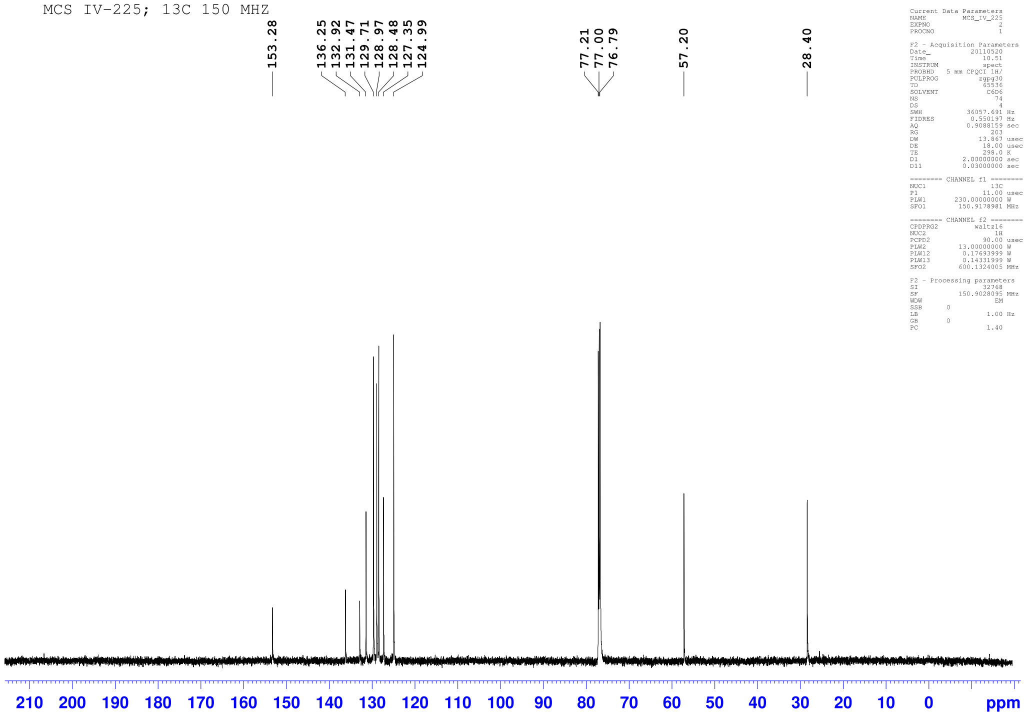
MCS IV-225; 13C 150 MHZ
153.28
136.25 132.92 131.47 129.71 128.97 128.48 127.35 124.99
77.21 77.00 76.79
57.20
28.40
210 200 190 180 170 160 150 140 130 120 110 100 90 80 70 60 50 40 30 20 10 0 ppm

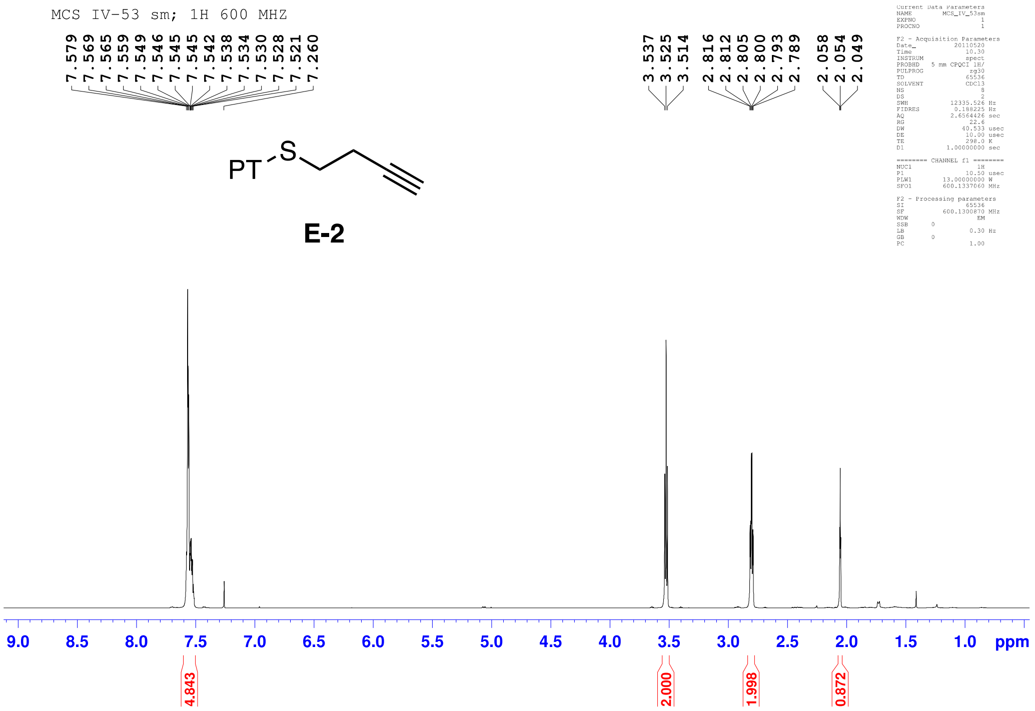
MCS IV-53 sm; 1H 600 MHZ
7.579 7.569 7.565 7.559 7.549 7.546 7.545 7.545 7.542 7.538 7.534 7.530 7.528 7.521 7.260
3.537 3.525 3.514
2.816 2.812 2.805 2.800 2.793 2.789
2.058 2.054 2.049
PT
S
E-2
9.0 8.5 8.0 7.5 7.0 6.5 6.0 5.5 5.0 4.5 4.0 3.5 3.0 2.5 2.0 1.5 1.0 ppm
4.843
2.000
1.998
0.872

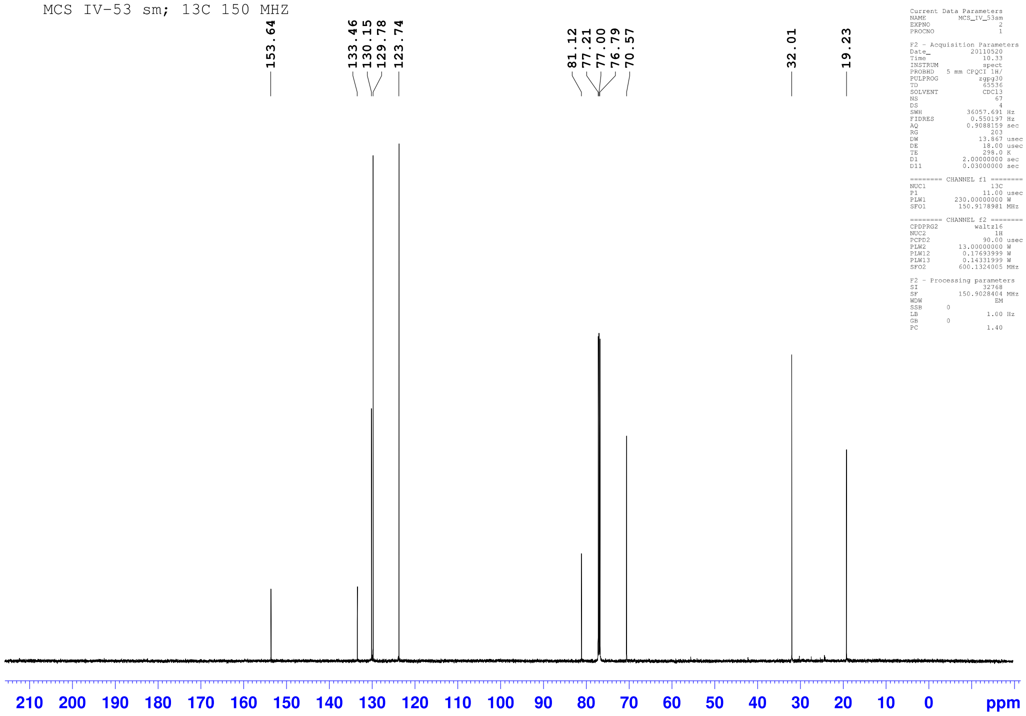
MCS IV-53 sm; 13C 150 MHZ
153.64
133.46 130.15 129.78 123.74
81.12 77.21 77.00 76.79 70.57
32.01
19.23
210 200 190 180 170 160 150 140 130 120 110 100 90 80 70 60 50 40 30 20 10 0 ppm

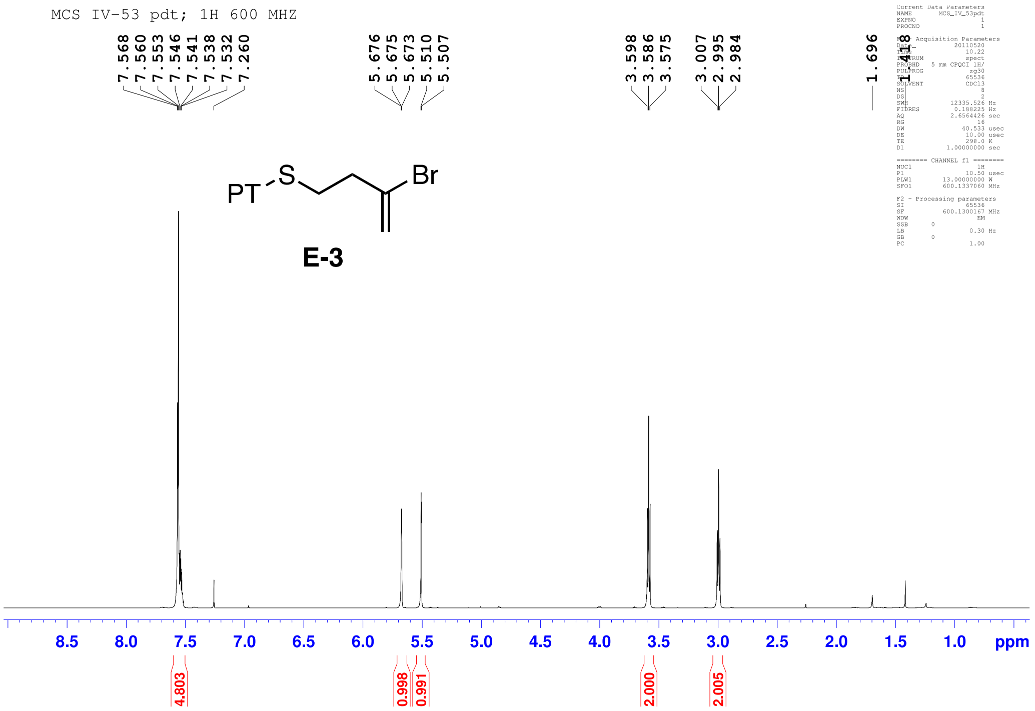
MCS IV-53 pdt; 1H 600 MHZ
7.568
7.560
7.553
7.546
7.541
7.538
7.532
7.260
5.676
5.675
5.673
5.510
5.507
3.598
3.586
3.575
3.007
2.995
2.984
1.696
1.418
PT
S
Br
E-3
8.5
8.0
7.5
7.0
6.5
6.0
5.5
5.0
4.5
4.0
3.5
3.0
2.5
2.0
1.5
1.0
ppm
4.803
0.998
0.991
2.000
2.005

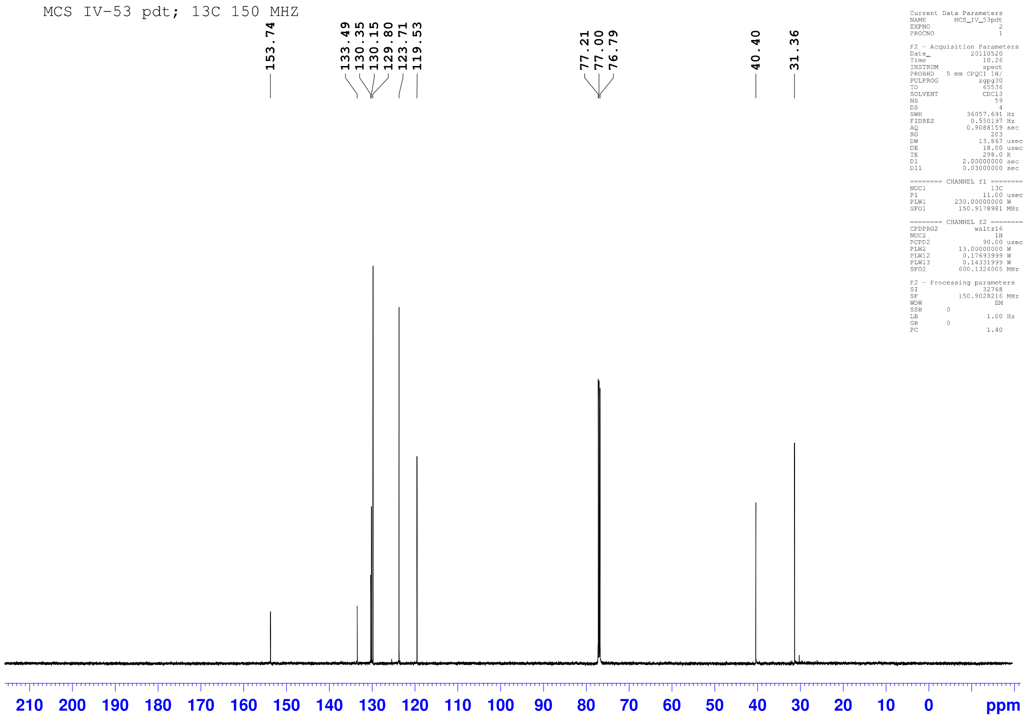
MCS IV-53 pdt; 13C 150 MHZ
153.74
133.49
130.35
130.15
129.80
123.71
119.53
77.21
77.00
76.79
40.40
31.36
210
200
190
180
170
160
150
140
130
120
110
100
90
80
70
60
50
40
30
20
10
0
ppm

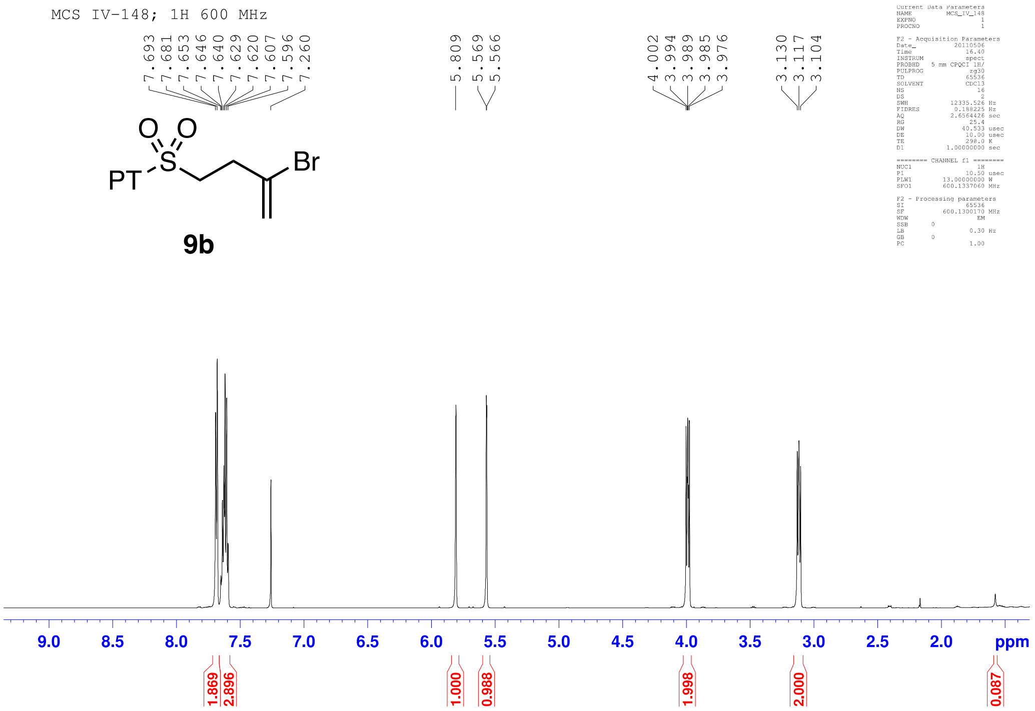
MCS IV-148; 1H 600 MHz
7.693 7.681 7.653 7.646 7.640 7.629 7.620 7.607 7.596 7.260
5.809 5.569 5.566
4.002 3.994 3.989 3.985 3.976
3.130 3.117 3.104
PT
S
O O
Br
9b
9.0 8.5 8.0 7.5 7.0 6.5 6.0 5.5 5.0 4.5 4.0 3.5 3.0 2.5 2.0 ppm
1.869 2.896 1.000 0.988 1.998 2.000 0.087

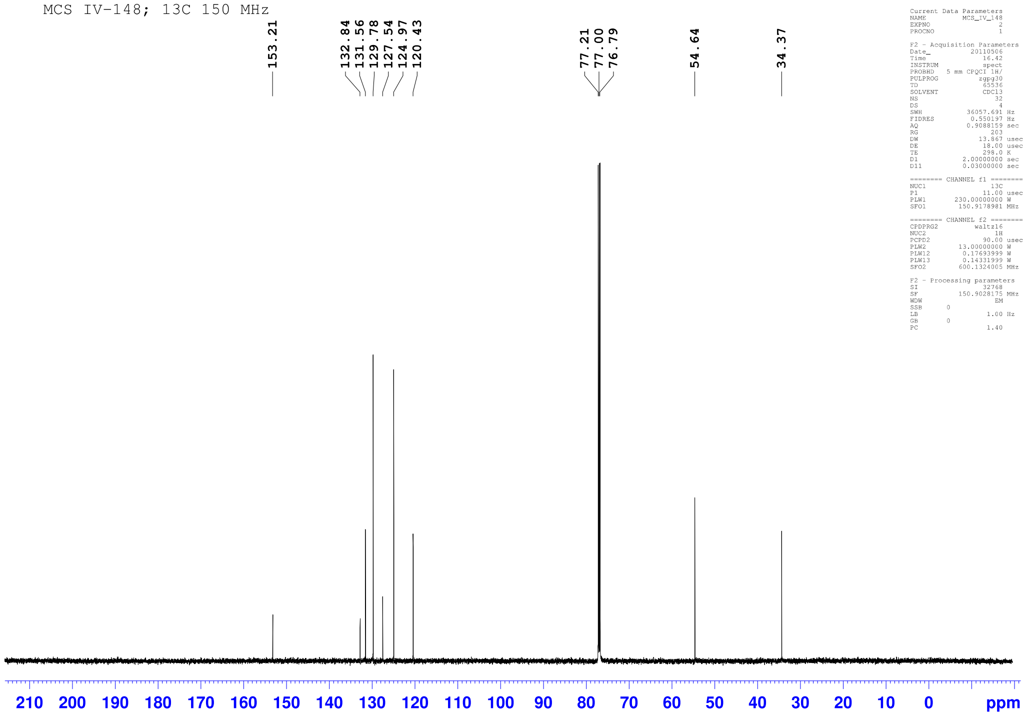
MCS IV-148; 13C 150 MHz
153.21
132.84 131.56 129.78 127.54 124.97 120.43
77.21 77.00 76.79
54.64
34.37
210 200 190 180 170 160 150 140 130 120 110 100 90 80 70 60 50 40 30 20 10 0 ppm

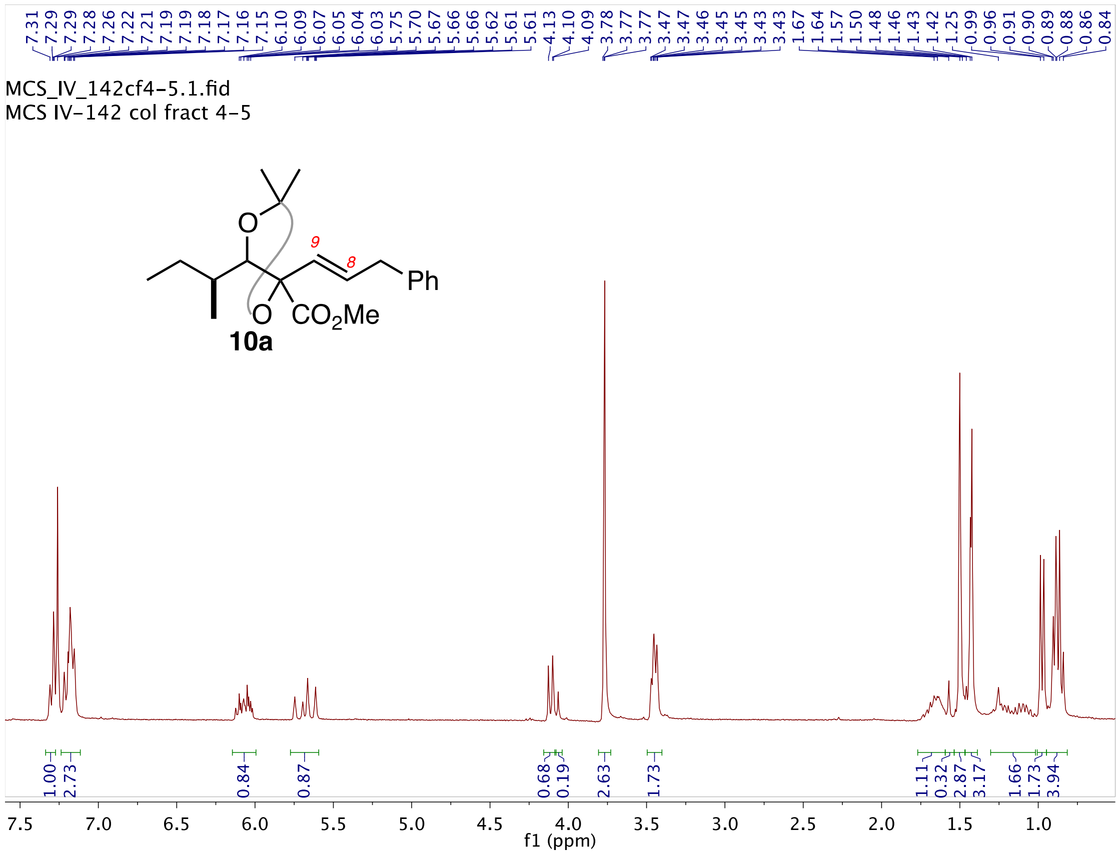
MCS_IV_142cf4-5.1.fid
MCS IV-142 col fract 4-5
10a
Ph
CO2Me
f1 (ppm)

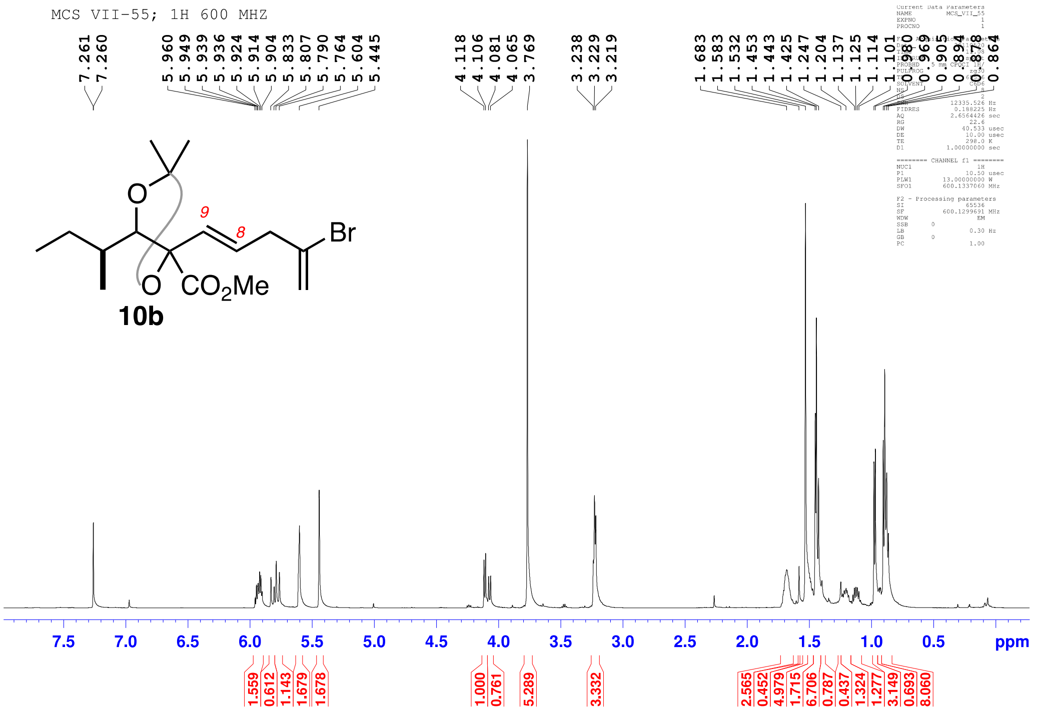
MCS VII-55; 1H 600 MHZ
7.261
7.260
5.960
5.949
5.939
5.936
5.924
5.914
5.904
5.833
5.807
5.790
5.764
5.604
5.445
4.118
4.106
4.081
4.065
3.769
3.238
3.229
3.219
1.683
1.583
1.532
1.453
1.443
1.425
1.247
1.204
1.137
1.125
1.114
1.101
0.980
0.969
0.905
0.894
0.878
0.864
9
8
O
O
Br
CO_2Me
10b
7.5
7.0
6.5
6.0
5.5
5.0
4.5
4.0
3.5
3.0
2.5
2.0
1.5
1.0
0.5
ppm
1.559
0.612
1.143
1.679
1.678
1.000
0.761
5.289
3.332
2.565
0.452
4.979
1.715
6.706
0.787
0.437
1.324
1.277
3.149
0.693
8.060

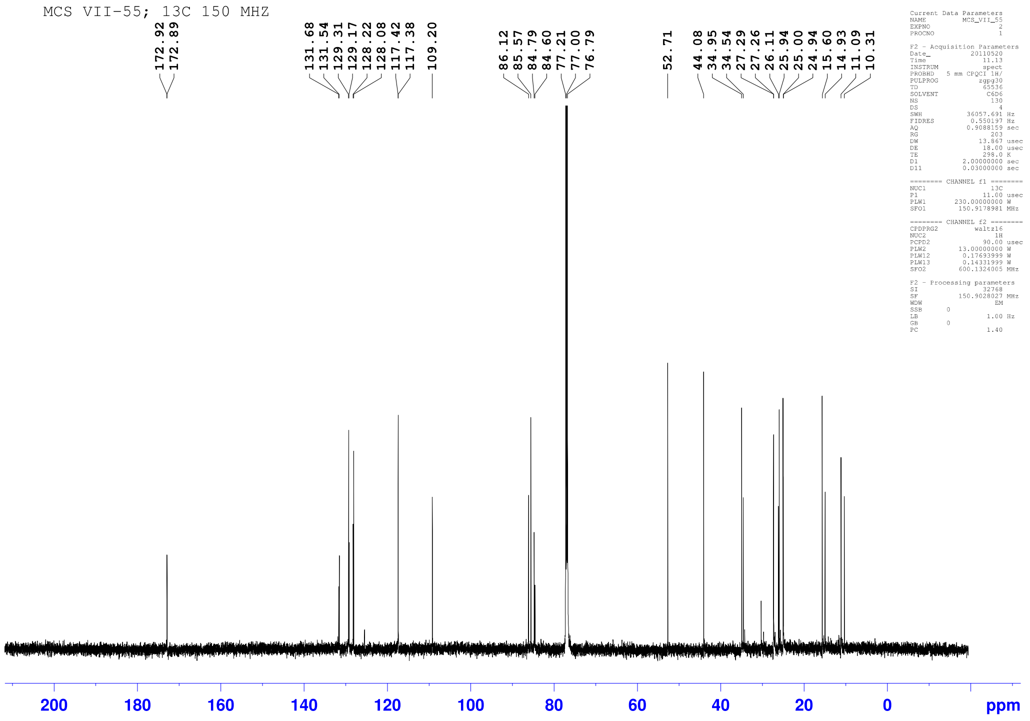
MCS VII-55; 13C 150 MHZ
172.92
172.89
131.68
131.54
129.31
129.17
128.22
128.08
117.42
117.38
109.20
86.12
85.57
84.79
84.60
77.21
77.00
76.79
52.71
44.08
34.95
34.54
27.29
27.26
26.11
25.94
25.00
24.94
15.60
14.93
11.09
10.31
200
180
160
140
120
100
80
60
40
20
0
ppm

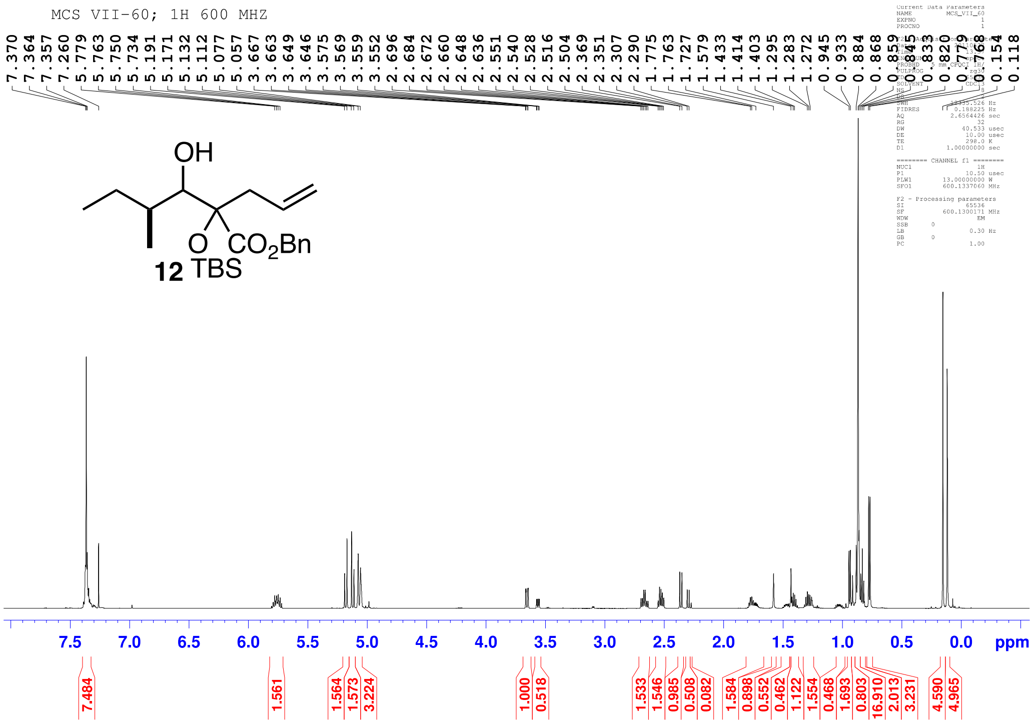
MCS VII-60; 1H 600 MHZ
7.370 7.364 7.357 7.260 5.779 5.763 5.750 5.734 5.191 5.171 5.132 5.112 5.077 5.057 3.667 3.663 3.649 3.646 3.575 3.569 3.559 3.552 2.696 2.684 2.672 2.660 2.648 2.636 2.551 2.540 2.528 2.516 2.504 2.369 2.351 2.307 2.290 1.775 1.763 1.727 1.579 1.433 1.414 1.403 1.295 1.283 1.272 0.945 0.933 0.884 0.868 0.859 0.845 0.833 0.820 0.779 0.768 0.154 0.118
OH
O
CO2Bn
12 TBS
7.5 7.0 6.5 6.0 5.5 5.0 4.5 4.0 3.5 3.0 2.5 2.0 1.5 1.0 0.5 0.0 ppm
7.484 1.561 1.564 1.573 3.224 1.000 0.518 1.533 1.546 0.985 0.508 0.082 1.584 0.898 0.552 0.462 1.122 1.554 0.468 1.693 0.803 16.910 2.013 3.231 4.590 4.965

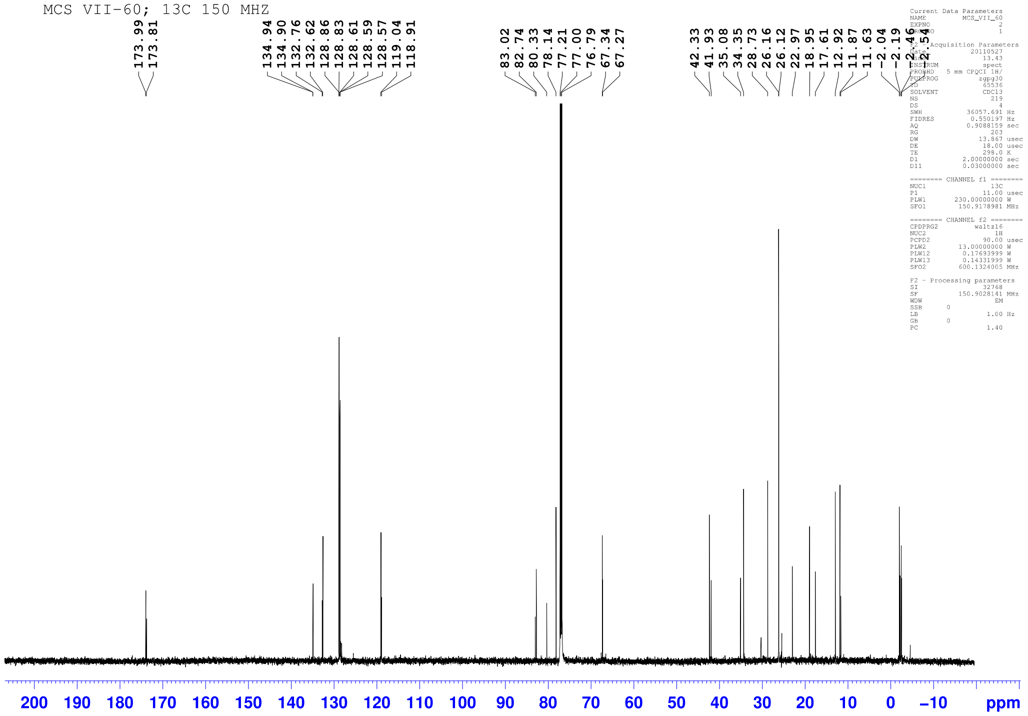
MCS VII-60; 13C 150 MHZ
173.99 173.81 134.94 134.90 132.76 132.62 128.86 128.83 128.61 128.59 128.57 119.04 118.91 83.02 82.74 80.33 78.14 77.21 77.00 76.79 67.34 67.27 42.33 41.93 35.08 34.35 28.73 26.16 26.12 22.97 18.95 17.61 12.92 11.87 11.63 -2.04 -2.19 -2.46 -2.54
200 190 180 170 160 150 140 130 120 110 100 90 80 70 60 50 40 30 20 10 0 -10 ppm

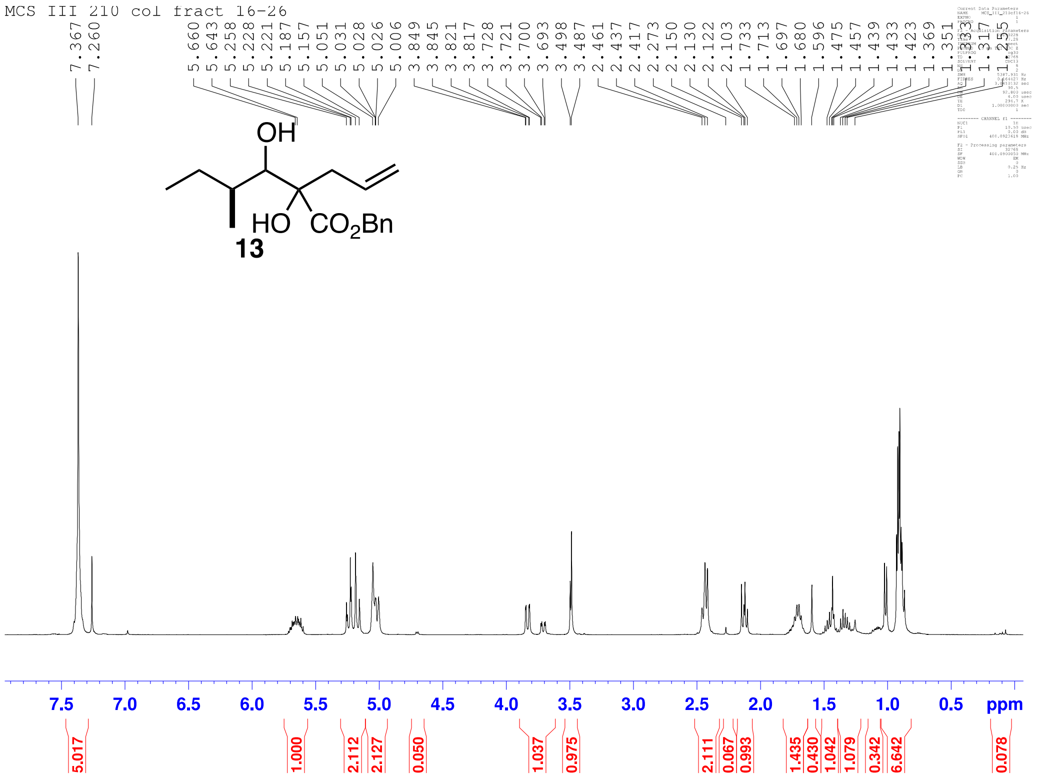

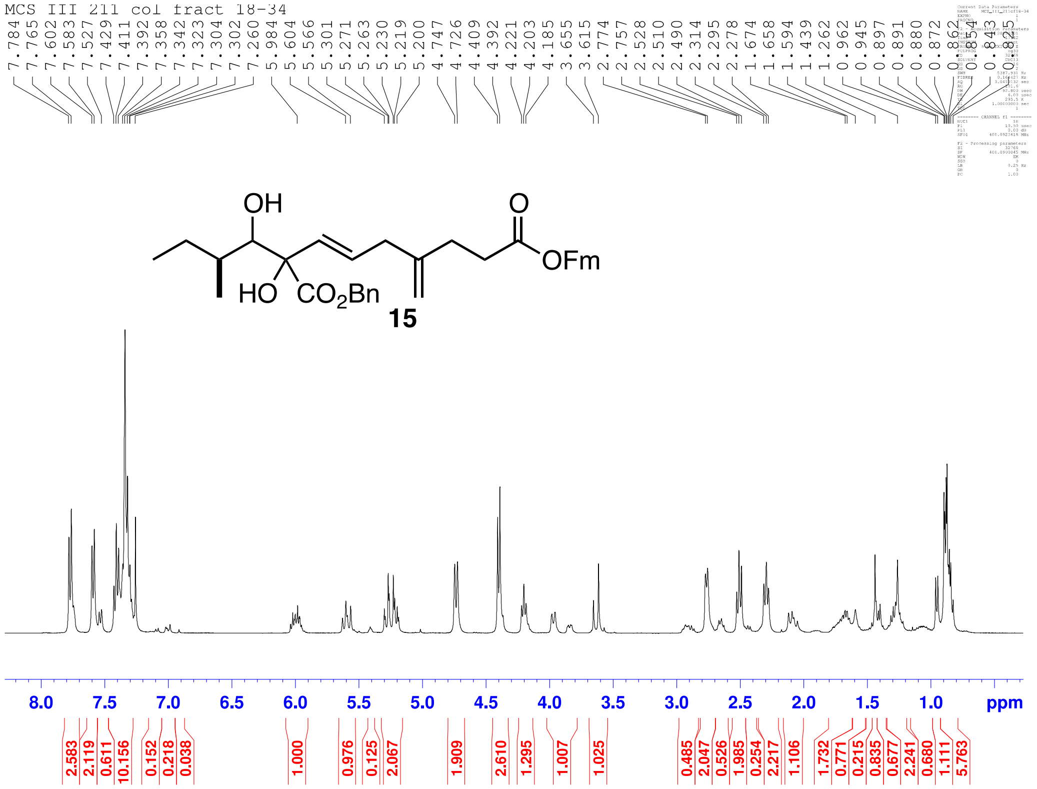

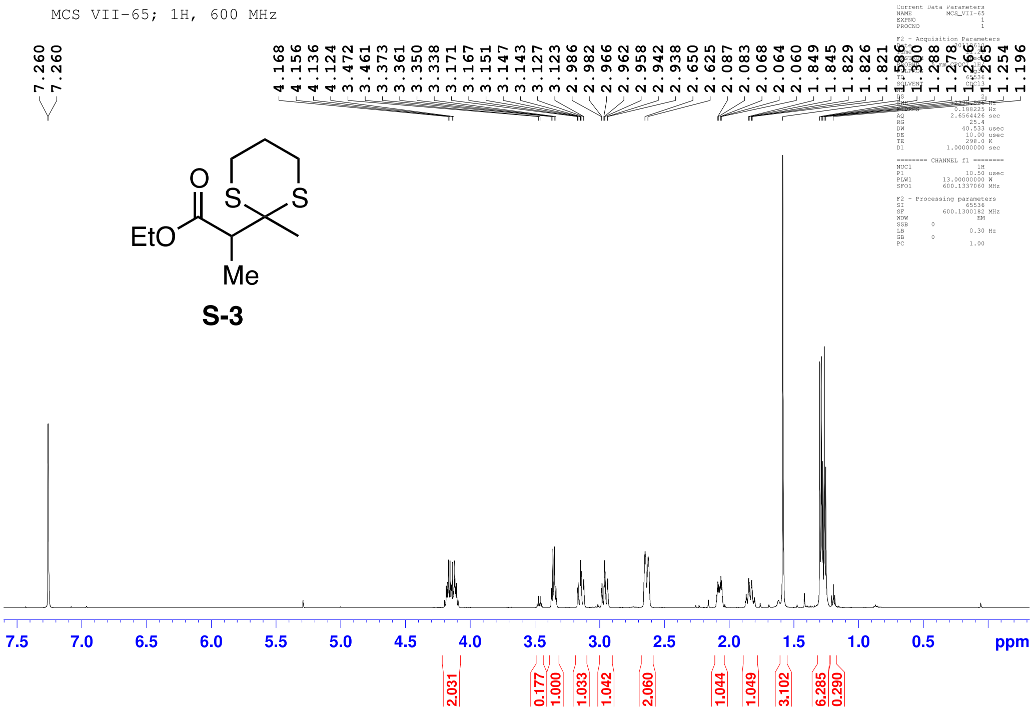
MCS VII-65; 1H, 600 MHz
7.260
7.260
4.168
4.156
4.136
4.124
3.472
3.461
3.373
3.361
3.350
3.338
3.171
3.167
3.151
3.147
3.143
3.127
3.123
2.986
2.982
2.966
2.962
2.958
2.942
2.938
2.650
2.625
2.087
2.083
2.068
2.064
2.060
1.849
1.845
1.829
1.826
1.821
1.586
1.300
1.288
1.278
1.266
1.263
1.254
1.196
EtO
O
S
S
Me
S-3
7.5 7.0 6.5 6.0 5.5 5.0 4.5 4.0 3.5 3.0 2.5 2.0 1.5 1.0 0.5 ppm
2.031
0.177
1.000
1.033
1.042
2.060
1.044
1.049
3.102
6.285
0.290

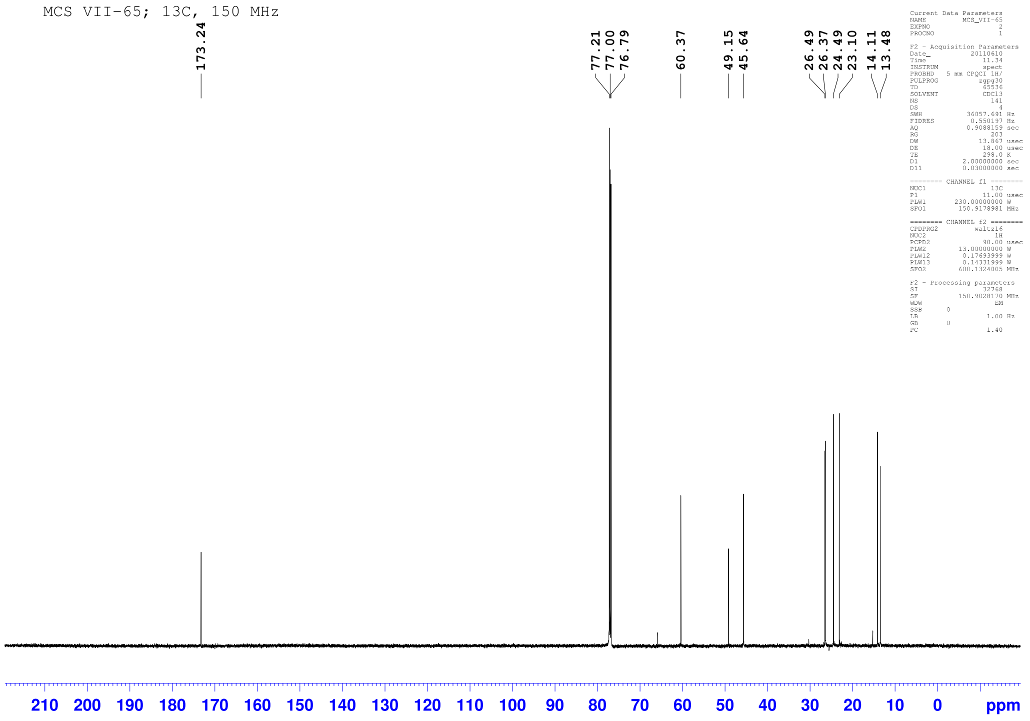
MCS VII-65; 13C, 150 MHz
173.24
77.21
77.00
76.79
60.37
49.15
45.64
26.49
26.37
24.49
23.10
14.11
13.48
210 200 190 180 170 160 150 140 130 120 110 100 90 80 70 60 50 40 30 20 10 0 ppm

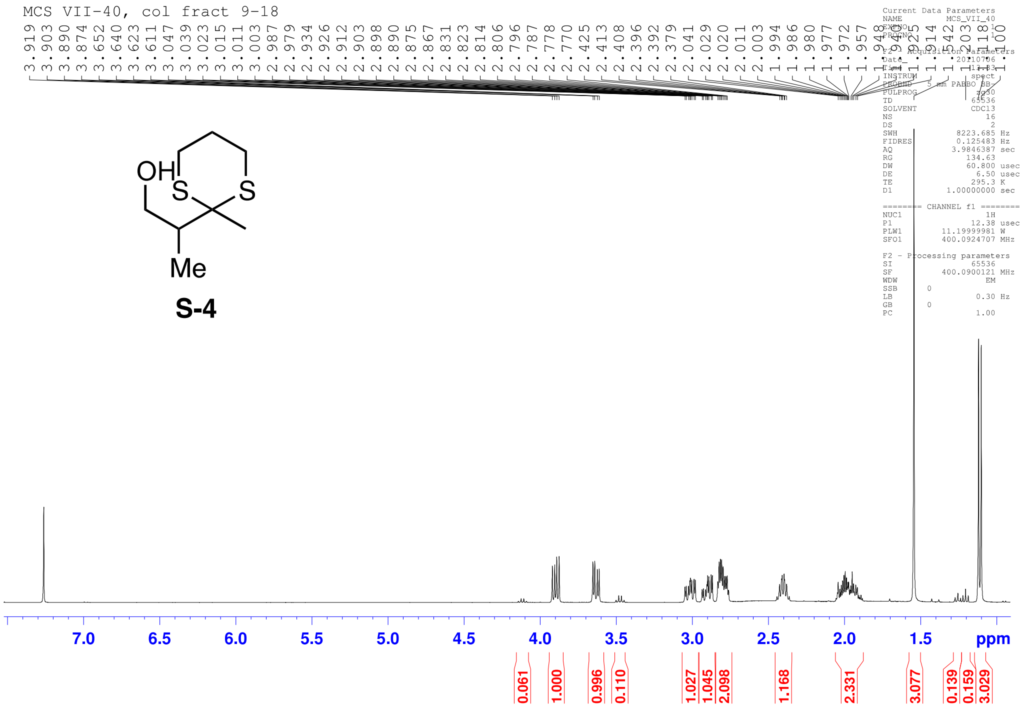
MCS VII-40, col fract 9-18
OH
S
S
Me
S-4
7.0 6.5 6.0 5.5 5.0 4.5 4.0 3.5 3.0 2.5 2.0 1.5 ppm
0.061 1.000 0.996 0.110 1.027 1.045 2.098 1.168 2.331 3.077 0.139 0.159 3.029

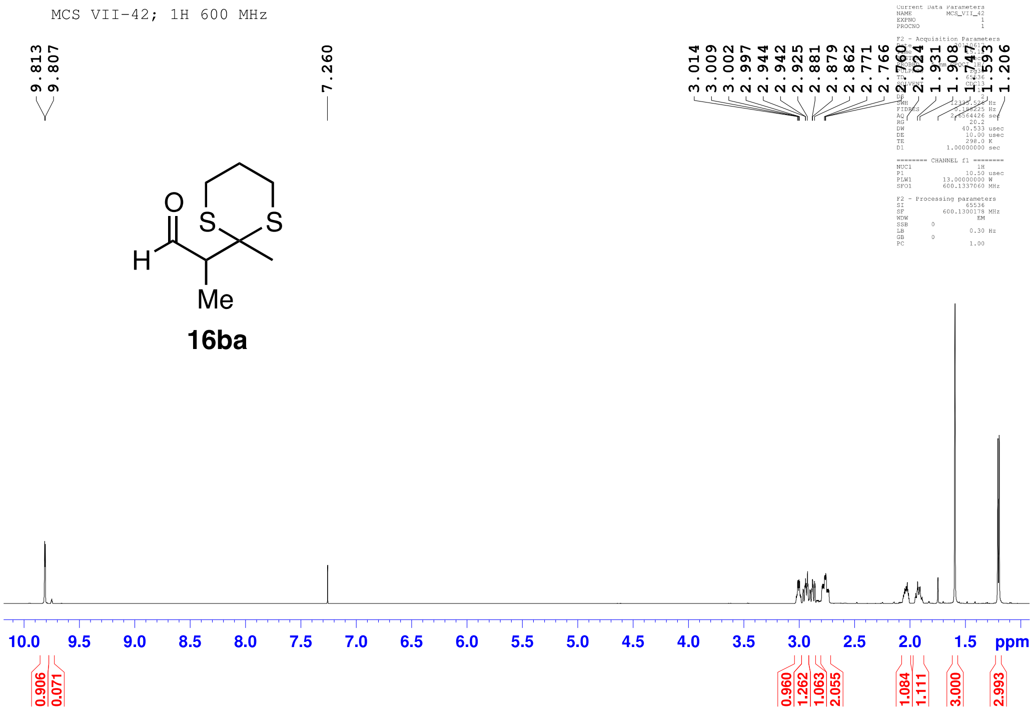
MCS VII-42; 1H 600 MHz
9.813
9.807
7.260
3.014
3.009
3.002
2.997
2.944
2.942
2.925
2.881
2.879
2.862
2.771
2.766
2.760
2.024
1.931
1.908
1.747
1.593
1.206
O
S
S
H
Me
16ba
10.0 9.5 9.0 8.5 8.0 7.5 7.0 6.5 6.0 5.5 5.0 4.5 4.0 3.5 3.0 2.5 2.0 1.5 ppm
0.906
0.071
0.960
1.262
1.063
2.055
1.084
1.111
3.000
2.993

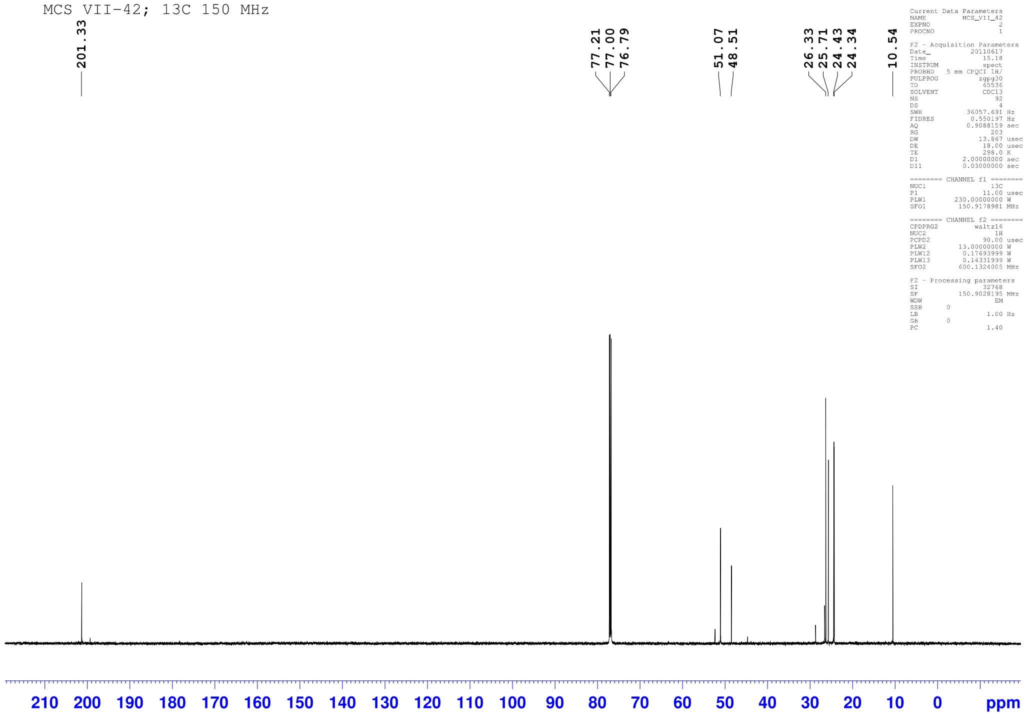
MCS VII-42; 13C 150 MHz
201.33
77.21
77.00
76.79
51.07
48.51
26.33
25.71
24.43
24.34
10.54
210 200 190 180 170 160 150 140 130 120 110 100 90 80 70 60 50 40 30 20 10 0 ppm

MCS VII-45; 1H 600 MHz

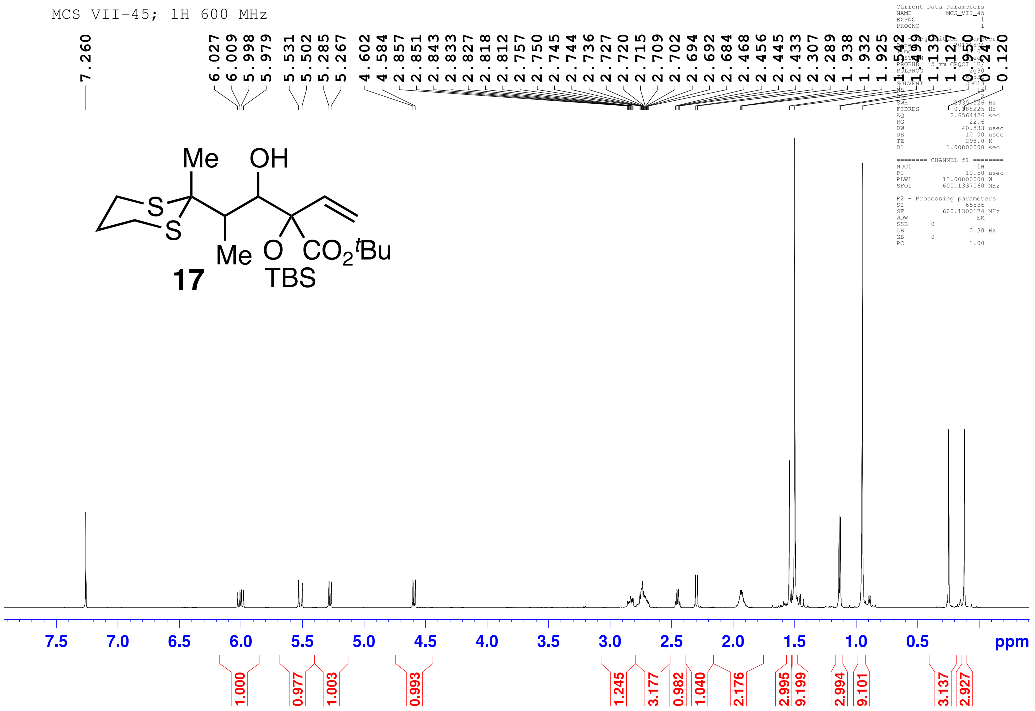

MCS VII-45; 13C 150 MHz

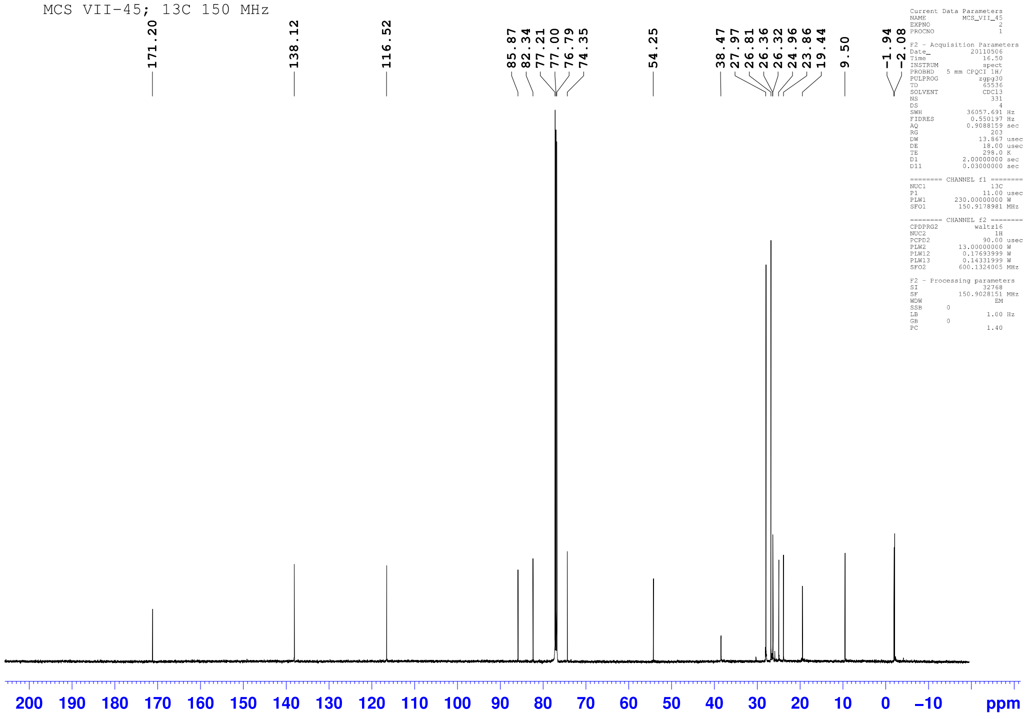

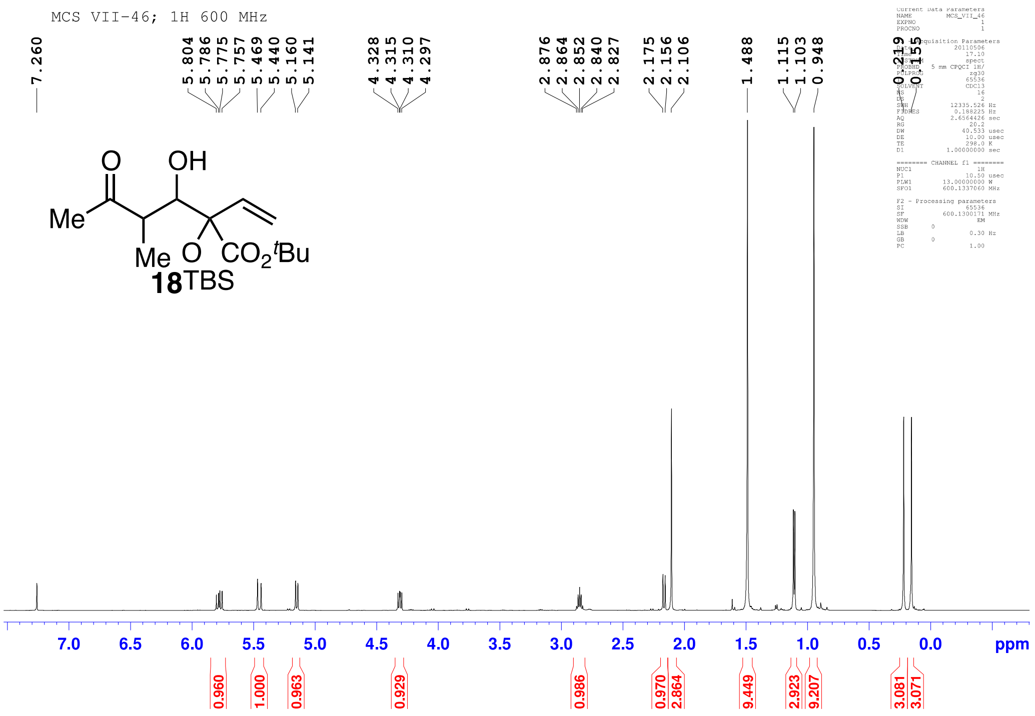
MCS VII-46; 1H 600 MHz
7.260
5.804 5.786 5.775 5.757 5.469 5.440 5.160 5.141
4.328 4.315 4.310 4.297
2.876 2.864 2.852 2.840 2.827
2.175 2.156 2.106
1.488
1.115 1.103 0.948
0.219 0.155
Me O OH Me O CO2tBu TBS
18
ppm
0.960 1.000 0.963 0.929 0.986 0.970 2.864 9.449 2.923 9.207 3.081 3.071

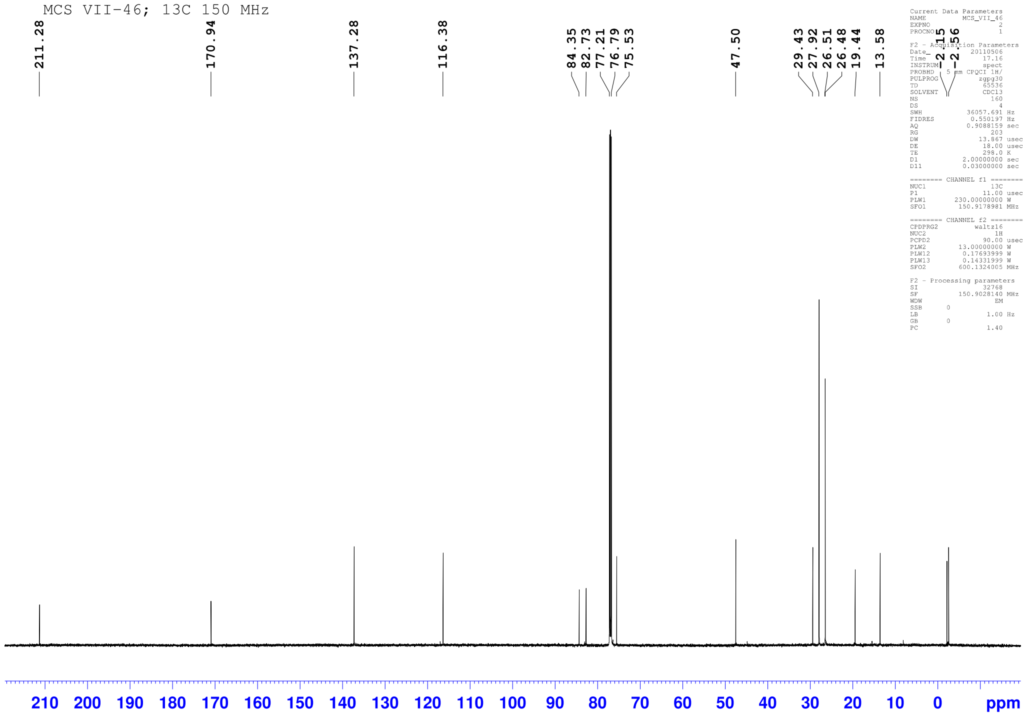
MCS VII-46; 13C 150 MHz
211.28
170.94
137.28
116.38
84.35 82.73 77.21 76.79 75.53
47.50
29.43 27.92 26.51 26.48 19.44 13.58
-2.15 -2.56
ppm

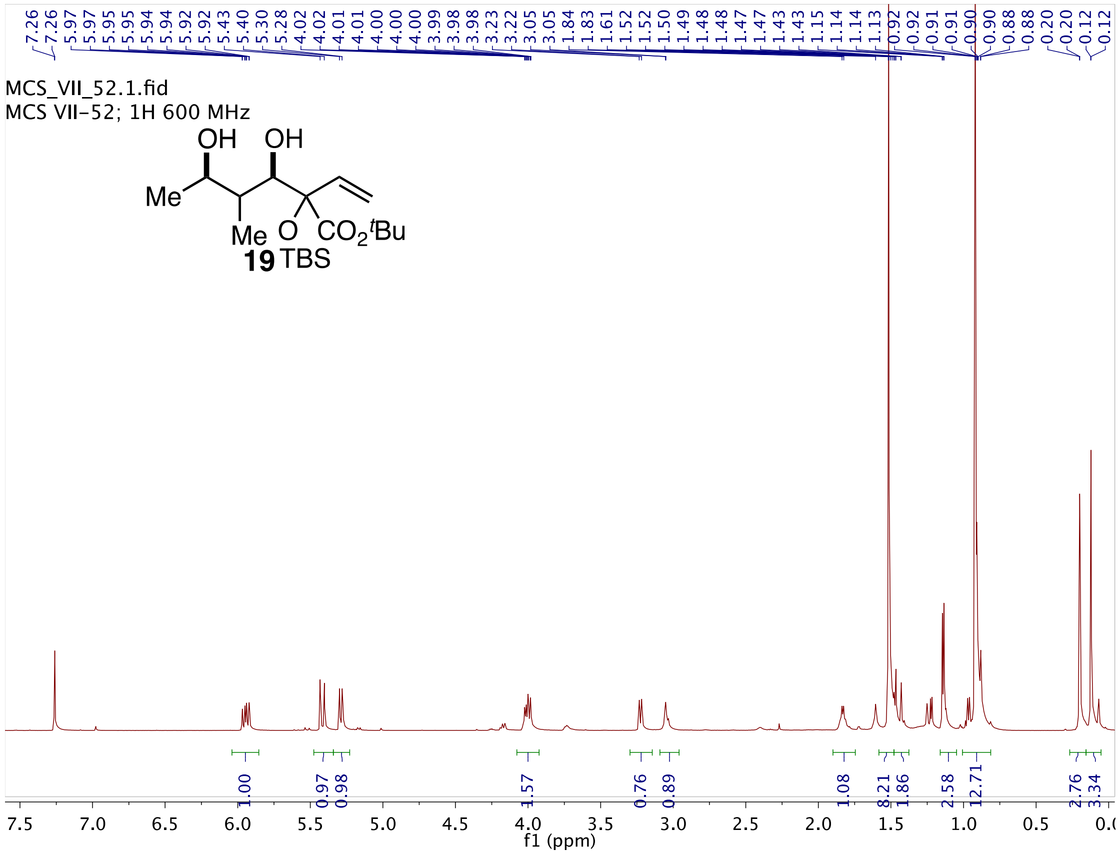
MCS_VII_52.1.fid
MCS VII-52; 1H 600 MHz
19
f1 (ppm)

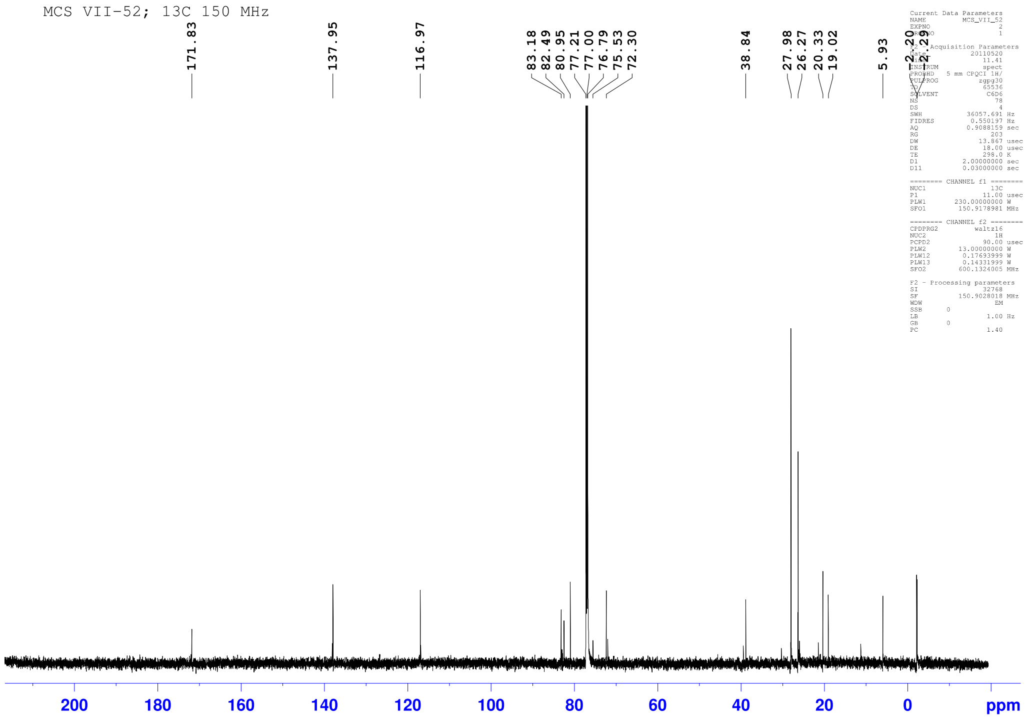
MCS VII-52; 13C 150 MHz
171.83
137.95
116.97
83.18
82.49
80.95
77.21
77.00
76.79
75.53
72.30
38.84
27.98
26.27
20.33
19.02
5.93
ppm

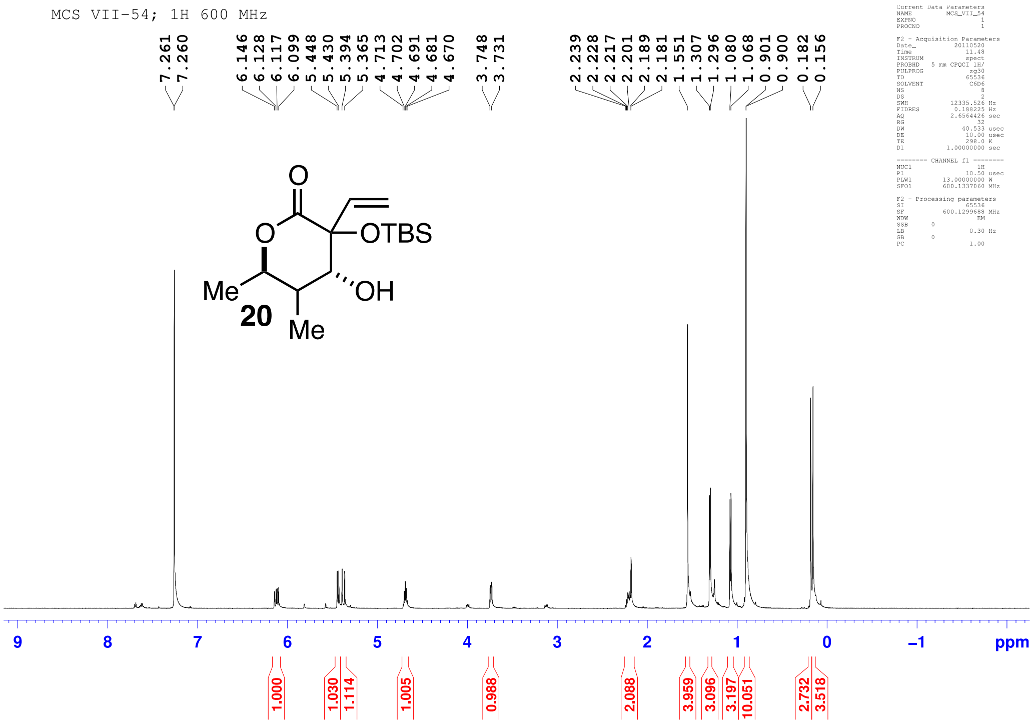
MCS VII-54; 1H 600 MHz
7.261
7.260
6.146
6.128
6.117
6.099
5.448
5.430
5.394
5.365
4.713
4.702
4.691
4.681
4.670
3.748
3.731
2.239
2.228
2.217
2.201
2.189
2.181
1.551
1.307
1.296
1.080
1.068
0.901
0.900
0.182
0.156
O
OTBS
O
Me
OH
20
Me
9
8
7
6
5
4
3
2
1
0
–1
ppm
1.000
1.030
1.114
1.005
0.988
2.088
3.959
3.096
3.197
10.051
2.732
3.518

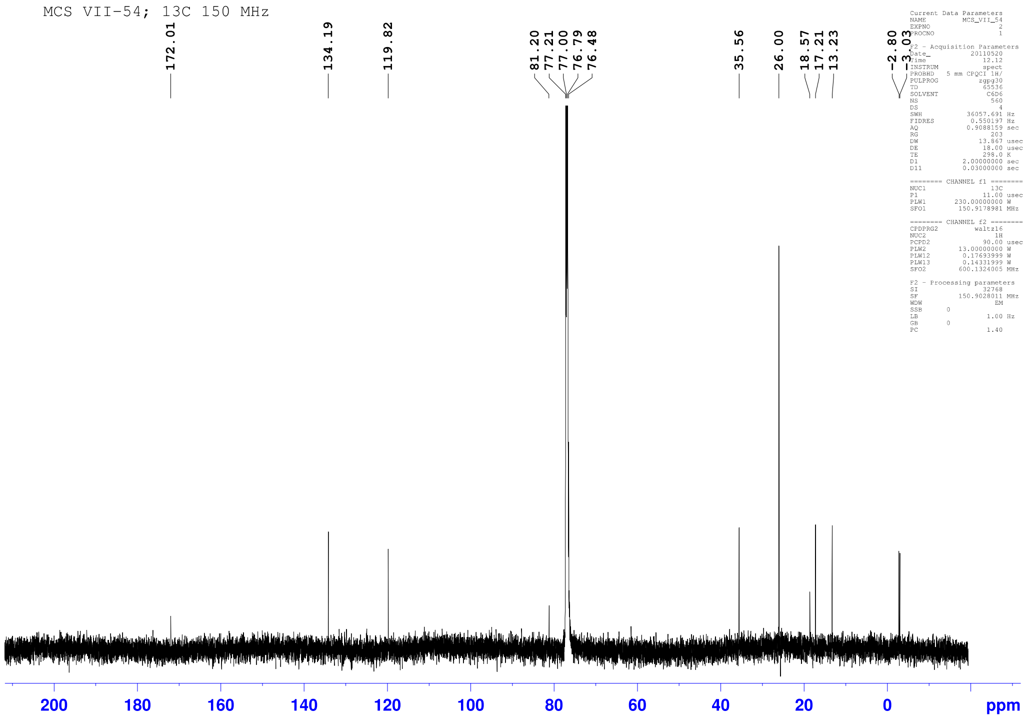
MCS VII-54; 13C 150 MHz
172.01
134.19
119.82
81.20
77.21
77.00
76.79
76.48
35.56
26.00
18.57
17.21
13.23
-2.80
-3.03
200
180
160
140
120
100
80
60
40
20
0
ppm

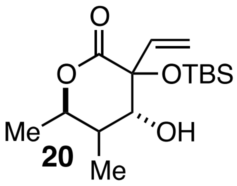

MCS VII-54; 600 MHz NOESY

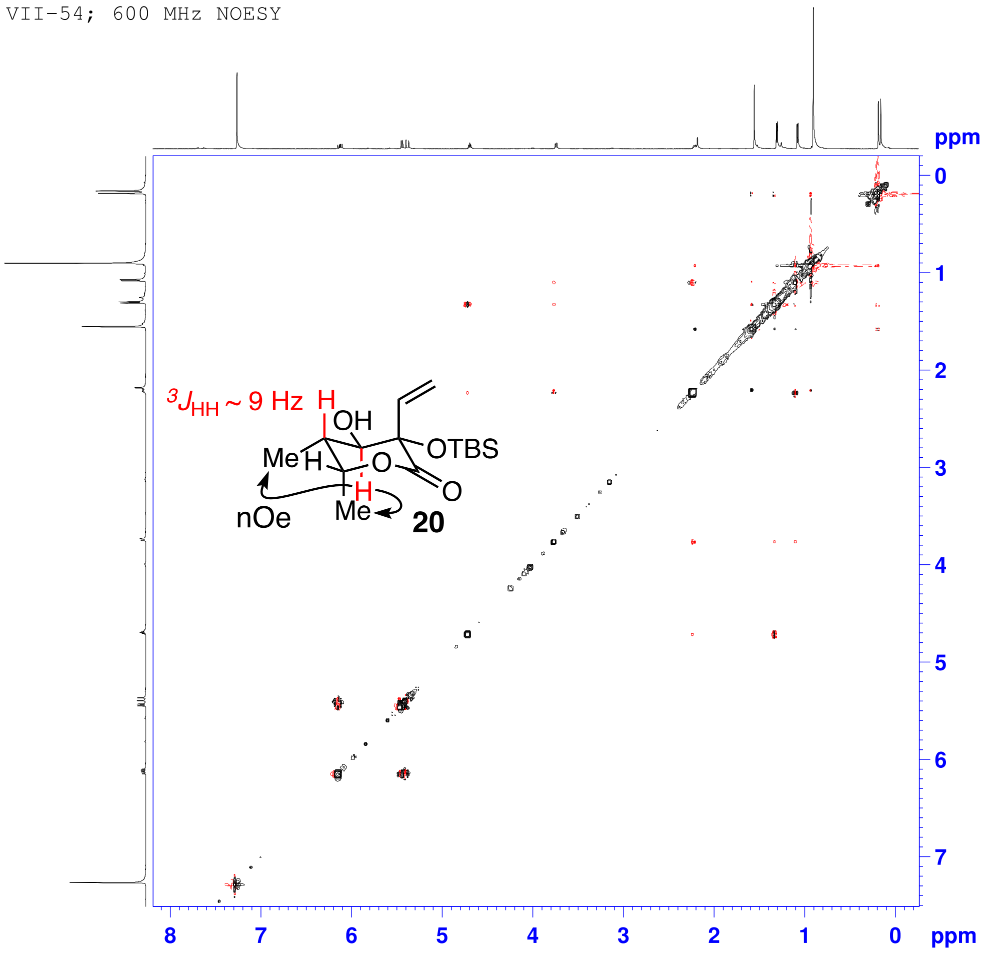


```
Current Data Parameters
NAME          MCS_VII_54
EXPNO                  3
PROCNO                 1

F2 - Acquisition Parameters
Date_           20110520
Time               15.07
INSTRUM            spect
PROBHD   5 mm CPQCI 1H/
PULPROG      noesygpphpp
TD                  2048
SOLVENT            CDCl3
NS                     4
DS                    32
SWH             6127.451 Hz
FIDRES          2.991920 Hz
AQ             0.1671668 sec
RG                   203
DW                81.600 usec
DE                 10.00 usec
TE                 298.0 K
D0            0.00006823 sec
D1            2.00000000 sec
D8            0.30000001 sec
D11           0.03000000 sec
D12           0.00002000 sec
D16           0.00020000 sec
IN0           0.00016320 sec

======== CHANNEL f1 ========
NUC1                  1H
P1                 10.50 usec
P2                 21.00 usec
P17              2500.00 usec
PLW1         13.00000000 W
PLW10         2.12019992 W
SFO1         600.1327625 MHz

====== GRADIENT CHANNEL =====
GPNAM1        SMSQ10.100
GPZ1               40.00 %
P16              1000.00 usec

F1 - Acquisition parameters
TD                   256
SFO1            600.1328 MHz
FIDRES         23.935356 Hz
SW                10.210 ppm
FnMODE       States-TPPI

F2 - Processing parameters
SI                  1024
SF           600.1300000 MHz
WDW                QSINE
SSB                    2
LB        0 Hz
GB        0
PC                  1.00

F1 - Processing parameters
SI                  1024
MC2          States-TPPI
SF           600.1300000 MHz
WDW
SSB                    2
LB        0 Hz
GB        0
```

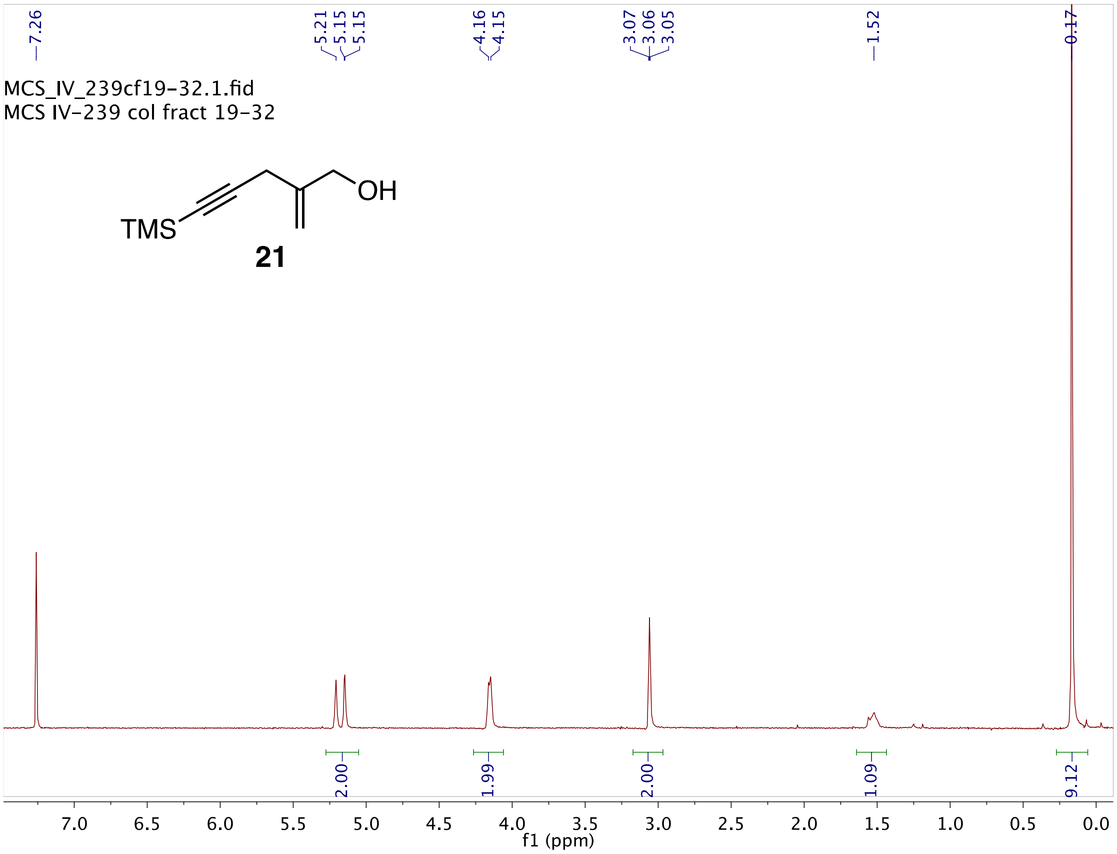

7.26
5.21
5.15
5.15
4.16
4.15
3.07
3.06
3.05
1.52
0.17
MCS_IV_239cf19-32.1.fid
MCS IV-239 col fract 19-32
TMS
OH
21
2.00
1.99
2.00
1.09
9.12
7.0 6.5 6.0 5.5 5.0 4.5 4.0 3.5 3.0 2.5 2.0 1.5 1.0 0.5 0.0
f1 (ppm)

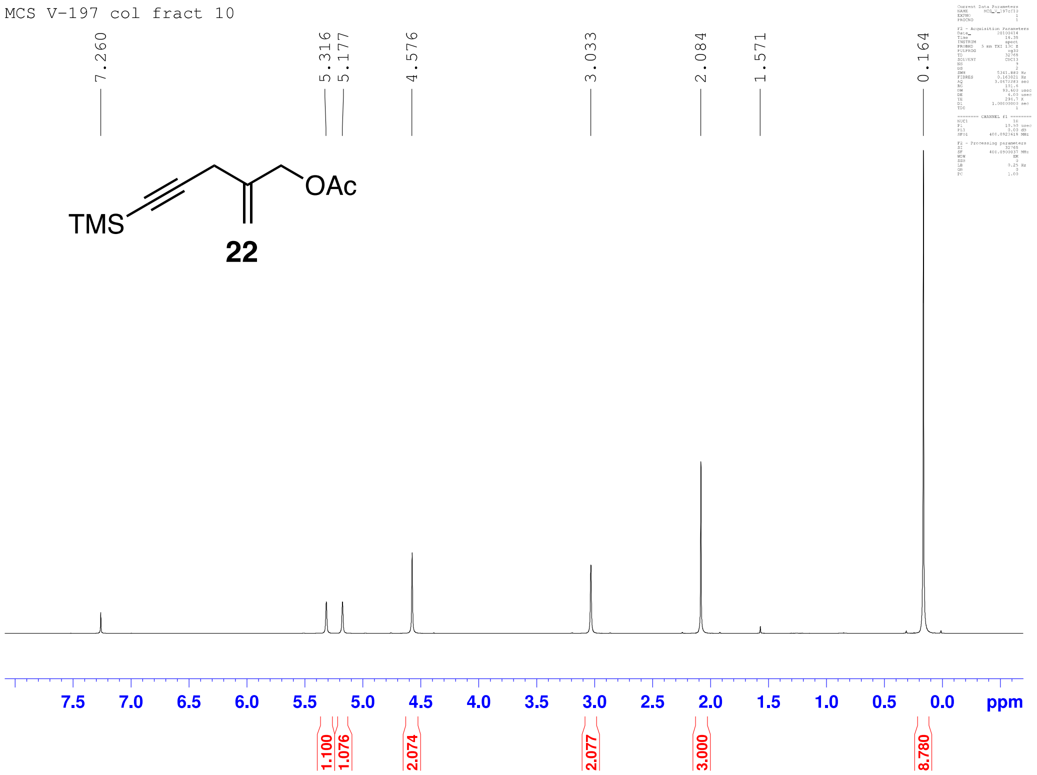
MCS V-197 col fract 10
7.260
5.316
5.177
4.576
3.033
2.084
1.571
0.164
TMS
OAc
22
7.5 7.0 6.5 6.0 5.5 5.0 4.5 4.0 3.5 3.0 2.5 2.0 1.5 1.0 0.5 0.0 ppm
1.100
1.076
2.074
2.077
3.000
8.780

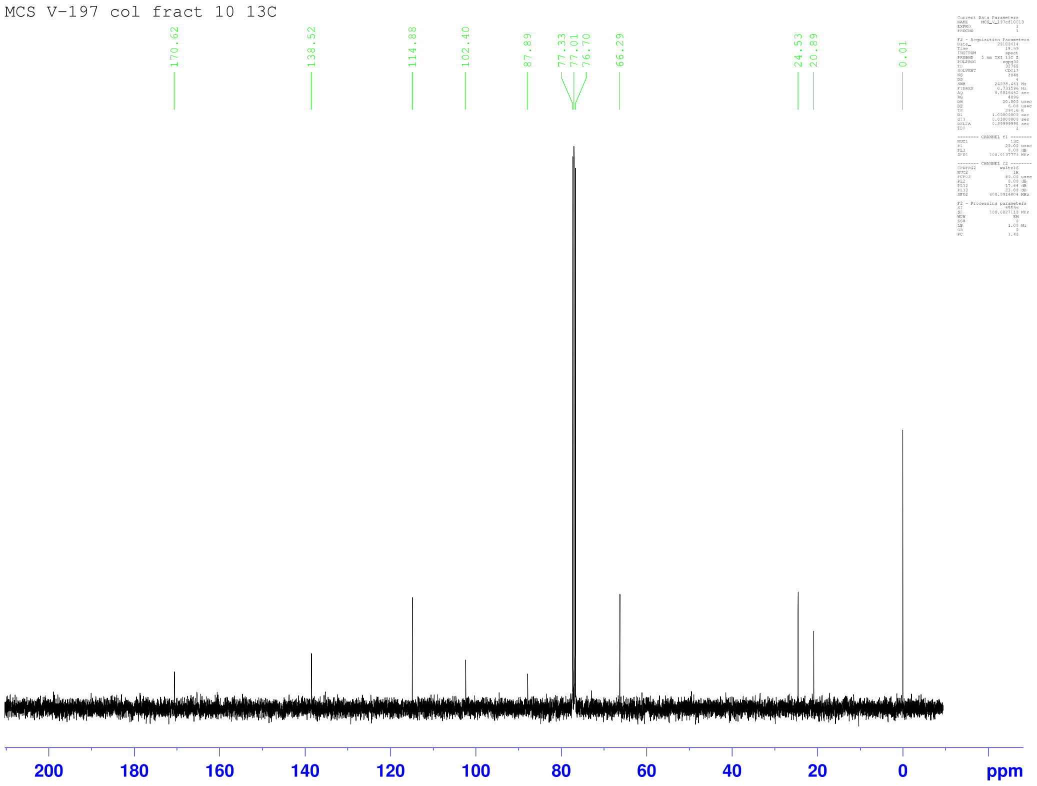
MCS V-197 col fract 10 13C
170.62
138.52
114.88
102.40
87.89
77.33
77.01
76.70
66.29
24.53
20.89
0.01
200 180 160 140 120 100 80 60 40 20 0 ppm

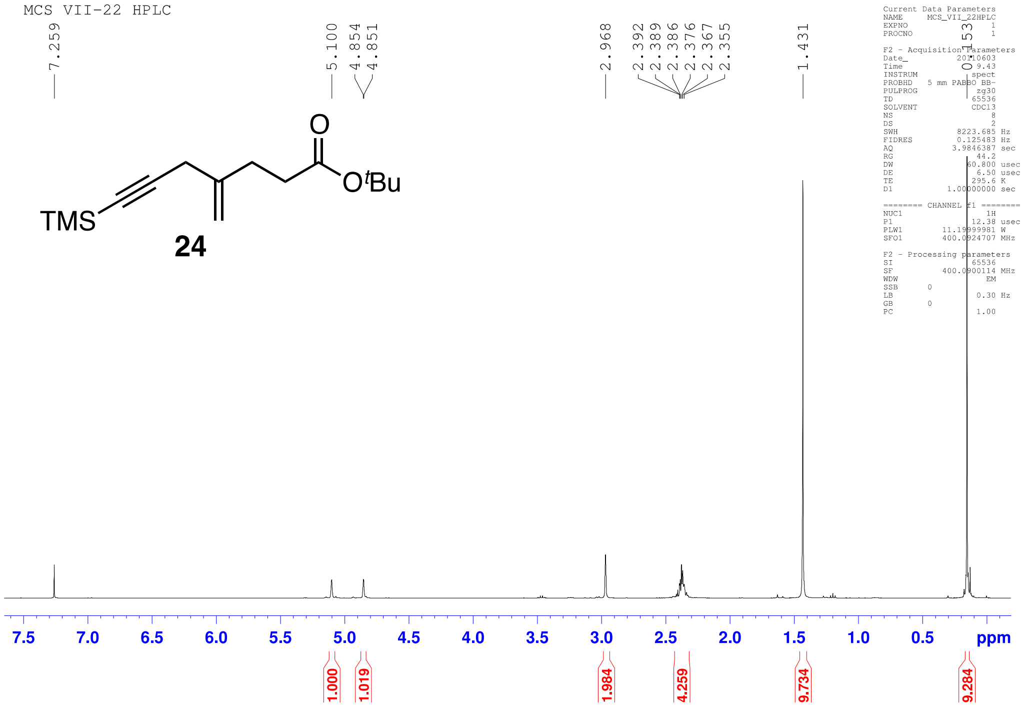
MCS VII-22 HPLC
7.259
5.100
4.854
4.851
2.968
2.392
2.389
2.386
2.376
2.367
2.355
1.431
0.153
O
O^tBu
TMS
24
7.5 7.0 6.5 6.0 5.5 5.0 4.5 4.0 3.5 3.0 2.5 2.0 1.5 1.0 0.5 ppm
1.000
1.019
1.984
4.259
9.734
9.284

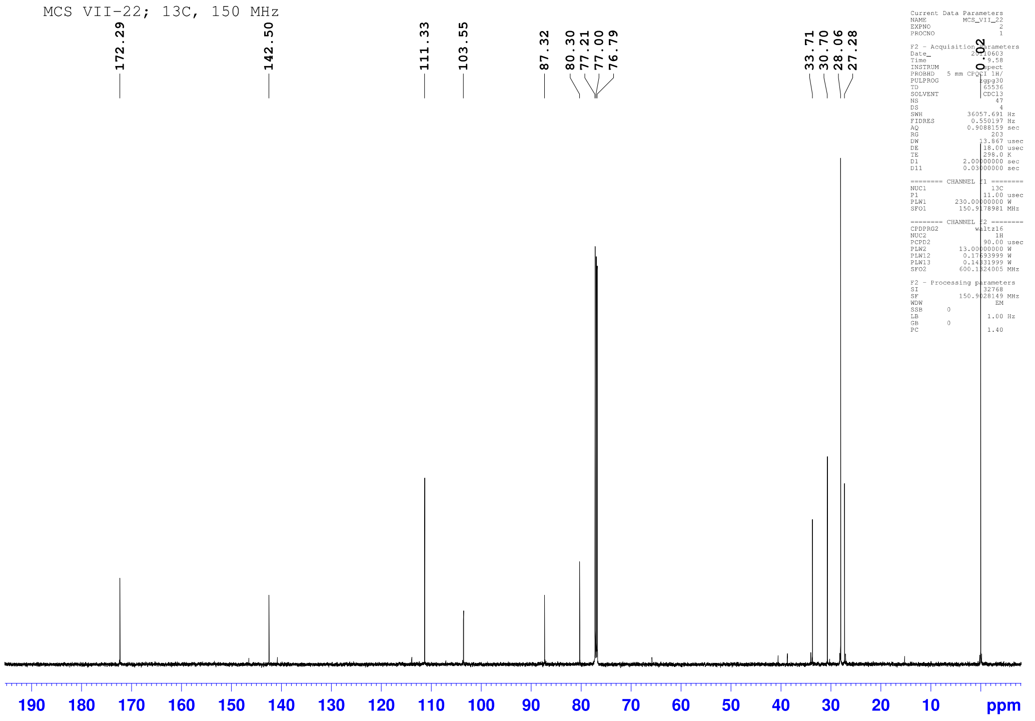
MCS VII-22; 13C, 150 MHz
172.29
142.50
111.33
103.55
87.32
80.30
77.21
77.00
76.79
33.71
30.70
28.06
27.28
0.02
190 180 170 160 150 140 130 120 110 100 90 80 70 60 50 40 30 20 10 ppm

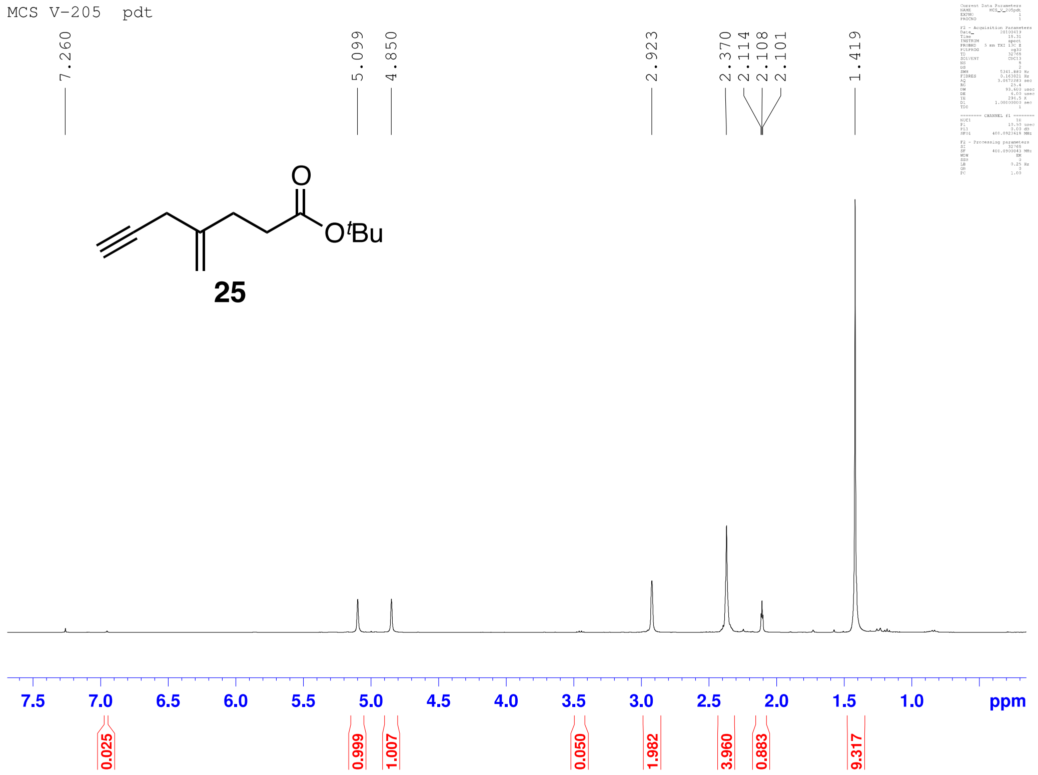
MCS V-205 pdt
7.260
5.099
4.850
2.923
2.370
2.114
2.108
2.101
1.419
O
OtBu
25
7.5
7.0
6.5
6.0
5.5
5.0
4.5
4.0
3.5
3.0
2.5
2.0
1.5
1.0
ppm
0.025
0.999
1.007
0.050
1.982
3.960
0.883
9.317

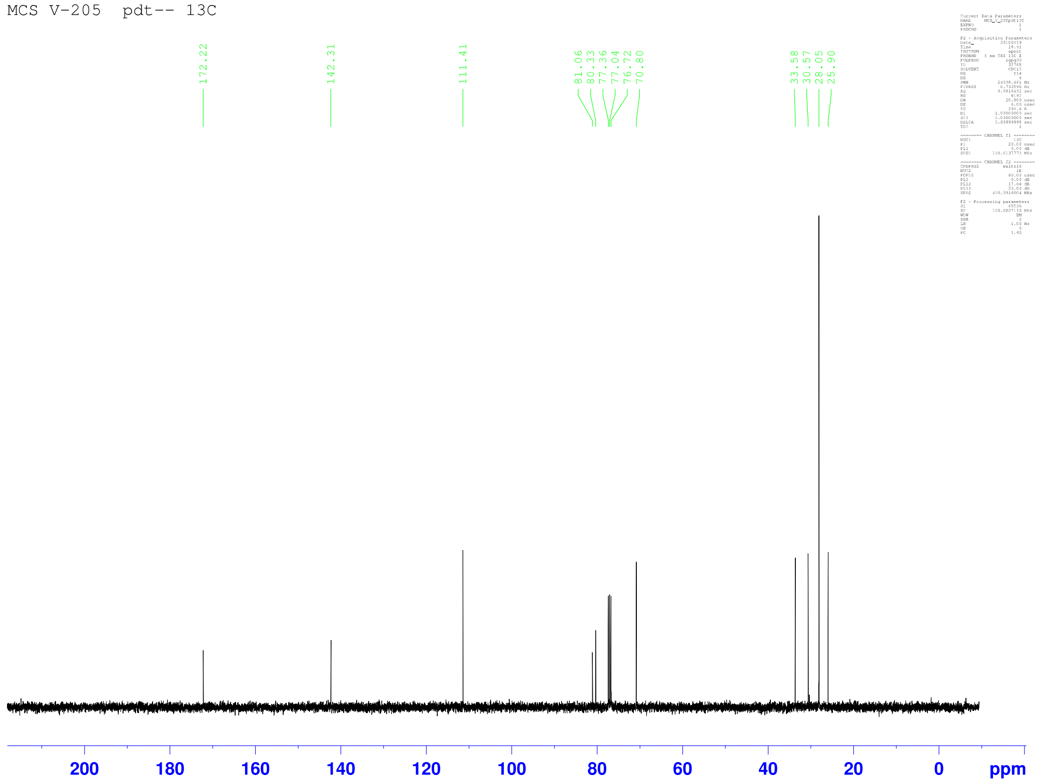
MCS V-205 pdt-- 13C
172.22
142.31
111.41
81.06
80.33
77.36
77.04
76.72
70.80
33.58
30.57
28.05
25.90
200
180
160
140
120
100
80
60
40
20
0
ppm

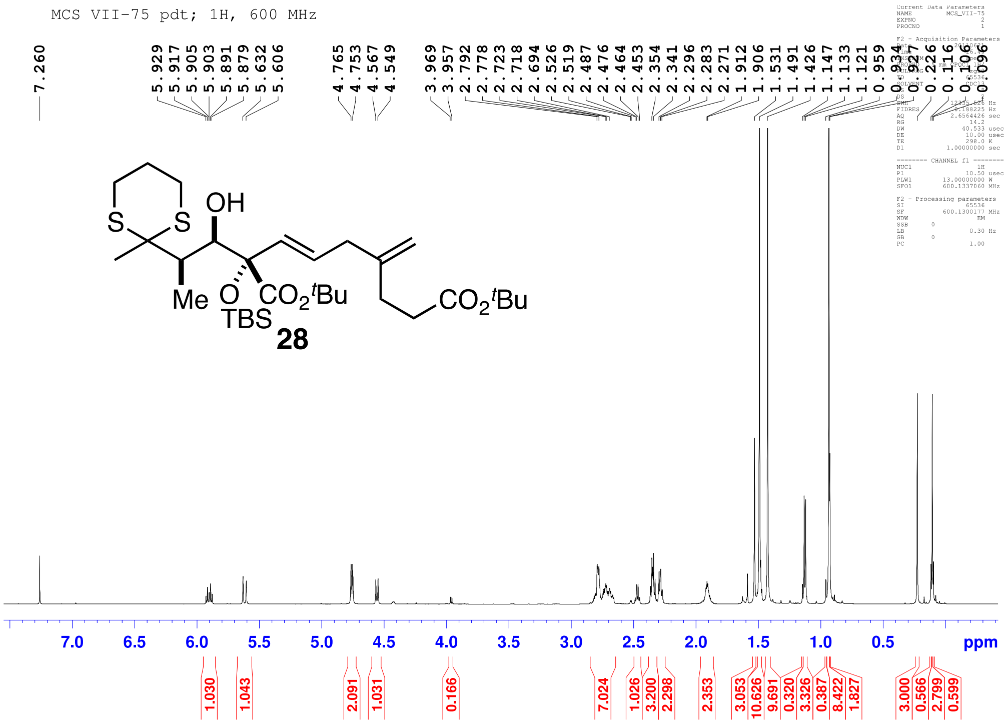
MCS VII-75 pdt; 1H, 600 MHz
7.260
5.929 5.917 5.905 5.903 5.891 5.879 5.632 5.606
4.765 4.753 4.567 4.549
3.969 3.957 2.792 2.778 2.723 2.718 2.694 2.526 2.519 2.487 2.476 2.464 2.453 2.354 2.341 2.296 2.283 2.271 1.912 1.906 1.531 1.491 1.426 1.147 1.133 1.121 0.959 0.934 0.927 0.226 0.116 0.106 0.096
OH
S S
Me O CO2tBu
TBS
CO2tBu
28
7.0 6.5 6.0 5.5 5.0 4.5 4.0 3.5 3.0 2.5 2.0 1.5 1.0 0.5 ppm
1.030 1.043 2.091 1.031 0.166 7.024 1.026 3.200 2.298 2.353 3.053 10.626 9.691 0.320 3.326 0.387 8.422 1.827 3.000 0.566 2.799 0.599

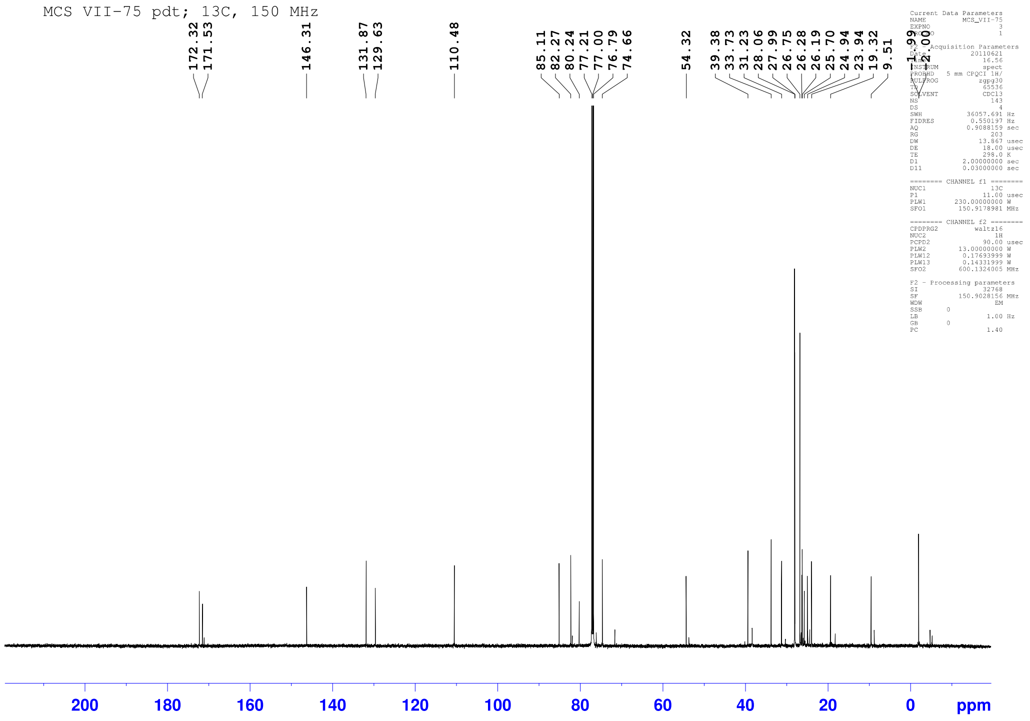
MCS VII-75 pdt; 13C, 150 MHz
172.32 171.53 146.31 131.87 129.63 110.48
85.11 82.27 80.24 77.21 77.00 76.79 74.66
54.32 39.38 33.73 31.23 28.06 27.99 26.75 26.28 26.19 25.70 24.94 23.94 19.32 9.51
200 180 160 140 120 100 80 60 40 20 0 ppm

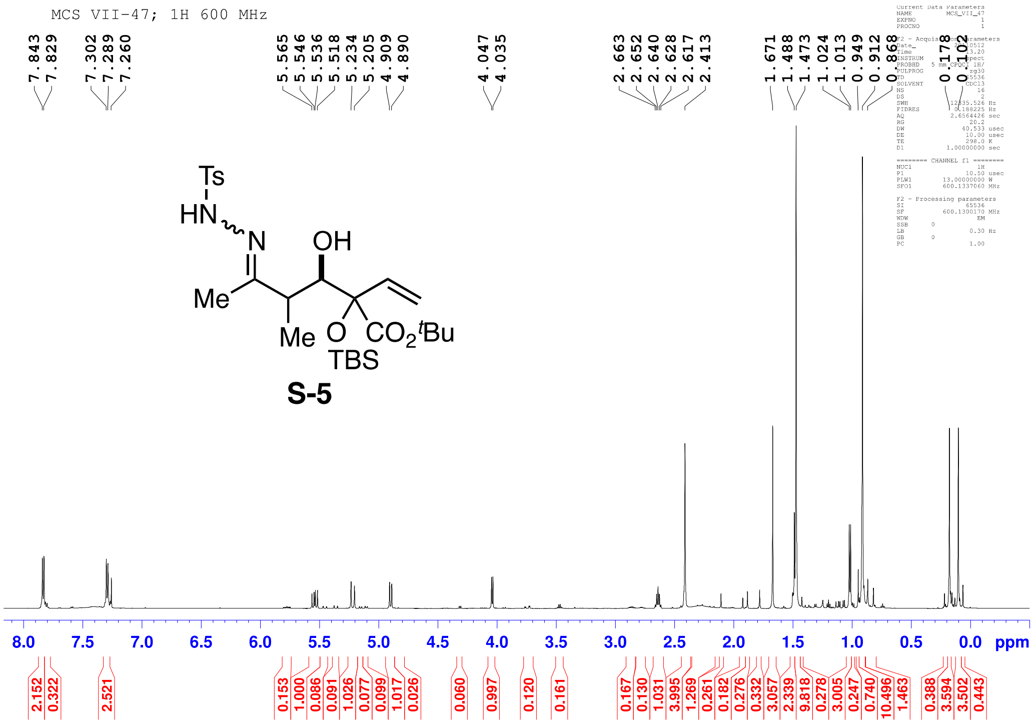
MCS VII-47; 1H 600 MHz
7.843
7.829
7.302
7.289
7.260
5.565
5.546
5.536
5.518
5.234
5.205
4.909
4.890
4.047
4.035
2.663
2.652
2.640
2.628
2.617
2.413
1.671
1.488
1.473
1.024
1.013
0.949
0.912
0.868
0.178
0.102
Ts
HN
N
OH
Me
Me
O
TBS
CO2tBu
S-5
8.0 7.5 7.0 6.5 6.0 5.5 5.0 4.5 4.0 3.5 3.0 2.5 2.0 1.5 1.0 0.5 0.0 ppm

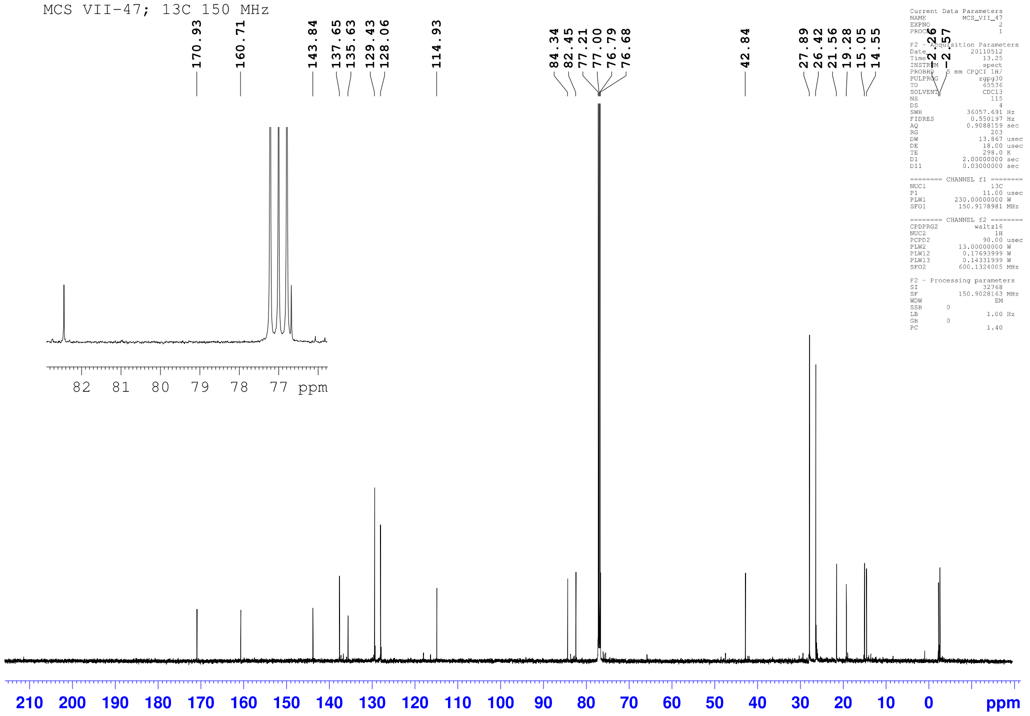
MCS VII-47; 13C 150 MHz
170.93
160.71
143.84
137.65
135.63
129.43
128.06
114.93
84.34
82.45
77.21
77.00
76.79
76.68
42.84
27.89
26.42
21.56
19.28
15.05
14.55
82 81 80 79 78 77 ppm
210 200 190 180 170 160 150 140 130 120 110 100 90 80 70 60 50 40 30 20 10 0 ppm

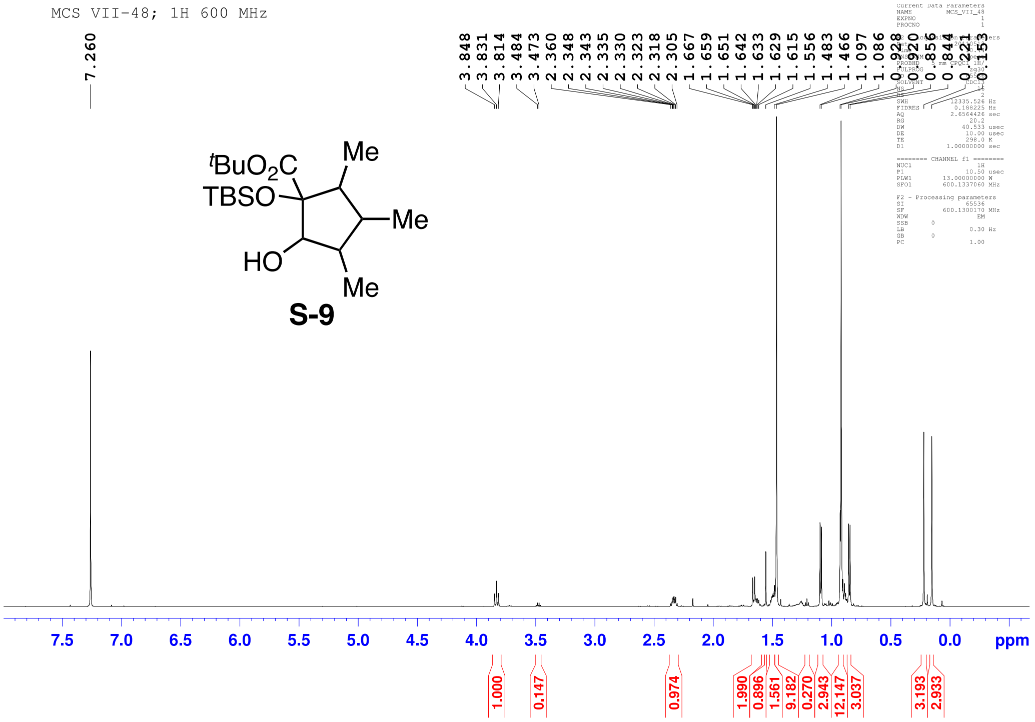
MCS VII-48; 1H 600 MHz
7.260
3.848 3.831 3.814 3.484 3.473 2.360 2.348 2.343 2.335 2.330 2.323 2.318 2.305 1.667 1.659 1.651 1.642 1.633 1.629 1.615 1.556 1.483 1.466 1.097 1.086 0.928 0.920 0.856 0.844 0.221 0.153
^{t}BuO$_2$C
TBSO
HO
Me
Me
Me
S-9
7.5 7.0 6.5 6.0 5.5 5.0 4.5 4.0 3.5 3.0 2.5 2.0 1.5 1.0 0.5 0.0 ppm
1.000 0.147 0.974 1.990 0.896 1.561 9.182 0.270 2.943 12.147 3.037 3.193 2.933

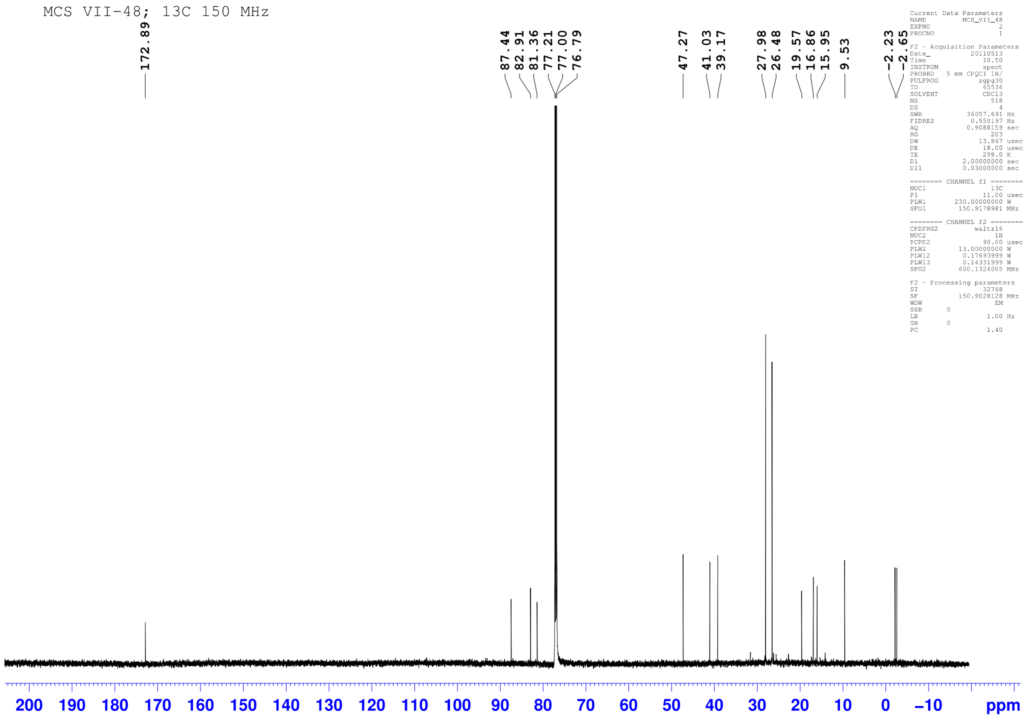
MCS VII-48; 13C 150 MHz
172.89
87.44 82.91 81.36 77.21 77.00 76.79
47.27 41.03 39.17 27.98 26.48 19.57 16.86 15.95 9.53 −2.23 −2.65
200 190 180 170 160 150 140 130 120 110 100 90 80 70 60 50 40 30 20 10 0 −10 ppm
